# Seasonal Variability of Volatile Components in *Calypogeia integristipula*

**DOI:** 10.3390/molecules28217276

**Published:** 2023-10-26

**Authors:** Rafał Wawrzyniak, Małgorzata Guzowska, Wiesław Wasiak, Beata Jasiewicz, Alina Bączkiewicz, Katarzyna Buczkowska

**Affiliations:** 1 Faculty of Chemistry, Adam Mickiewicz University in Poznań, Uniwersytetu Poznańskiego 8, 61-614 Poznań, Poland; malguz@amu.edu.pl (M.G.); wasiakw@amu.edu.pl (W.W.); beatakoz@amu.edu.pl (B.J.); 2 Faculty of Biology, Adam Mickiewicz University in Poznań, Uniwersytetu Poznańskiego 6, 61-614 Poznań, Poland; alinbacz@amu.edu.pl (A.B.); androsac@amu.edu.pl (K.B.)

**Keywords:** *Calypogeia integristipula*, volatile organic compounds, liverworts, HS-SPME, GC-MS

## Abstract

Liverworts contain a large number of biologically active compounds that are synthesised and stored in their oil bodies. However, knowledge about the chemical composition of individual species is still incomplete. The subject of the study was *Calypogeia integristipula*, a species representing leafy liverworts. Plant material for chemotaxonomic studies was collected from various locations in Poland. The chemical composition was determined in 74 samples collected from the natural environment in 2021 and 2022 in three growing seasons: spring, summer and autumn, and for comparison with samples originating from in vitro culture. The plants were classified as *Calypogeia integristipula* on the basis of morphological characteristics, oil bodies, and DNA markers. The volatile organic compounds (VOCs) from the biological material were extracted by headspace solid phase microextraction (HS-SPME). The samples were then analysed by gas chromatography–mass spectrometry (GC-MS). A total of 79 compounds were detected, of which 44 compounds were identified. The remaining compounds were described using the MS fragmentation spectrum. Cyclical changes in the composition of compounds associated with the growing season of *Calypogeia integristipula* were observed. Moreover, samples from in vitro culture and samples taken from the natural environment were shown to differ in the composition of chemical compounds. In terms of quantity, among the volatile compounds, compounds belonging to the sesquiterpene group (46.54–71.19%) and sesqiuterpenoid (8.12–22.11%) dominate. A smaller number of compounds belong to aromatic compounds (2.30–10.96%), monoterpenes (0.01–0.07%), monoterpenoids (0.02–0.33%), and aliphatic hydrocarbons (1.11–6.12%). The dominant compounds in the analysed liverworts were: anastreptene (15.27–31.14%); bicyclogermacrene (6.99–18.09%), 4,5,9,10-dehydro-isolongifolene (2.00–8.72%), palustrol (4.95–9.94%), spathulenol (0.44–5.11%).

## 1. Introduction

Phytochemical studies have shown that liverworts have a large number of biologically active compounds, such as terpenoids and aromatic compounds, and some of them can be important for humans. Many of the biologically active compounds are unique to this group of plants [1,2,3,4,5]. Liverworts are the only group of plants with characteristic cellular structures, oil bodies, that are organelles surrounded by a single membrane [6,7]. In these structures, chemical compounds are synthesised and stored [8,9]. Chemotaxonomic studies of liverworts have shown the presence of specific compounds at the family, genus, and even species level [9,10,11]. Furthermore, chemical studies of complex species, e.g., *Conocephalum conicum* and *Anaura pinguis*, revealed marker compounds enabling the identification of cryptic species [12,13]. Studies conducted on various plant species have shown that the accumulation of specialised metabolites depends on various environmental factors, including temperature, light, and soil moisture [14]. In the higher plant studied so far, such as *Thymus* [15], *Rosmarinus* [16,17], *Helichrysum* [18], *Mentha* [19], and *Eugenia patrisii* [20], variability in quantity or composition of essential oil content related to the season or different environmental conditions has also been demonstrated. Similarly, seasonal changes in the composition of volatile organic compounds (VOCs) were observed in one of the species of the genus *Calypogeia*, that is, in the previously analysed *Calypogeia azurea* [21].

The genus *Calypogeia* Raddi represents leafy liverworts belonging to the subclass Jungermanniidae. The genus comprises about 90 species [22,23]. This genus occurs almost worldwide, but the highest diversity of species was recorded in subtropical and tropical climates [24,25]. In the Holarctic, the species richness of *Calypogeia* is much lower, with nine species occurring in Europe, including *Calypogeia integristipula* Steph. [23]. *Calypogeia integristipula* is a small plant that grows in mats or mixed with other bryophytes. Its shoots are up to 3 cm long and 1.5–3 mm wide. The species has a wide distribution in the northern hemisphere and has been recorded in North America, Europe, and Asia [26], however, the distribution is uncertain, as *Calypogeia integristipula* can sometimes be confused with *Calypogeia neesiana*, to which it is most morphologically similar [27]. The characteristics that distinguish these two species are the shape of the leaves and the underleaves, but these morphological characteristics can be subject to environmental modification. The most stable morphological diagnostic feature is the distribution and shape of the oil bodies [25,26]. However, in recent times, species identification has often been performed based on molecular tools, including the fast-growing DNA barcoding proposed by [28]. Another method to identify taxonomically difficult species of liverworts is an analysis of the chemical composition of the compounds contained in plants [8,12,13]. Chemical composition analysis is a method for classifying and identifying plants based on confirmed differences and similarities in their biochemical composition. Chemotaxonomic analysis in the case of liverworts gives reproducible results with a low probability of error. However, to use chemical analysis for species identification, it is necessary to know whether the qualitative and quantitative composition of chemical compounds is constant for the given species collected from different substrates or at different times of the growing season. The aim of the work was to analyse the composition of chemical compounds in *Calypogeia integristipula* collected at different times of the growing season to indicate the compounds that are characteristic of the tested species. We also wanted to obtain information whether in vitro cultures conducted in laboratory conditions allow obtaining plant material with a similar composition of chemical compounds as in material originating from the natural environment.

## 2. Results and Discussion

### 2.1. Volatiles Present in Calypogeia integristipula

Thirty-eight samples of *Calypogeia integristipula* collected in 2022 in different vegetation seasons: spring (May), summer (July), and autumn (September) (Table 1) have been analysed for volatile organic compounds. The results of chemical composition for samples collected from the natural environment are presented in the following tables: Table 2a,b for samples CI-1 to CI-12 collected in spring, Table 3a,b for samples CI-13 to CI-24 collected in summer, Table 4a,b for samples CI-25 to CI-36 collected in autumn, and Table 5 for samples CI-37 and CI-38 from in vitro culture. In the Supplementary Materials, Table S1 contains information on samples collected in 2021. The results of chemical composition analyses for these samples are presented in Tables S2a,b, S3a,b and S4a,b.

A total of 79 compounds were detected of which 44 were identified, accounting for 96.01–99.71% of the total volatile compositions. The remaining compounds are described by means of mass spectra. In terms of content, compounds belonging to sesquiterpenes (46.54–71.19%) and sesqiuterpenoids (10.82–22.11%) dominate. In addition to the groups of compounds, compounds belonging to aromatic compounds (3.34–10.51%), monoterpenes (0.01–0.07%), monoterpenoids (0.02–0.33%), and aliphatic hydrocarbons (1.11–6.12%) were detected in *Calypogeia integristipula* cells. In *Calypogeia integristipula*, the dominant compounds are anastreptene (**23**) (15.28–31.14%), bicyclegermacrene (**45**) (6.99–17.72%), 4,5,9,10-dehydro-isolongifolene (**53**) (4.14–8.72%), palustrol (**59**) (5.09–9.94%), and spathulenol (**62**) (2.57–7.97%).

To better illustrate the seasonal variability in the composition of the determined volatile organic compounds, Table 6 includes average values for individual seasons obtained on the basis of the results presented in Table 2, Table 3, Table 4 and Table 5.

Liverworts of the *Calypogeia integristipula* species are characterised by visible variability in the composition of specialised metabolites, resulting from the vegetation period of the plant. Cyclical changes in the composition of VOCs were observed in spring, summer, and autumn, repeating from 2021–2022. Furthermore, clear differences in terms of chemical compound composition, both quantitative and qualitative, were observed between samples from in vitro culture and samples from the natural environment (Table 6). The diversity of chemical composition observed in *Calypogeia integristipula* is not an isolated case, because in addition to *Calypogeia azurea* liverworts [21], it has also been described in the example of *Hypnum cupressiforme* moss [29].

**Figure 1:**
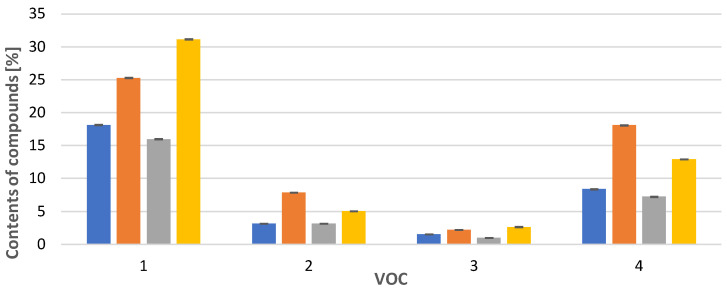
Comparison of VOC percentages for samples collected in 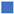 spring, 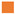 summer, 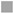 autumn and obtained 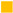 in vitro. Compounds: 1: anastreptene (**23**), 2: aromandendrene (**34**), 3: δ-selinene (**42**), 4: bicyclogermacrene (**45**). The deviation bar shows the standard deviation for a given group.

**Figure 2:**
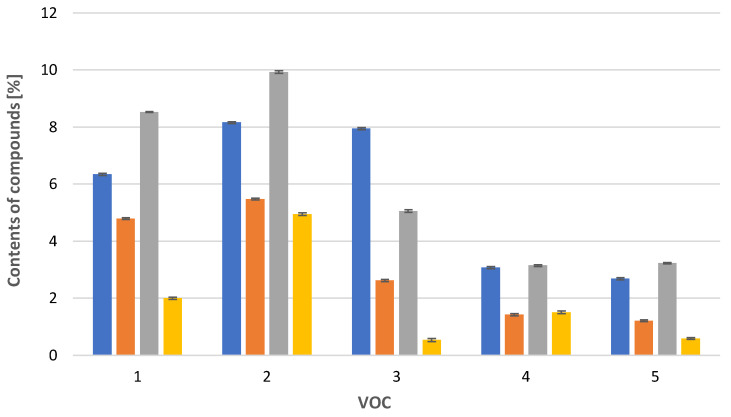
Comparison of VOC percentages for samples collected in 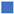 spring, 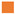 summer, 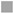 autumn and obtained 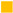 in vitro. 1: 4,5,9,10-dehydro-isolongifolene (**53**), 2: palustrol (**59**), 3: spathulenol (**62**), 4: globulol (**65**), 5: 1,4-dimethyl-7-(1-methylethyl)-azulene (**78**). The deviation bar shows the standard deviation for a given group.

In the case of *Calypogeia integristipula* in summer, the content of anastraptene (**23**)**,** aromandendrene (**34**), δ-selinene (**42**), and bicyclogermacrene (**45**) was higher than in spring and autumn. The dependencies discussed are shown in Figure 1. On the other hand, samples collected in spring and autumn are characterised by a higher content of 4,5,9,10-dehydro-isolongifolene (**53**), palustrol (**59**), spathulenol (**62**), globulol (**65**), and 1,4-dimethyl-7-(1-methylethyl)-azulene (**78**) (Figure 2).

The relative content of volatile organic compounds increases from spring to summer, reaching the highest value in the summer months. Undoubtedly, this may be due to the fact that in the summer liverworts are exposed to a smaller supply of water and to greater insolation. Most sesquiterpenoids increase from spring to peak in summer. The levels of these compounds are likely to increase in summer to allow liverworts to cope with abiotic stresses such as high temperatures and drought. A similar correlation was observed in the liverwort *Calypogeia azurea*, but the seasonal changes involved compounds belonging to sesquiterpenes [21].

When comparing samples of *Calypogeia integristipula* collected in nature with plants obtained from in vitro cultures, it should be concluded that samples from in vitro cultures were similar in the composition of specialised metabolites to plant samples collected in summer. However, they differ from samples collected in the natural environment with a higher content of aliphatic and aromatic hydrocarbons, such as: pentanal (**2**), hexanal (**3**), benzenemethanol (**9**), phenoxyethanol (**16**). Exactly the same situation was observed for in vitro samples of *Calypogeia azurea* liverworts [21], their composition was the same as the samples collected during the summer. However, in vitro samples of *Calypogeia azurea* liverworts did not contain elevated levels of aliphatic and aromatic compounds. However, liverworts do not always show variability in the composition of metabolites depending on environmental factors. An example would be liverworts belonging to the species *Aneura pinguis*, which do not show such variability [13]. The factor that differentiates them is their structure. *Calypogeia* are liverworts with a leafy structure and *Aneura pingius* has a thallus-like structure, making it more resistant to changes in environmental factors.

### 2.2. Statistical Analysis of the Obtained Results

**Figure 3:**
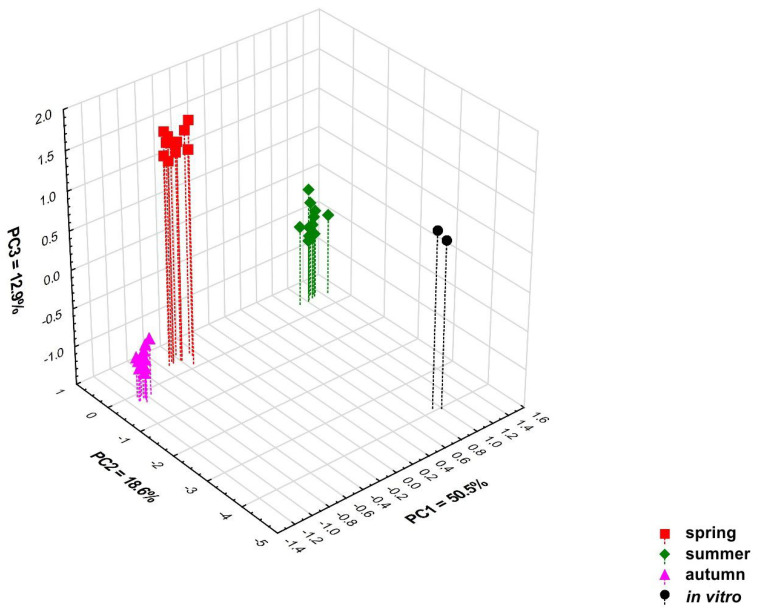
Three-dimensional PCA scatter plot based on all 79 detected compounds in samples of *Calypogeia integristipula* collected in spring, summer, and autumn in 2022 and in vitro. The percentage of explained variance (R^2^X) is 50.5% for PC1, 18.6% for PC2, 12.9% for PC3, and predictive ability (Q^2^) is 46.7%, 15.3%, and 36.0%, respectively.

Multivariate classification analyses (PCA and heat map) performed on all 79 detected chemical compounds confirm the differentiation of the content of these compounds in the tested samples depending on the time at which the samples were collected. Four significant principal components (PCs) included in the PCA model explain 84.4% of the variation (R^2^X) and 71.0% of the predicted variation (Q^2^). Based on the value of the predictive variability Q^2^, it was determined that the optimal number of principal components in the model is three. The 3D scatterplot revealed the presence of four distinct groups correlated with the collection time (Figure 3 and Figure S7). The PC1 axis divided the samples collected in spring and autumn from the samples collected in summer, and these originated from the in vitro culture. The second principal component (PC2 axis) distinguishes in vitro culture samples from all others, while samples collected in spring and autumn are very well differentiated by the PC3 axis (Figure 3, Figures S1–S3 and S8–S10). It should be emphasised that the variation between individual samples from different regions collected in the same growing season is low (Figure 3, Figures S1–S3 and S8–S10).

Samples collected in spring and autumn, samples collected in summer, and those originating from in vitro culture differ mainly in the content of compounds **1**, **10**, **23**, **30**, **42**, **45**, **52**, **59**, **65**, **67**, and **78**, which have the largest contribution to the PC1 axis. Variables **23**, **42**, and **45** had positive loading, whereas **1**, **10**, **30**, **52**, **59**, **65**, **67**, and **78** had negative loading. Samples collected in spring and autumn have a lower concentration of anastreptene (**23**), δ-selinene (**42**), and bicyclogermacrene (**45**) than those collected in summer and from in vitro culture (Table 7). Compounds **2**, **3**, **9**, **16**, **39**, and **57** distinguish samples grown in in vitro culture as the compounds have the highest loadings in PC2. Variable **39** had positive loading, **2**, **3**, **9**, **16**, and **57** were negatively loaded. Culture plants were characterised by lower concentrations of the compound **39** (not identified) and a higher content of pentanal (**2**), hexanal (**3**), benzenemethanol (**9**), phenoxyethanol (**16**), and compound **57** (not identified) than in plants growing under natural conditions. The samples collected in spring and autumn differ in the content of compounds **4** (positive loading), **13**, and **76** (negative loading), which contribute greatly to PC3. The plants collected in spring had a higher content of 1-hexanol (**4**), while the concentration of benzeneethanol (**13**) and germacra-4(15),5,10(14)-trien-1-α-ol (**76**) was lower than in those collected in autumn (Table 7). The loading plots showing the contribution of individual variables to the first three principal components are presented in Figures S4–S6 and S11–S13.

**Figure 4:**
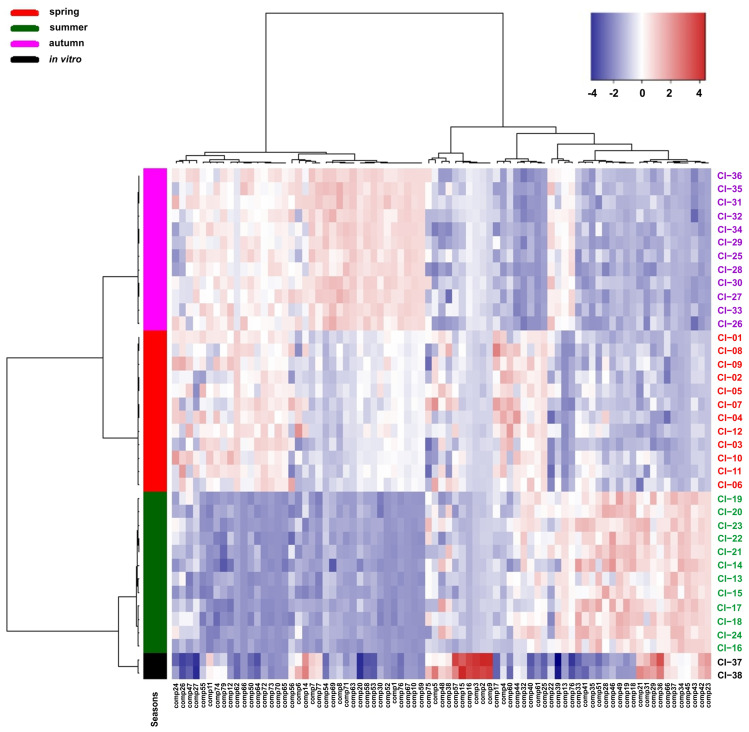
Clustering and heat map analysis of the 79 chemical compounds detected in the *Calypogeia integristipula* samples studied collected in different seasons in 2022 and from in vitro culture. The annotation bar on the left of the heat map shows the clustering of the samples by season. Each cell was coloured on the basis of the level of the chemical compound in the sample; red is used for positive values, while blue is used for negative values (data are standardised).

Similarly, the differentiation of the samples analysed according to the growing season was shown by the heat map. The analysed samples are grouped into two main clusters correlated with the date of their collection. The first cluster includes samples collected in spring and autumn, which form separate groups, and the second group includes samples collected in summer and from in vitro culture. The analysis of the heat map showed that the chemical compounds detected in the *Calypogeia integristipula* samples analysed form two separate groups, the content of which in the plants tested changes clearly depending on the time of collection of the material in the field (Figure 4).

## 3. Materials and Methods

### 3.1. Plant Material

The plant material studied included 74 *Calypogeia integristipula* samples obtained from habitats in different regions of Poland and cultured in vitro. Detailed information on the plant material used in the investigation is presented in Table 1 and Table S1. The data include the place of collection, geographic coordinates determined using a GPS receiver, type of sample (whether the sample was analysed directly from a natural site or in vitro), and the date of collection of the plant material. In Table 1 and Table S1, the samples are arranged by season and within a season by geographical location from south to north.

Natural liverwort samples were collected in the years 2021–2022 in 3 seasons: spring (May), summer (July), and autumn (September). The months of material collection were selected according to the growing season in Poland. The first samples were taken in spring to give the plants time to regenerate after winter, and then the samples were taken every two months. No plant material collection was carried out in the winter months due to snow cover and negative temperatures. Samples from the natural environment were taken from the same substrate at all sites, i.e., a layer of humus on which *Calypogeia integristipula* often grows [22,27]. The in vitro samples were collected in 2022 and 2023. The samples were taken when the plants were fully developed, at the optimal stage of their development. Both plants collected in the natural environment and in in vitro culture were at the same stage of development, i.e., the samples consisted of well-developed stems that were in a sterile state, i.e., without reproductive structures.

Research materials were collected at 9 locations in the Bieszczady Mts, Tatra Mts, Małe Pieniny Mts, Pieniny Mts, Gorce Mts, central Poland (Wielkopolska), west Poland (Lubuskie), Suwałki Lake District, and Pomerania. Five samples weighing approximately 15 g each were taken from each natural site. Only green plants that did not show signs of drying out and were not affected by visible diseases were eligible for collection and further research. All samples analysed were determined based on morphological characteristics [22,27], oil bodies, and genetically by four DNA barcodes (*rbcL*, *trnL*, *trnG*, and *trnH-psbA*). The sequences of the analysed samples corresponded to the sequences of *Calypogeia integristipula* with GenBank acc. numbers: JF776848-JF776849, MH367760-MH367761, MH367632-MH367635, MH367823-MH367826, MH367697-MH367697, deposited by Buczkowska et al. [30].

Before analysis, the samples were cleaned from different plant material and soil. In addition to samples from natural sites, in vitro culture samples were also analysed.

### 3.2. HS-SPME Extraction

Volatile compounds from *Calypogeia integristipula* were extracted using the headspace solid phase microextraction (HS-SPME) technique. Fused silica fibers coated with divinylbenzene/carboxen/polydimethylsiloxane (DVB/CAR/PDMS) were used and 2 cm long fiber covered with a 50/30 µm thick film was used. To prepare the fibers for analysis, they were conditioned for one hour at 270 °C, in accordance with the manufacturer’s guidelines. For the extraction process, 5 mg of plant material was placed within a 1.7 mL vial, which was hermetically sealed using a PTFE/red silicone septum. The extraction of the compounds followed at 50 °C for 60 min. Fiber analyte desorption was carried out in the injection port of the gas chromatograph at 250 °C for 10 min. Both the sorption and desorption operations were performed using the TriPlus RSH autosampler (Thermo Scientific, Waltham, MA, USA).

### 3.3. GC-MS Analysis

The analysis of volatile compounds was performed using a previously described gas chromatography–mass spectrometry (GC-MS) method [13,21]. GC-MS analyses using a silphenylene phase were performed on a Trace 1310 (Thermo Scientific, Waltham, MA, USA) equipped with a Quadrex 007-5MS column (30 m, 0.25 mm, 0.25 μm).

The ISQ QD mass detector (Thermo Scientific, Waltham, MA, USA) was operated at 70 eV in the EI mode in the m/z range of 30 to 550. Helium was employed as the carrier gas, flowing at a rate of 1.0 mL/min. The oven temperature was programmed from 60 to 230 °C at 4 °C/min and then isothermal at 230 °C for 40 min. The injector temp and transfer line were 250 °C. Injection samples were in splitless mode with a dedicated liner for the SPME technique. To confirm the identity of the components, their mass spectral fragmentation patterns were compared with those stored in various mass spectrometry databases (including NIST 2011 [31], NIST Chemistry WebBook [32], Adams 4 Library [33], and Pherobase [34]). Furthermore, the retention indices in non-polar columns, determined relative to a homologous series of n-alkanes (C8–C26), were compared with the data from the published indices. Quantitative data of the components were obtained by integrating the TIC chromatogram and calculating the relative percentage of the peak areas. Each sample of *Calypogeia integristipula* was analysed three times.

### 3.4. Statistical Analysis

In multivariate statistical analyses, the data obtained for *Calypogeia integristipula* in different vegetative seasons and growing in in vitro cultures were compared to check whether the composition of chemical compounds in the species studied is constant or changes depending on the season when the samples were collected in the field. The PCA analysis based on the correlation matrix of all 79 compounds using the non-linear iterative partial least squares (NIPALS) algorithm was used to construct the PCA model. The v-fold method (v = 7) was used to find the optimal number of principal components that reaches the maximum Q^2^. The statistical significance of the principal component was assessed based on the following rule: Q^2^ > limit. This analysis was performed with STATISTICA 13.3 (StatSoft, Kraków, Poland). To compare the content of chemical compounds in individual samples collected in different growing seasons, from in vitro and different geographic regions, the data were illustrated using a heat map, which allows the grouping of objects and variables simultaneously. In a heat map, two-dimensional variables (sample, chemical compounds) are represented by colours. The heat map is a common technique in biology that is useful for visualising multivariate data [35,36]. A heat map with dendrograms for both variables and row-side season annotation was generated using the heatmap3 package in R [37,38]. We used the following parameters: standardisation for columns, Euclidean and 1-r Pearson distances for rows and columns, respectively, and Ward agglomeration method for clustering.

## 4. Conclusions

GC-MS analysis of volatiles isolated by SPME from *Calypogeia integristipula* liverwort cells showed 79 volatile compounds that are metabolites. Forty-four of them have been identified. Our research has shown that the composition of metabolites is dominated by compounds belonging to sesquiterpenes, sesquiterpenoids, and aromatic compounds. The dominant compounds are anastreptene, bicyclogermacrene, 4,5,9,10-dehydro-isolongifolene, palustrol, and spathulenol. The composition of the metabolites was found to be unaffected by geographical region. However, during the identification, the variability of the composition resulting from the sensitivity of this species to environmental stress should be taken into account. It is manifested by cyclic changes in the content of metabolites depending on the vegetation period of the plant: spring–summer–autumn. Clear differences in the composition of chemical compounds were also observed depending on whether the plants were collected from the natural environment or obtained from in vitro culture. The in vitro culture in terms of the composition of metabolites was found to be more similar to the environmental samples collected in the summer. Multidimensional PCA statistical analyses and heat map clustering confirmed the relationships described above.

## Figures and Tables

**Table 1:** The *Calypogeia integristipula* sampling data in 2022 used for studies, divided by collecting season.

Sample Code	Collection Place	Geographical Coordinates	Date Month Year
	Spring		
CI-1	South-Eastern Poland, Bieszczady Mts, Rozsypaniec	49°03′35.5″ N, 22°46′10.7″ E	May 2022
CI-2	Southern Poland, Tatry Mts, Morskie Oko	49°12′00.0″ N, 20°04′24.3″ E	May 2022
CI-3	Southern Poland, Tatry Mts, Gąsienicowa Valley	49°14′45.8″ N, 20°00′25.1″ E	May 2022
CI-4	Southern Poland, Tatry Mts, Kościeliska Valley	49°15′18.3″ N, 19°51′55.4″ E	May 2022
CI-5	Southern Poland, Małe Pieniny Mts	49°22′56.8″ N, 20°33′18.9″ E	May 2022
CI-6	Southern Poland, Pieniny Mts, Potok Kotłowy stream	49°24′22.5″ N, 20°24′02.1″ E	May 2022
CI-7	Southern Poland, Gorce Mts, Ochotnica Dolna	49°32′04.5″ N, 20°19′00.0″ E	May 2022
CI-8	Central Poland, Wielkopolska, Antonin	51°30′59.8″ N, 17°50′58.1″ E	May 2022
CI-9	Western Poland, Lubuskie, Nabłoto	51°47′31.4″ N, 14°46′55.8″ E	May 2022
CI-10	North-Eastern Poland, Suwałki Lake District, Lake Łempis	54°02′38.2″ N, 23°28′10.8″ E	May 2022
CI-11	North-Western Poland, Pomerania, Lake Czarne	54°22′50.7″ N, 18°12′07.6″ E	May 2022
CI-12	North-Western Poland, Pomerania, Lake Lubygość	54°24′46.7″ N, 17°58′44.0″ E	May 2022
	Summer		
CI-13	South-Eastern Poland, Bieszczady Mts, Rozsypaniec	49°03′35.5″ N, 22°46′10.7″ E	July 2022
CI-14	Southern Poland, Tatry Mts, Morskie Oko	49°12′00.0″ N, 20°04′24.3″ E	July 2022
CI-15	Southern Poland, Tatry Mts, Gąsienicowa Valley	49°14′45.8″ N, 20°00′25.1″ E	July 2022
CI-16	Southern Poland, Tatry Mts, Kościeliska Valley	49°15′18.3″ N, 19°51′55.4″ E	July 2022
CI-17	Southern Poland, Małe Pieniny Mts	49°22′56.8″ N, 20°33′18.9″ E	July 2022
CI-18	Southern Poland, Pieniny Mts, Potok Kotłowy Stream	49°24′22.5″ N, 20°24′02.1″ E	July 2022
CI-19	Southern Poland, Gorce Mts, Ochotnica Dolna	49°32′04.5″ N, 20°19′00.0″ E	July 2022
CI-20	Central Poland, Wielkopolska, Antonin	51°30′59.8″ N, 17°50′58.1″ E	July 2022
CI-21	Western Poland, Lubuskie, Nabłoto	51°47′31.4″ N, 14°46′55.8″ E	July 2022
CI-22	North-Eastern Poland, Suwałki Lake District, Lake Łempis	54°02′38.2″ N, 23°28′10.8″ E	July 2022
CI-23	North-Western Poland, Pomerania, Lake Czarne	54°22′50.7″ N, 18°12′07.6″ E	July 2022
CI-24	North-Western Poland, Pomerania, Lake Lubygość	54°24′46.7″ N, 17°58′44.0″ E	July 2022
	Autumn		
CI-25	South-Eastern Poland, Bieszczady Mts, Rozsypaniec	49°03′35.5″ N, 22°46′10.7″ E	September 2022
CI-26	Southern Poland, Tatry Mts, Morskie Oko	49°12′00.0″ N, 20°04′24.3″ E	September 2022
CI-27	Southern Poland, Tatry Mts, Gąsienicowa Valley	49°14′45.8″ N, 20°00′25.1″ E	September 2022
CI-28	Southern Poland, Tatry Mts, Kościeliska Valley	49°15′18.3″ N, 19°51′55.4″ E	September 2022
CI-29	Southern Poland, Małe Pieniny Mts	49°22′56.8″ N, 20°33′18.9″ E	September 2022
CI-30	Southern Poland, Pieniny Mts, Potok Kotłowy Stream	49°24′22.5″ N, 20°24′02.1″ E	September 2022
CI-31	Southern Poland, Gorce Mts, Ochotnica Dolna	49°32′04.5″ N, 20°19′00.0″ E	September 2022
CI-32	Central Poland, Wielkopolska, Antonin	51°30′59.8″ N, 17°50′58.1″ E	September 2022
CI-33	Western Poland, Lubuskie, Nabłoto	51°47′31.4″ N, 14°46′55.8″ E	September 2022
CI-34	North-Eastern Poland, Suwałki Lake District, Lake Łempis	54°02′38.2″ N, 23°28′10.8″ E	September 2022
CI-35	North-Western Poland, Pomerania, Lake Czarne	54°22′50.7″ N, 18°12′07.6″ E	September 2022
CI-36	North-Western Poland, Pomerania, Lake Lubygość	54°24′46.7″ N, 17°58′44.0″ E	September 2022
	In vitro		
CI-37	North-Western Poland, Pomerania, Lake Lubygość	54°24′46.7″ N, 17°58′44.0″ E	March 2022
CI-38	North-Western Poland, Pomerania, Lake Lubygość	54°24′46.7″ N, 17°58′44.0″ E	March 2023

**Table 2:** (**a**) Volatile compounds detected in the samples collected in spring (CI-1–CI-6). (**b**) Volatile compounds detected in the samples collected in spring (CI-7–CI-12).

(a)									
No.	Compounds *	RI **	RI ***	Code ****					
				CI-1	CI-2	CI-3	CI-4	CI-5	CI-6
1	propan-1-ol	<700 ^a,b^	483 ^a^	1.11 (0.02)	1.13 (0.03)	1.17 (0.02)	1.15 (0.03)	1.09 (0.03)	0.99 (0.05)
2	pentanal	705 ^a,b,c,d^	704 ^c^	0.38 (0.03)	0.32 (0.05)	0.38 (0.03)	0.35 (0.04)	0.25 (0.04)	0.34 (0.04)
3	hexanal	802 ^a,b,c,d^	801 ^c^	0.18 (0.02)	0.23 (0.03)	0.18 (0.04)	0.21 (0.02)	0.22 (0.03)	0.19 (0.03)
4	hexan-1-ol	867 ^a,b^	869 ^a^	0.49 (0.05)	0.55 (0.05)	0.51 (0.02)	0.56 (0.04)	0.47 (0.04)	0.41 (0.04)
5	heptanal	902 ^a,b,c,d^	901 ^c^	0.06 (0.01)	0.07 (0.02)	0.02 (0.01)	0.03 (0.01)	0.06 (0.02)	0.09 (0.02)
6	α-pinene	939 ^a,b,c^	932 ^c^	0.02 (0.01)	0.03 (0.01)	0.01 (0.01)	0.05 (0.01)	0.03 (0.01)	0.02 (0.01)
7	benzaldehyde	940 ^a,b,c^	952 ^c^	0.18 (0.03)	0.20 (0.06)	0.15 (0.02)	0.22 (0.03)	0.21 (0.03)	0.17 (0.05)
8	2-ethylhexan-1-ol	1023 ^a,b^	1025 ^a^	0.08 (0.02)	0.09 (0.02)	0.05 (0.01)	0.06 (0.02)	0.10 (0.04)	0.11 (0.03)
9	phenylmethanol	1028 ^a,b,c^	1026 ^c^	1.11 (0.03)	1.17 (0.06)	1.21 (0.04)	1.05 (0.04)	1.13 (0.03)	1.09 (0.03)
10	phenylacetaldehyde	1044 ^a,b^	1044 ^a^	1.18 (0.03)	1.23 (0.03)	1.15 (0.04)	1.12 (0.05)	1.19 (0.04)	1.22 (0.04)
11	nonanal	1102 ^a,b,c,d^	1100 ^c^	0.12 (0.01)	0.08 (0.05)	0.13 (0.03)	0.09 (0.02)	0.10 (0.03)	0.11 (0.02)
12	3,4-dimethylcyclohexan-1-ol	1115 ^a,b^	1126 ^a^	0.09 (0.03)	0.08 (0.03)	0.11 (0.03)	0.07 (0.02)	0.09 (0.03)	0.07 (0.03)
13	phenylethanol	1121 ^a,b^	1121 ^a^	0.16 (0.02)	0.13 (0.06)	0.12 (0.01)	0.15 (0.03)	0.13 (0.04)	0.16 (0.04)
14	decanal	1195 ^a,b,c,d^	1201 ^c^	0.07 (0.02)	0.05 (0.01)	0.03 (0.01)	0.05 (0.02)	0.06 (0.02)	0.04 (0.02)
15	β-cyclocitral	1221 ^c^	1217 ^c^	0.08 (0.01)	0.07 (0.01)	0.07 (0.02)	0.06 (0.02)	0.05 (0.01)	0.08 (0.03)
16	2-phenoxyethan-1-ol	1225 ^a,b^	1226 ^a^	0.95 (0.51)	0.87 (0.05)	0.99 (0.03)	1.01 (0.03)	0.92 (0.05)	0.88 (0.05)
17	bicycloelemene	1316 ^a^	1330 ^a^	0.14 (0.02)	0.10 (0.03)	0.08 (0.02)	0.13 (0.04)	0.11 (0.03)	0.09 (0.02)
18	δ-elemene	1324 ^a,b,c^	1335 ^c^	0.74 (0.04)	0.77 (0.02)	0.71 (0.03)	0.78 (0.03)	0.82 (0.04)	0.83 (0.05)
19	204[M+](5) 121(100) 93(89)	1343	ND	0.18 (0.02)	0.19 (0.03)	0.15 (0.03)	0.21 (0.04)	0.19 (0.04)	0.23 (0.04)
20	200[M+](39) 159(100) 117(95)	1345	ND	1.09 (0.05)	1.07 (0.05)	0.99 (0.04)	1.04 (0.04)	1.11 (0.06)	1.05 (0.03)
21	202[M+](13) 81(100) 96(73)	1350	ND	0.11 (0.01)	0.09 (0.01)	0.12 (0.02)	0.14 (0.02)	0.08 (0.02)	0.06 (0.02)
22	204[M+](10) 119(100) 91(84)	1353	ND	0.08 (0.01)	0.05 (0.02)	0.04 (0.01)	0.03 (0.01)	0.09 (0.02)	0.04 (0.02)
23	anastreptene	1370 ^a^	1370 ^a^	17.99 (0.06)	18.13 (0.05)	18.10 (0.06)	17.92 (0.05)	18.19 (0.06)	18.21 (0.05)
24	204[M+](5) 81(100) 93(96)	1384	ND	0.21 (0.02)	0.18 (0.02)	0.15 (0.05)	0.23 (0.04)	0.19 (0.02)	0.17 (0.05)
25	β-elemene	1391 ^a,b,c^	1389 ^c^	2.71 (0.01)	2.81 (0.04)	2.72 (0.03)	2.76 (0.04)	2.83 (0.03)	2.76 (0.03)
26	204[M+](13) 157(100) 185(84)	1398	ND	0.29 (0.02)	0.25 (0.05)	0.18 (0.02)	0.32 (0.03)	0.27 (0.04)	0.32 (0.02)
27	204[M+](13) 157(100) 185(84)	1417	ND	0.34 (0.03)	0.21 (0.02)	0.23 (0.03)	0.37 (0.05)	0.18 (0.02)	0.24 (0.04)
28	204[M+](19) 135(100) 105(82)	1423	ND	0.17 (0.01)	0.25 (0.04)	0.15 (0.05)	0.31 (0.05)	0.22 (0.04)	0.15 (0.02)
29	204[M+](9) 91(100) 105(93)	1425	ND	0.03 (0.01)	0.06 (0.03)	0.01 (0.01)	0.02 (0.01)	0.04 (0.01)	0.06 (0.02)
30	(-)-aristolene	1429 ^a,b,c,d^	1428 ^a^	1.02 (0.02)	1.12 (0.05)	0.99 (0.05)	1.05 (0.05)	1.18 (0.03)	1.06 (0.04)
31	204[M+](9) 107(100) 79(43)	1432	ND	0.15 (0.02)	0.18 (0.03)	0.16 (0.04)	0.16 (0.03)	0.20 (0.03)	0.19 (0.04)
32	γ-maaliene	1435 ^a,b^	1427 ^a^	0.49 (0.04)	0.55 (0.02)	0.47 (0.03)	0.51 (0.04)	0.50 (0.02)	0.47 (0.05)
33	α-maaliene	1443 ^a,b^	1442 ^a^	0.34 (0.04)	0.33 (0.04)	0.27 (0.03)	0.35 (0.03)	0.26 (0.04)	0.36 (0.02)
34	aromandendrene	1445 ^a,b^	1447 ^a^	3.25 (0.04)	3.14 (0.05)	3.18 (0.05)	3.28 (0.05)	3.02 (0.04)	3.06 (0.05)
35	selina-5,11-diene	1447 ^a,b^	1454 ^a^	0.47 (0.02)	0.59 (0.03)	0.45 (0.03)	0.42 (0.03)	0.63 (0.03)	0.58 (0.05)
36	dehydroaromadendrene	1456 ^c^	1460 ^c^	1.09 (0.04)	1.15 (0.02)	1.01 (0.04)	0.99 (0.05)	1.21 (0.03)	1.23 (0.05)
37	1,2,9,10-tetradehydroaristolane	1461	ND	0.47 (0.02)	0.46 (0.05)	0.40 (0.02)	0.43 (0.04)	0.50 (0.02)	0.51 (0.04)
38	204[M+](15) 91(100) 105(84)	1465	ND	0.39 (0.04)	0.31 (0.06)	0.33 (0.03)	0.30 (0.03)	0.41 (0.03)	0.43 (0.03)
39	204[M+](18) 128(100) 143(95)	1469	ND	0.32 (0.01)	0.33 (0.02)	0.29 (0.04)	0.26 (0.02)	0.36 (0.04)	0.37 (0.05)
40	γ-gurjunene	1474 ^c,d^	1475 ^c^	0.49 (0.04)	0.47 (0.04)	0.45 (0.05)	0.41 (0.03)	0.52 (0.02)	0.55 (0.03)
41	γ-muurolene	1477 ^c^	1478 ^c^	0.14 (0.02)	0.16 (0.04)	0.13 (0.02)	0.16 (0.02)	0.19 (0.01)	0.21 (0.05)
42	δ-selinene	1488 ^c^	1492 ^c^	1.36 (0.03)	1.54 (0.02)	1.29 (0.02)	1.35 (0.04)	1.37 (0.04)	1.29 (0.03)
43	ledene	1492 ^a,b,c^	1496 ^c^	1.59 (0.04)	1.65 (0.05)	1.61 (0.04)	1.59 (0.04)	1.55 (0.05)	1.71 (0.04)
44	204[M+](38) 105(100) 93(96)	1495	ND	0.19 (0.01)	0.31 (0.07)	0.15 (0.04)	0.41 (0.05)	0.17 (0.02)	0.27 (0.03)
45	bicyclogermacrene	1499 ^a,b,c^	1500 ^c^	8.41 (0.02)	8.38 (0.06)	8.34 (0.06)	8.43 (0.06)	8.25 (0.06)	8.51 (0.05)
46	204[M+](19) 93(100) 91(95)	1505	ND	0.09 (0.04)	0.19 (0.03)	0.14 (0.02)	0.19 (0.02)	0.21 (0.04)	0.24 (0.05)
47	202[M+](25) 133(100) 91(89)	1509	ND	0.21 (0.04)	0.18 (0.02)	0.19 (0.04)	0.15 (0.03)	0.24 (0.02)	0.20 (0.03)
48	206[M+](14) 191(100) 57(38)	1514	ND	0.26 (0.01)	0.21 (0.03)	0.26 (0.03)	0.17 (0.04)	0.29 (0.04)	0.20 (0.03)
49	202[M+](33) 131(100) 145(53)	1518	ND	0.18 (0.02)	0.15 (0.05)	0.14 (0.03)	0.21 (0.02)	0.19 (0.02)	0.17 (0.02)
50	δ-cadinene	1524 ^a,b,c^	1522 ^c^	0.33 (0.03)	0.30 (0.07)	0.30 (0.02)	0.32 (0.03)	0.29 (0.05)	0.27 (0.05)
51	204[M+](5) 91(100) 131(95)	1530	ND	0.09 (0.01)	0.08 (0.01)	0.09 (0.01)	0.11 (0.04)	0.10 (0.04)	0.10 (0.03)
52	200[M+](54) 185(100) 143(91)	1535	ND	0.17 (0.08)	0.13 (0.02)	0.15 (0.05)	0.16 (0.02)	0.11 (0.03)	0.10 (0.04)
53	4,5,9,10-dehydro-isolongifolene	1544 ^a,b^	1544 ^a^	6.12 (0.02)	6.34 (0.04)	6.34 (0.06)	6.29 (0.05)	6.01 (0.07)	6.40 (0.05)
54	202[M+](4) 128(100) 157(95)	1547	ND	0.69 (0.04)	0.70 (0.04)	0.69 (0.05)	0.73 (0.05)	0.75 (0.05)	0.69 (0.05)
55	200[M+](8) 171(100) 186(79)	1551	ND	0.22 (0.03)	0.09 (0.02)	0.23 (0.04)	0.25 (0.04)	0.25 (0.04)	0.14 (0.03)
56	200[M+](91) 129(100) 157(88)	1556	ND	0.09 (0.01)	0.05 (0.01)	0.11 (0.02)	0.09 (0.02)	0.11 (0.03)	0.15 (0.02)
57	204[M+](8) 143(100) 157(98)	1559	ND	0.05 (0.01)	0.08 (0.01)	0.04 (0.01)	0.07 (0.02)	0.09 (0.02)	0.11 (0.05)
58	204[M+](82) 173(100) 189(94)	1563	ND	1.38 (0.02)	1.45 (0.02)	1.41 (0.05)	1.33 (0.04)	1.45 (0.05)	1.41 (0.03)
59	palustrol	1567 ^c^	1567 ^c^	8.34 (0.02)	8.16 (0.03)	8.43 (0.04)	8.31 (0.04)	8.14 (0.05)	8.21 (0.06)
60	200[M+](11) 79(100) 93(95)	1570	ND	0.47 (0.04)	0.82 (0.02)	0.83 (0.03)	0.78 (0.03)	0.74 (0.03)	0.56 (0.03)
61	204[M+](31) 81(100) 109(88)	1573	ND	2.11 (0.11)	2.04 (0.03)	2.09 (0.02)	1.99 (0.02)	2.02 (0.02)	1.87 (0.02)
62	spathulenol	1576 ^a,b,c^	1577 ^c^	7.97 (0.04)	7.95 (0.04)	7.93 (0.04)	7.55 (0.04)	7.91 (0.06)	7.44 (0.06)
63	200[M+](56) 185(100) 143(63)	1581	ND	3.64 (0.05)	3.59 (0.04)	3.60 (0.02)	3.67 (0.04)	3.59 (0.04)	3.57 (0.02)
64	202[M+](4) 91(100) 79(82)	1587	ND	0.51 (0.03)	0.48 (0.05)	0.55 (0.05)	0.53 (0.02)	0.39 (0.02)	0.51 (0.05)
65	globulol	1599 ^a,b,c,d^	1590 ^c^	3.14 (0.02)	3.07 (0.04)	3.32 (0.03)	2.97 (0.02)	3.01 (0.04)	3.11 (0.04)
66	200[M+](8) 198(100) 183(84)	1605	ND	0.24 (0.02)	0.21 (0.02)	0.20 (0.04)	0.23 (0.04)	0.24 (0.03)	0.19 (0.02)
67	220[M+](2) 145(100) 200(93)	1609	ND	1.22 (0.02)	1.19 (0.02)	1.18 (0.04)	1.11 (0.05)	1.15 (0.02)	1.23 (0.05)
68	(+)-bisabola-2,10-diene[1,9]oxide	1615 ^a,b^	1596 ^a^	0.22 (0.03)	0.11 (0.03)	0.14 (0.03)	0.10 (0.03)	0.18 (0.03)	0.24 (0.04)
69	208[M+](3) 95(100) 85(95)	1621	ND	0.83 (0.05)	0.65 (0.04)	0.89 (0.05)	0.74 (0.05)	0.69 (0.04)	0.81 (0.04)
70	ledene oxide-(II)	1631 ^a,b^	1631 ^a^	0.22 (0.01)	0.24 (0.03)	0.28 (0.02)	0.19 (0.04)	0.21 (0.02)	0.23 (0.05)
71	isospathulenol	1635 ^a,b^	1633 ^a^	0.61 (0.01)	0.54 (0.04)	0.57 (0.02)	0.63 (0.04)	0.63 (0.04)	0.54 (0.03)
72	220[M+](18) 91(100) 105(83)	1639	ND	1.59 (0.02)	1.96 (0.05)	1.98 (0.03)	1.87 (0.05)	1.89 (0.04)	2.01 (0.02)
73	cubenol	1642 ^a,b,c,d^	1645 ^c^	0.53 (0.01)	0.47 (0.03)	0.54 (0.04)	0.55 (0.02)	0.51 (0.04)	0.55 (0.06)
74	220[M+](21) 91(100) 105(82)	1651	ND	0.10 (0.01)	0.07 (0.02)	0.11 (0.02)	0.12 (0.02)	0.10 (0.03)	0.09 (0.03)
75	222[M+](3) 179(100) 121(92)	1655	ND	0.05 (0.02)	0.03 (0.01)	0.01 (0.01)	0.06 (0.01)	0.05 (0.02)	0.03 (0.01)
76	germacra-4(15),5,10(14)-trien-1-α-ol	1660^c^	1685^c^	0.57 (0.04)	0.61 (0.05)	0.61(0.01)	0.59 (0.03)	0.62 (0.05)	0.65 (0.05)
77	216[M+](31) 145(100) 91(97)	1699	ND	0.41 (0.03)	0.43 (0.05)	0.38 (0.02)	0.40 (0.04)	0.47 (0.05)	0.44 (0.04)
78	1,4-dimethyl-7-(1-methylethyl)-azulene	1790 ^c^	1779 ^c^	2.75 (0.06)	2.68 (0.04)	2.77 (0.04)	2.75 (0.05)	2.69 (0.04)	2.71 (0.03)
79	14-hydroxy-δ-cadinene	1797 ^c^	1803 ^c^	0.39 (0.02)	0.27 (0.03)	0.29 (0.03)	0.31 (0.05)	0.33 (0.03)	0.27 (0.02)
	Total			96.30 (2.51)	96.65 (2.71)	96.22 (2.42)	96.11 (2.64)	96.39 (2.67)	96.42 (2.84)
	% identified			78.15 (1.59)	78.39 (1.65)	78.00 (1.33)	77.35 (1.51)	77.76 (1.56)	78.02 (1.72)
	including:								
	aliphatics			2.58 (0.20)	2.60 (0.29)	2.58 (0.20)	2.57 (0.22)	2.44 (0.28)	2.35 (0.28)
	aromatics			3.58 (0.63)	3.60 (0.26)	3.62 (0.14)	3.55 (0.18)	3.58 (0.19)	3.52 (0.21)
	monoterpene hydrocarbons			0.02 (0.01)	0.03 (0.01)	0.01 (0.01)	0.05 (0.01)	0.03 (0.01)	0.02 (0.01)
	monoterpenoid hydrocarbons			0.08 (0.01)	0.07 (0.01)	0.07 (0.02)	0.06 (0.02)	0.05 (0.01)	0.08 (0.03)
	sesquiterpene hydrocarbons			49.90 (0.57)	50.67 (0.76)	49.61 (0.70)	49.92 (0.77)	50.12 (0.71)	50.81 (0.78)
	sesquiterpenoid hydrocarbons			21.99 (0.19)	21.42 (0.32)	22.11 (0.26)	21.20 (0.31)	21.54 (0.36)	21.24 (0.41)
(**b**)									
**No.**	Compounds *	RI **	RI ***	Code ****					
				**CI-7**	**CI-8**	**CI-9**	**CI-10**	**CI-11**	**CI-12**
1	propan-1-ol	<700 ^a,b^	483 ^a^	1.18 (0.03)	1.12 (0.03)	1.03 (0.04)	1.08 (0.03)	1.19 (0.04)	0.95 (0.03)
2	pentanal	705 ^a,b,c,d^	704 ^c^	0.39 (0.04)	0.26 (0.04)	0.31 (0.03)	0.34 (0.04)	0.36 (0.02)	0.29 (0.03)
3	hexanal	802 ^a,b,c,d^	801 ^c^	0.21 (0.03)	0.16 (0.03)	0.19 (0.03)	0.21 (0.02)	0.20 (0.04)	0.17 (0.02)
4	hexan-1-ol	867 ^a,b^	869 ^a^	0.52 (0.04)	0.53 (0.02)	0.43 (0.02)	0.50 (0.04)	0.52 (0.03)	0.48 (0.03)
5	heptanal	902 ^a,b,c,d^	901 ^c^	0.10 (0.02)	0.03 (0.01)	0.07 (0.01)	0.02 (0.01)	0.04 (0.01)	0.05 (0.01)
6	α-pinene	939 ^a,b,c^	932 ^c^	0.05 (0.01)	0.01 (0.04)	0.03 (0.01)	0.04 (0.01)	0.02 (0.01)	0.07 (0.01)
7	benzaldehyde	940 ^a,b,c^	952 ^c^	0.15 (0.03)	0.23 (0.05)	0.19 (0.04)	0.26 (0.02)	0.16 (0.04)	0.24 (0.04)
8	2-ethylhexan-1-ol	1023 ^a,b^	1025 ^a^	0.07 (0.03)	0.14 (0.03)	0.13 (0.05)	0.07 (0.02)	0.06 (0.01)	0.04 (0.01)
9	phenylmethanol	1028 ^a,b,c^	1026 ^c^	1.07 (0.05)	1.15 (0.02)	1.21 (0.03)	1.04 (0.04)	1.03 (0.04)	1.11 (0.03)
10	phenylacetaldehyde	1044 ^a,b^	1044 ^a^	1.24 (0.04)	1.14 (0.05)	1.16 (0.03)	1.10 (0.04)	1.16 (0.04)	1.09 (0.03)
11	nonanal	1102 ^a,b,c,d^	1100 ^c^	0.09 (0.02)	0.07 (0.04)	0.06 (0.02)	0.15 (0.03)	0.13 (0.02)	0.11 (0.02)
12	3,4-dimethylcyclohexan-1-ol	1115 ^a,b^	1126 ^a^	0.08 (0.03)	0.11 (0.03)	0.13 (0.04)	0.14 (0.03)	0.11 (0.03)	0.09 (0.01)
13	phenylethanol	1121 ^a,b^	1121 ^a^	0.09 (0.03)	0.11 (0.05)	0.12 (0.05)	0.14 (0.03)	0.10 (0.04)	0.11 (0.04)
14	decanal	1195 ^a,b,c,d^	1201 ^c^	0.09 (0.04)	0.10 (0.02)	0.06 (0.04)	0.04 (0.01)	0.06 (0.02)	0.10 (0.04)
15	β-cyclocitral	1221 ^c^	1217 ^c^	0.04 (0.02)	0.09 (0.03)	0.08 (0.02)	0.11 (0.04)	0.07 (0.01)	0.06 (0.02)
16	2-phenoxyethan-1-ol	1225 ^a,b^	1226 ^a^	0.89 (0.04)	0.93 (0.02)	0.99 (0.06)	1.01 (0.05)	0.89 (0.05)	0.93 (0.03)
17	bicycloelemene	1316 ^a^	1330 ^a^	0.16 (0.02)	0.18 (0.05)	0.14 (0.02)	0.09 (0.02)	0.12 (0.04)	0.11 (0.02)
18	δ-elemene	1324 ^a,b,c^	1335 ^c^	0.85 (0.03)	0.79 (0.04)	0.75 (0.05)	0.71 (0.05)	0.69 (0.05)	0.64 (0.04)
19	204[M+](5) 121(100) 93(89)	1343	ND	0.15 (0.03)	0.16 (0.03)	0.23 (0.04)	0.25 (0.04)	0.16 (0.02)	0.13 (0.01)
20	200[M+](39) 159(100) 117(95)	1345	ND	1.12 (0.02)	1.16 (0.04)	0.97 (0.06)	1.02 (0.03)	1.05 (0.03)	0.96 (0.03)
21	202[M+](13) 81(100) 96(73)	1350	ND	0.09 (0.01)	0.15 (0.05)	0.16 (0.02)	0.17 (0.03)	0.14 (0.05)	0.09 (0.02)
22	204[M+](10) 119(100) 91(84)	1353	ND	0.06 (0.02)	0.09 (0.03)	0.07 (0.01)	0.04 (0.01)	0.03 (0.01)	0.08 (0.02)
23	anastreptene	1370 ^a^	1370 ^a^	18.03 (0.06)	17.92 (0.06)	17.87 (0.06)	18.23 (0.06)	18.14 (0.06)	18.06 (0.05)
24	204[M+](5) 81(100) 93(96)	1384	ND	0.23 (0.04)	0.17 (0.03)	0.25 (0.04)	0.27 (0.02)	0.17 (0.03)	0.20 (0.04)
25	β-elemene	1391 ^a,b,c^	1389 ^c^	2.67 (0.04)	2.87 (0.03)	2.75 (0.02)	2.84 (0.04)	2.80 (0.05)	2.77 (0.03)
26	204[M+](13) 157(100) 185(84)	1398	ND	0.18 (0.02)	0.29 (0.05)	0.35 (0.04)	0.31 (0.05)	0.37 (0.04)	0.21 (0.05)
27	204[M+](13) 157(100) 185(84)	1417	ND	0.20 (0.03)	0.31 (0.06)	0.30 (0.05)	0.33 (0.05)	0.37 (0.03)	0.28 (0.03)
28	204[M+](19) 135(100) 105(82)	1423	ND	0.19 (0.03)	0.21 (0.03)	0.27 (0.05)	0.16 (0.03)	0.21 (0.05)	0.23 (0.04)
29	204[M+](9) 91(100) 105(93)	1425	ND	0.07 (0.02)	0.02 (0.01)	0.04 (0.01)	0.06 (0.01)	0.07 (0.02)	0.02 (0.01)
30	(-)-aristolene	1429 ^a,b,c,d^	1428 ^a^	0.91 (0.04)	0.96 (0.05)	0.99 (0.03)	1.03 (0.05)	1.06 (0.05)	1.11 (0.04)
31	204[M+](9) 107(100) 79(43)	1432	ND	0.14 (0.03)	0.18 (0.05)	0.21 (0.04)	0.23 (0.04)	0.10 (0.04)	0.13 (0.03)
32	γ-maaliene	1435 ^a,b^	1427 ^a^	0.45 (0.05)	0.49 (0.04)	0.52 (0.03)	0.54 (0.02)	0.51 (0.04)	0.48 (0.05)
33	α-maaliene	1443 ^a,b^	1442 ^a^	0.30 (0.04)	0.28 (0.04)	0.26 (0.02)	0.36 (0.04)	0.31 (0.04)	0.36 (0.02)
34	aromandendrene	1445 ^a,b^	1447 ^a^	3.09 (0.03)	3.27 (0.05)	3.09 (0.04)	3.12 (0.04)	3.29 (0.05)	3.33 (0.04)
35	selina-5,11-diene	1447 ^a,b^	1454 ^a^	0.56 (0.05)	0.51 (0.02)	0.53 (0.04)	0.63 (0.05)	0.67 (0.05)	0.53 (0.05)
36	dehydroaromadendrene	1456 ^c^	1460 ^c^	1.15 (0.06)	1.09 (0.04)	1.22 (0.02)	1.21 (0.03)	1.19 (0.03)	1.11 (0.02)
37	1,2,9,10-tetradehydroaristolane	1461	ND	0.49 (0.04)	0.39 (0.06)	0.42 (0.04)	0.45 (0.04)	0.43 (0.03)	0.52 (0.04)
38	204[M+](15) 91(100) 105(84)	1465	ND	0.45 (0.03)	0.47 (0.05)	0.29 (0.03)	0.27 (0.03)	0.25 (0.04)	0.33 (0.03)
39	204[M+](18) 128(100) 143(95)	1469	ND	0.30 (0.05)	0.29 (0.04)	0.27 (0.03)	0.30 (0.05)	0.33 (0.03)	0.35 (0.03)
40	γ-gurjunene	1474 ^c,d^	1475 ^c^	0.46 (0.02)	0.39 (0.04)	0.55 (0.02)	0.49 (0.05)	0.45 (0.05)	0.53 (0.03)
41	γ-muurolene	1477 ^c^	1478 ^c^	0.13 (0.03)	0.11 (0.03)	0.09 (0.01)	0.20 (0.03)	0.18 (0.03)	0.19 (0.05)
42	δ-selinene	1488 ^c^	1492 ^c^	1.24 (0.03)	1.47 (0.04)	1.58 (0.02)	1.49 (0.05)	1.50 (0.02)	1.56 (0.04)
43	ledene	1492 ^a,b,c^	1496 ^c^	1.72 (0.03)	1.66 (0.04)	1.63 (0.04)	1.62 (0.06)	1.54 (0.05)	1.66 (0.03)
44	204[M+](38) 105(100) 93(96)	1495	ND	0.33 (0.04)	0.34 (0.03)	0.26 (0.03)	0.34 (0.02)	0.27 (0.03)	0.29 (0.05)
45	bicyclogermacrene	1499 ^a,b,c^	1500 ^c^	8.43 (0.05)	8.27 (0.06)	8.46 (0.06)	8.32 (0.04)	8.24 (0.06)	8.45 (0.06)
46	204[M+](19) 93(100) 91(95)	1505	ND	0.18 (0.03)	0.15 (0.03)	0.12 (0.02)	0.23 (0.05)	0.22 (0.03)	0.15 (0.03)
47	202[M+](25) 133(100) 91(89)	1509	ND	0.16 (0.05)	0.17 (0.03)	0.24 (0.03)	0.25 (0.03)	0.14 (0.04)	0.27 (0.03)
48	206[M+](14) 191(100) 57(38)	1514	ND	0.22 (0.04)	0.24 (0.04)	0.19 (0.05)	0.17 (0.02)	0.26 (0.03)	0.20 (0.02)
49	202[M+](33) 131(100) 145(53)	1518	ND	0.19 (0.04)	0.22 (0.04)	0.23 (0.02)	0.21 (0.04)	0.22 (0.05)	0.18 (0.04)
50	δ-cadinene	1524 ^a,b,c^	1522 ^c^	0.35 (0.03)	0.37 (0.03)	0.32 (0.03)	0.24 (0.03)	0.26 (0.03)	0.35 (0.05)
51	204[M+](5) 91(100) 131(95)	1530	ND	0.09 (0.04)	0.07 (0.02)	0.06 (0.04)	0.12 (0.04)	0.13 (0.02)	0.15 (0.03)
52	200[M+](54) 185(100) 143(91)	1535	ND	0.12 (0.03)	0.15 (0.05)	0.21 (0.05)	0.14 (0.05)	0.15 (0.05)	0.19 (0.05)
53	4,5,9,10-dehydro-isolongifolene	1544 ^a,b^	1544 ^a^	6.29 (0.07)	6.34 (0.07)	6.21 (0.06)	6.40 (0.06)	6.37 (0.06)	6.39 (0.06)
54	202[M+](4) 128(100) 157(95)	1547	ND	0.67 (0.06)	0.61 (0.05)	0.64 (0.04)	0.72 (0.04)	0.75 (0.05)	0.64 (0.04)
55	200[M+](8) 171(100) 186(79)	1551	ND	0.17 (0.04)	0.18 (0.03)	0.20 (0.03)	0.16 (0.03)	0.17 (0.03)	0.19 (0.03)
56	200[M+](91) 129(100) 157(88)	1556	ND	0.14 (0.03)	0.13 (0.04)	0.08 (0.02)	0.06 (0.02)	0.04 (0.01)	0.07 (0.02)
57	204[M+](8) 143(100) 157(98)	1559	ND	0.12 (0.03)	0.06 (0.01)	0.07 (0.02)	0.09 (0.02)	0.10 (0.05)	0.12 (0.04)
58	204[M+](82) 173(100) 189(94)	1563	ND	1.33 (0.04)	1.37 (0.05)	1.47 (0.04)	1.40 (0.04)	1.48 (0.03)	1.51 (0.04)
59	palustrol	1567 ^c^	1567 ^c^	8.26 (0.03)	8.46 (0.06)	8.41 (0.06)	8.38 (0.06)	8.29 (0.06)	8.27 (0.06)
60	200[M+](11) 79(100) 93(95)	1570	ND	0.85 (0.05)	0.76 (0.04)	0.77 (0.03)	0.48 (0.03)	0.55 (0.04)	0.90 (0.05)
61	204[M+](31) 81(100) 109(88)	1573	ND	2.08 (0.04)	2.11 (0.04)	1.98 (0.04)	1.96 (0.05)	2.04 (0.05)	2.11 (0.02)
62	spathulenol	1576 ^a,b,c^	1577 ^c^	7.65 (0.05)	7.81 (0.06)	7.78 (0.07)	7.69 (0.06)	7.21 (0.03)	7.54 (0.05)
63	200[M+](56) 185(100) 143(63)	1581	ND	3.67 (0.02)	3.50 (0.06)	3.71 (0.04)	3.60 (0.05)	3.54 (0.05)	3.68 (0.05)
64	202[M+](4) 91(100) 79(82)	1587	ND	0.56 (0.04)	0.41 (0.03)	0.43 (0.03)	0.52 (0.04)	0.49 (0.05)	0.47 (0.02)
65	globulol	1599 ^a,b,c,d^	1590 ^c^	3.04 (0.05)	3.11 (0.02)	2.87 (0.04)	2.93 (0.04)	2.94 (0.04)	3.13 (0.05)
66	200[M+](8) 198(100) 183(84)	1605	ND	0.27 (0.03)	0.30 (0.05)	0.19 (0.03)	0.17 (0.03)	0.24 (0.03)	0.29 (0.03)
67	220[M+](2) 145(100) 200(93)	1609	ND	1.18 (0.02)	1.14 (0.04)	1.26 (0.04)	1.31 (0.02)	1.18 (0.03)	1.23 (0.05)
68	(+)-bisabola-2,10-diene [1,9]oxide	1615 ^a,b^	1596 ^a^	0.21 (0.04)	0.22 (0.03)	0.12 (0.03)	0.15 (0.03)	0.18 (0.03)	0.20 (0.02)
69	208[M+](3) 95(100) 85(95)	1621	ND	0.80 (0.03)	0.79 (0.05)	0.71 (0.02)	0.66 (0.05)	0.73 (0.05)	0.74 (0.40)
70	ledene oxide-(II)	1631 ^a,b^	1631 ^a^	0.24 (0.02)	0.19 (0.03)	0.20 (0.03)	0.23 (0.04)	0.24 (0.03)	0.29 (0.03)
71	isospathulenol	1635 ^a,b^	1633 ^a^	0.59 (0.04)	0.51 (0.05)	0.49 (0.04)	0.53 (0.05)	0.64 (0.05)	0.59 (0.04)
72	220[M+](18) 91(100) 105(83)	1639	ND	1.91 (0.02)	1.67 (0.05)	1.74 (0.05)	1.86 (0.03)	1.99 (0.03)	2.01 (0.05)
73	cubenol	1642 ^a,b,c,d^	1645 ^c^	0.47 (0.04)	0.39 (0.03)	0.41 (0.03)	0.44 (0.05)	0.49 (0.03)	0.40 (0.04)
74	220[M+](21) 91(100) 105(82)	1651	ND	0.07 (0.03)	0.06 (0.02)	0.12 (0.02)	0.10 (0.03)	0.11 (0.02)	0.09 (0.02)
75	222[M+](3) 179(100) 121(92)	1655	ND	0.07 (0.01)	0.02 (0.01)	0.01 (0.01)	0.02 (0.01)	0.01 (0.01)	0.04 (0.01)
76	germacra-4(15),5,10(14)-trien-1-α-ol	1660 ^c^	1685 ^c^	0.47 (0.02)	0.51 (0.05)	0.60 (0.03)	0.57 (0.05)	0.63 (0.03)	0.59 (0.04)
77	216[M+](31) 145(100) 91(97)	1699	ND	0.39 (0.06)	0.45 (0.04)	0.51 (0.04)	0.43 (0.03)	0.46 (0.05)	0.39 (0.04)
78	1,4-dimethyl-7-(1-methylethyl)-azulene	1790 ^c^	1779 ^c^	2.74 (0.02)	2.62 (0.06)	2.71 (0.03)	2.59 (0.05)	2.63 (0.02)	2.75 (0.04)
79	14-hydroxy-δ-cadinene	1797 ^c^	1803 ^c^	0.25 (0.01)	0.34 (0.03)	0.40 (0.02)	0.42 (0.02)	0.39 (0.05)	0.39 (0.05)
	Total			96.21 (2.69)	96.10 (3.04)	96.17 (2.67)	96.56 (2.83)	96.01 (2.83)	97.11 (3.04)
	% identified			77.46 (1.54)	77.70 (1.72)	77.56 (1.51)	78.15 (1.67)	77.49 (1.61)	78.19 (1.54)
	including:								
	aliphatics			2.73 (0.28)	2.52 (0.25)	2.41 (0.28)	2.55 (0.23)	2.67 (0.22)	2.28 (0.20)
	aromatics			3.44 (0.19)	3.56 (0.19)	3.67 (0.21)	3.55 (0.18)	3.34 (0.21)	3.48 (0.17)
	monoterpene hydrocarbons			0.05 (0.01)	0.01 (0.04)	0.03 (0.01)	0.04 (0.01)	0.02 (0.01)	0.07 (0.01)
	monoterpenoid hydrocarbons			0.04 (0.02)	0.09 (0.03)	0.08 (0.02)	0.11 (0.04)	0.07 (0.01)	0.06 (0.02)
	sesquiterpene hydrocarbons			50.02 (0.74)	49.98 (0.85)	50.09 (064)	50.56 (0.81)	50.38 (0.81)	50.90 (0.76)
	sesquiterpenoid hydrocarbons			21.18 (0.30)	21.54 (0.36)	21.28 (0.35)	21.34 (0.40)	21.01 (0.35)	21.40 (0.38)

- less than 0.01%. * The names of terpenes and terpenoids according to IUPAC terminology are given in Table S5. ** Retention index on Quadex 007-5MS column. *** Literature retention index. ND, no data. **** For abbreviations of samples, see Table 1. Standard deviation in brackets. Identification of compounds by MS databases (^a^ NIST 2011, ^b^ NIST Chemistry WebBook, ^c^ Adams 4 Library, ^d^ Pherobase).

**Table 3:** (**a**) Volatile compounds detected in the samples collected in summer (CI-13–CI-18). (**b**) Volatile compounds detected in the samples collected in summer (CI-19–CI-24).

(a)									
No.	Compounds *	RI **	RI ***	Code ****					
				CI-13	CI-14	CI-15	CI-16	CI-17	CI-18
1	propan-1-ol	<700 ^a,b^	483 ^a^	0.43 (0.03)	0.39 (0.03)	0.35 (0.04)	0.48 (0.04)	0.31 (0.04)	0.29 (0.05)
2	pentanal	705 ^a,b,c,d^	704 ^c^	0.32 (0.04)	0.28 (0.05)	0.29 (0.02)	0.33 (0.02)	0.35 (0.02)	0.29 (0.01)
3	hexanal	802 ^a,b,c,d^	801 ^c^	0.08 (0.02)	0.07 (0.01)	0.05 (0.01)	0.09 (0.01)	0.06 (0.01)	0.05 (0.03)
4	hexan-1-ol	867 ^a,b^	869 ^a^	0.33 (0.04)	0.33 (0.03)	0.29 (0.03)	0.31 (0.03)	0.32 (0.03)	0.33 (0.01)
5	heptanal	902 ^a,b,c,d^	901 ^c^	0.05 (0.03)	0.04 (0.01)	0.05 (0.01)	0.04 (0.01)	0.03 (0.01)	0.05 (0.01)
6	α-pinene	939 ^a,b,c^	932 ^c^	0.02 (0.01)	0.02 (0.01)	0.01 (0.01)	0.02 (0.01)	0.02 (0.01)	0.03 (0.01)
7	benzaldehyde	940 ^a,b,c^	952 ^c^	0.10 (0.04)	0.07 (0.01)	0.06 (0.02)	0.09 (0.02)	0.12 (0.03)	0.08 (0.01)
8	2-ethylhexan-1-ol	1023 ^a,b^	1025 ^a^	0.04 (0.02)	0.04 (0.01)	0.03 (0.01)	0.02 (0.01)	0.04 (0.03)	0.05 (0.01)
9	phenylmethanol	1028 ^a,b,c^	1026 ^c^	1.06 (0.04)	1.08 (0.03)	0.98 (0.05)	1.01 (0.04)	1.03 (0.01)	1.09 (0.02)
10	phenylacetaldehyde	1044 ^a,b^	1044 ^a^	0.21 (0.04)	0.22 (0.03)	0.20 (0.04)	0.18 (0.04)	0.19 (0.03)	0.21 (0.04)
11	nonanal	1102 ^a,b,c,d^	1100 ^c^	0.02 (0.01)	0.02 (0.01)	0.01 (0.01)	0.01 (0.00)	0.02 (0.01)	0.03 (0.01)
12	3,4-dimethylcyclohexan-1-ol	1115 ^a,b^	1126 ^a^	0.05 (0.01)	0.01 (0.01)	0.03 (0.01)	0.02 (0.01)	0.01 (0.01)	0.02 (0.01)
13	phenylethanol	1121 ^a,b^	1121 ^a^	0.69 (0.05)	0.67 (0.04)	0.71 (0.05)	0.68 (0.05)	0.69 (0.04)	0.65 (0.05)
14	decanal	1195 ^a,b,c,d^	1201^c^	0.02 (0.01)	0.03 (0.01)	0.01 (0.00)	0.02 (0.01)	0.03 (0.01)	0.04 (0.01)
15	β-cyclocitral	1221 ^c^	1217^c^	0.04 (0.01)	0.05 (0.01)	0.04 (0.01)	0.03 (0.01)	0.05 (0.01)	0.03 (0.01)
16	2-phenoxyethan-1-ol	1225 ^a,b^	1226 ^a^	0.41 (0.05)	0.38 (0.04)	0.45 (0.05)	0.41 (0.05)	0.42 (0.04)	0.43 (0.03)
17	bicycloelemene	1316 ^a^	1330 ^a^	0.08 (0.01)	0.09 (0.02)	0.09 (0.03)	0.07 (0.03)	0.08 (0.01)	0.06 (0.01)
18	δ-elemene	1324 ^a,b,c^	1335 ^c^	1.31 (0.05)	2.26 (0.04)	1.89 (0.03)	1.89 (0.04)	1.97 (0.06)	2.01 (0.06)
19	204[M+](5) 121(100) 93(89)	1343	ND	0.37 (0.04)	0.45 (0.04)	0.36 (0.02)	0.33 (0.03)	0.51 (0.02)	0.47 (0.02)
20	200[M+](39) 159(100) 117(95)	1345	ND	0.88 (0.05)	0.49 (0.03)	0.64 (0.03)	0.55 (0.04)	0.91 (0.04)	0.67 (0.04)
21	202[M+](13) 81(100) 96(73)	1350	ND	0.16 (0.02)	0.21 (0.05)	0.18 (0.04)	0.20 (0.05)	0.22 (0.03)	0.23 (0.03)
22	204[M+](10) 119(100) 91(84)	1353	ND	0.07 (0.01)	0.06 (0.02)	0.05 (0.01)	0.08 (0.02)	0.06 (0.01)	0.04 (0.03)
23	anastreptene	1370 ^a^	1370 ^a^	25.33 (0.05)	25.26 (0.06)	25.21 (0.06)	25.33 (0.06)	25.06 (0.05)	25.12 (0.06)
24	204[M+](5) 81(100) 93(96)	1384	ND	0.16 (0.05)	0.12 (0.04)	0.12 (0.02)	0.13 (0.02)	0.15 (0.02)	0.17 (0.04)
25	β-elemene	1391 ^a,b,c^	1389 ^c^	2.58 (0.03)	1.85 (0.02)	2.01 (0.04)	1.98 (0.04)	2.38 (0.04)	2.47 (0.05)
26	204[M+](13) 157(100) 185(84)	1398	ND	0.27 (0.02)	0.18 (0.04)	0.19 (0.03)	0.20 (0.03)	0.22 (0.03)	0.25 (0.02)
27	204[M+](13) 157(100) 185(84)	1417	ND	0.21 (0.06)	0.28 (0.03)	0.21 (0.03)	0.23 (0.03)	0.29 (0.03)	0.27 (0.06)
28	204[M+](19) 135(100) 105(82)	1423	ND	0.23 (0.02)	0.35 (0.04)	0.37 (0.02)	0.25 (0.05)	0.26 (0.05)	0.27 (0.02)
29	204[M+](9) 91(100) 105(93)	1425	ND	0.08 (0.02)	0.08 (0.01)	0.07 (0.02)	0.06 (0.02)	0.09 (0.02)	0.11 (0.04)
30	(-)-aristolene	1429 ^a,b,c,d^	1428 ^a^	0.75 (0.04)	0.71 (0.04)	0.77 (0.05)	0.76 (0.05)	0.69 (0.04)	0.67 (0.05)
31	204[M+](9) 107(100) 79(43)	1432	ND	0.19 (0.04)	0.26 (0.03)	0.20 (0.03)	0.21 (0.04)	0.25 (0.03)	0.17 (0.03)
32	γ-maaliene	1435 ^a,b^	1427 ^a^	0.40 (0.03)	0.59 (0.04)	0.41 (0.04)	0.39 (0.04)	0.37 (0.04)	0.47 (0.04)
33	α-maaliene	1443 ^a,b^	1442 ^a^	0.29 (0.02)	0.53 (0.03)	0.35 (0.04)	0.47 (0.04)	0.44 (0.05)	0.51 (0.04)
34	aromandendrene	1445 ^a,b^	1447 ^a^	6.23 (0.05)	7.87 (0.04)	5.17 (0.04)	7.45 (0.03)	7.54 (0.06)	6.96 (0.04)
35	selina-5,11-diene	1447 ^a,b^	1454 ^a^	0.54 (0.05)	0.87 (0.03)	0.64 (0.03)	0.77 (0.02)	0.81 (0.03)	0.79 (0.04)
36	dehydroaromadendrene	1456 ^c^	1460 ^c^	1.44 (0.02)	1.55 (0.02)	1.47 (0.02)	1.51 (0.04)	1.38 (0.02)	1.56 (0.03)
37	1,2,9,10-tetradehydroaristolane	1461	ND	1.31 (0.04)	0.92 (0.04)	1.21 (0.04)	1.08 (0.05)	1.11 (0.04)	1.23 (0.03)
38	204[M+](15) 91(100) 105(84)	1465	ND	0.32 (0.03)	0.39 (0.02)	0.30 (0.04)	0.29 (0.05)	0.41 (0.04)	0.35 (0.04)
39	204[M+](18) 128(100) 143(95)	1469	ND	0.41 (0.03)	0.25 (0.04)	0.39 (0.04)	0.38 (0.04)	0.34 (0.02)	0.44 (0.02)
40	γ-gurjunene	1474 ^c,d^	1475 ^c^	0.43 (0.04)	0.46 (0.02)	0.40 (0.02)	0.45 (0.03)	0.39 (0.05)	0.47 (0.04)
41	γ-muurolene	1477 ^c^	1478 ^c^	0.22 (0.06)	0.24 (0.05)	0.18 (0.04)	0.16 (0.03)	0.27 (0.04)	0.21 (0.04)
42	δ-selinene	1488 ^c^	1492 ^c^	2.14 (0.05)	2.21 (0.04)	2.01 (0.03)	2.25 (0.04)	2.19 (0.03)	2.20 (0.03)
43	ledene	1492 ^a,b,c^	1496 ^c^	1.97 (0.03)	2.08 (0.03)	2.00 (0.04)	2.01 (0.03)	2.10 (0.02)	2.03 (0.02)
44	204[M+](38) 105(100) 93(96)	1495	ND	0.23 (0.02)	0.10 (0.02)	0.25 (0.04)	0.18 (0.03)	0.15 (0.02)	0.27 (0.05)
45	bicyclogermacrene	1499 ^a,b,c^	1500 ^c^	17.62 (0.03)	18.09 (0.05)	17.72 (0.06)	16.78 (0.05)	16.89 (0.01)	16.45 (0.05)
46	204[M+](19) 93(100) 91(95)	1505	ND	0.20 (0.04)	0.64 (0.06)	0.41 (0.02)	0.23 (0.04)	0.57 (0.03)	0.44 (0.03)
47	202[M+](25) 133(100) 91(89)	1509	ND	0.07 (0.03)	0.14 (0.05)	0.08 (0.01)	0.10 (0.03)	0.12 (0.03)	0.18 (0.03)
48	206[M+](14) 191(100) 57(38)	1514	ND	0.10 (0.02)	0.29 (0.03)	0.12 (0.03)	0.15 (0.03)	0.33 (0.04)	0.18 (0.04)
49	202[M+](33) 131(100) 145(53)	1518	ND	0.42 (0.04)	0.38 (0.05)	0.41 (0.03)	0.37 (0.04)	0.35 (0.03)	0.47 (0.04)
50	δ-cadinene	1524 ^a,b,c^	1522 ^c^	0.09 (0.01)	0.14 (0.03)	0.10 (0.04)	0.15 (0.04)	0.12 (0.04)	0.08 (0.02)
51	204[M+](5) 91(100) 131(95)	1530	ND	0.19 (0.04)	0.11 (0.02)	0.16 (0.04)	0.20 (0.02)	0.21 (0.04)	0.14 (0.03)
52	200[M+](54) 185(100) 143(91)	1535	ND	-	-	-	-	-	-
53	4,5,9,10-dehydro-isolongifolene	1544 ^a,b^	1544 ^a^	4.89 (0.04)	4.14 (0.03)	4.79 (0.03)	4.38 (0.04)	4.56 (0.02)	4.78 (0.03)
54	202[M+](4) 128(100) 157(95)	1547	ND	0.81 (0.03)	0.69 (0.03)	0.79 (0.02)	0.71 (0.03)	0.65 (0.03)	0.83 (0.02)
55	200[M+](8) 171(100) 186(79)	1551	ND	0.07 (0.02)	0.10 (0.02)	0.06 (0.03)	0.09 (0.02)	0.11 (0.04)	0.06 (0.01)
56	200[M+](91) 129(100) 157(88)	1556	ND	0.08 (0.02)	0.05 (0.01)	0.07 (0.01)	0.08 (0.02)	0.06 (0.01)	0.04 (0.01)
57	204[M+](8) 143(100) 157(98)	1559	ND	0.06 (0.02)	0.04 (0.01)	0.02 (0.04)	0.03 (0.02)	0.05 (0.01)	0.07 (0.01)
58	204[M+](82) 173(100) 189(94)	1563	ND	1.23 (0.05)	1.21 (0.04)	1.18 (0.04)	1.22 (0.05)	1.24 (0.03)	1.19 (0.04)
59	palustrol	1567 ^c^	1567 ^c^	5.11 (0.02)	5.58 (0.04)	5.48 (0.03)	5.34 (0.04)	5.37 (0.03)	5.27 (0.06)
60	200[M+](11) 79(100) 93(95)	1570	ND	0.64 (0.04)	0.37 (0.03)	0.55 (0.03)	0.47 (0.04)	0.59 (0.04)	0.62 (0.03)
61	204[M+](31) 81(100) 109(88)	1573	ND	2.36 (0.02)	1.43 (0.03)	2.11 (0.04)	2.21 (0.02)	1.89 (0.04)	1.89 (0.03)
62	spathulenol	1576 ^a,b,c^	1577 ^c^	2.59 (0.05)	2.75 (0.04)	2.63 (0.04)	2.71 (0.03)	2.65 (0.06)	2.57 (0.04)
63	200[M+](56) 185(100) 143(63)	1581	ND	2.39 (0.05)	2.60 (0.06)	2.47 (0.03)	2.41 (0.03)	2.46 (0.03)	2.53 (0.03)
64	202[M+](4) 91(100) 79(82)	1587	ND	0.28 (0.02)	0.35 (0.03)	0.33 (0.02)	0.29 (0.04)	0.31 (0.03)	0.34 (0.02)
65	globulol	1599 ^a,b,c,d^	1590 ^c^	1.52 (0.04)	1.07 (0.03)	1.43 (0.04)	1.37 (0.06)	1.37 (0.04)	1.29 (0.03)
66	200[M+](8) 198(100) 183(84)	1605	ND	-	-	-	-	-	-
67	220[M+](2) 145(100) 200(93)	1609	ND	0.09 (0.01)	0.09 (0.02)	0.09 (0.04)	0.08 (0.02)	0.10 (0.04)	0.07 (0.01)
68	(+)-bisabola-2,10-diene[1,9]oxide	1615 ^a,b^	1596 ^a^	0.30 (0.04)	0.29 (0.04)	0.33 (0.02)	0.27 (0.02)	0.28 (0.03)	0.31 (0.04)
69	208[M+](3) 95(100) 85(95)	1621	ND	0.72 (0.03)	0.11 (0.03)	0.68 (0.03)	0.57 (0.03)	0.49 (0.02)	0.57 (0.04)
70	ledene oxide-(II)	1631 ^a,b^	1631 ^a^	-	-	-	-	-	-
71	isospathulenol	1635 ^a,b^	1633 ^a^	0.33 (0.05)	0.37 (0.04)	0.36 (0.05)	0.30 (0.04)	0.41 (0.02)	0.35 (0.03)
72	220[M+](18) 91(100) 105(83)	1639	ND	0.77 (0.03)	0.75 (0.05)	0.77 (0.03)	0.73 (0.05)	0.70 (0.04)	0.69 (0.03)
73	cubenol	1642 ^a,b,c,d^	1645 ^c^	0.10 (0.02)	0.05 (0.01)	0.07 (0.02)	0.09 (0.01)	0.05 (0.01)	0.04 (0.01)
74	220[M+](21) 91(100) 105(82)	1651	ND	0.05 (0.02)	0.03 (0.01)	0.06 (0.01)	0.04 (0.01)	0.03 (0.01)	0.02 (0.01)
75	222[M+](3) 179(100) 121(92)	1655	ND	0.05 (0.02)	0.05 (0.01)	0.05 (0.01)	0.04 (0.01)	0.03 (0.01)	0.06 (0.02)
76	germacra-4(15),5,10(14)-trien-1-α-ol	1660 ^c^	1685 ^c^	0.88 (0.05)	0.90 (0.05)	0.89 (0.05)	0.91 (0.04)	0.85 (0.05)	0.87 (0.03)
77	216[M+](31) 145(100) 91(97)	1699	ND	0.15 (0.05)	0.23 (0.06)	0.16 (0.05)	0.19 (0.03)	0.25 (0.03)	0.20 (0.04)
78	1,4-dimethyl-7-(1-methylethyl)-azulene	1790 ^c^	1779 ^c^	1.06 (0.06)	1.33 (0.05)	1.21 (0.03)	1.23 (0.02)	1.27 (0.04)	1.30 (0.05)
79	14-hydroxy-δ-cadinene	1797 ^c^	1803 ^c^	0.19 (0.03)	0.02 (0.01)	0.18 (0.04)	0.15 (0.02)	0.10 (0.02)	0.12 (0.01)
	Total			97.88 (2.47)	98.80 (2.34)	96.46 (2.30)	97.29 (2.37)	98.79 (2.23)	98.16 (2.26)
	% identified			83.57 (1.46)	85.92 (1.28)	82.56 (1.37)	83.99 (1.34)	84.39 (1.29)	83.86 (1.30)
	including:								
	aliphatics			1.34 (0.21)	1.21 (0.17)	1.11 (0.14)	1.32 (0.14)	1.17 (0.17)	1.15 (0.15)
	aromatics			2.47 (0.22)	2.42 (0.15)	2.40 (0.21)	2.37 (0.20)	2.45 (0.15)	2.46 (0.15)
	monoterpene hydrocarbons			0.02 (0.01)	0.02 (0.01)	0.01 (0.01)	0.02 (0.01)	0.02 (0.01)	0.03 (0.01)
	monoterpenoid hydrocarbons			0.04 (0.01)	0.05 (0.01)	0.04 (0.01)	0.03 (0.01)	0.05 (0.01)	0.03 (0.01)
	sesquiterpene hydrocarbons			68.68 (0.71)	71.19 (0.68)	67.63 (0.71)	69.11 (0.72)	69.62 (0.69)	69.37 (0.73)
	sesquiterpenoid hydrocarbons			11.02 (0.30)	11.03 (0.26)	11.37 (0.29)	11.14 (0.26)	11.08 (0.26)	10.82 (0.25)
(**b**)									
**No.**	Compounds *	RI **	RI ***	Code ****					
				**CI-19**	**CI-20**	**CI-21**	**CI-22**	**CI-23**	**CI-24**
1	propan-1-ol	<700 ^a,b^	483 ^a^	0.44 (0.03)	0.46 (0.03)	0.36 (0.03)	0.38 (0.03)	0.42 (0.03)	0.44 (0.02)
2	pentanal	705 ^a,b,c,d^	704 ^c^	0.27 (0.01)	0.26 (0.01)	0.31 (0.05)	0.34 (0.01)	0.33 (0.04)	0.30 (0.04)
3	hexanal	802 ^a,b,c,d^	801 ^c^	0.04 (0.01)	0.06 (0.01)	0.07 (0.01)	0.09 (0.01)	0.08 (0.01)	0.09 (0.02)
4	hexan-1-ol	867 ^a,b^	869 ^a^	0.35 (0.04)	0.29 (0.04)	0.31 (0.03)	0.32 (0.02)	0.34 (0.04)	0.35 (0.03)
5	heptanal	902 ^a,b,c,d^	901 ^c^	0.04 (0.01)	0.06 (0.01)	0.03 (0.01)	0.04 (0.01)	0.05 (0.01)	0.04 (0.01)
6	α-pinene	939 ^a,b,c^	932 ^c^	0.01 (0.01)	0.01 (0.01)	0. 01 (0.01)	0.02 (0.04)	0.03 (0.01)	0.04 (0.01)
7	benzaldehyde	940 ^a,b,c^	952 ^c^	0.06 (0.01)	0.08 (0.04)	0.11 (0.01)	0.12 (0.04)	0.10 (0.02)	0.09 (0.02)
8	2-ethylhexan-1-ol	1023 ^a,b^	1025 ^a^	0.03 (0.01)	0.02 (0.01)	0.03 (0.01)	0.04 (0.01)	0.04 (0.01)	0.05 (0.01)
9	phenylmethanol	1028 ^a,b,c^	1026 ^c^	1.11 (0.03)	0.96 (0.04)	0.96 (0.03)	1.02 (0.03)	1.01 (0.04)	1.07 (0.03)
10	phenylacetaldehyde	1044 ^a,b^	1044 ^a^	0.23 (0.04)	0.25 (0.03)	0.19 (0.03)	0.18 (0.01)	0.20 (0.03)	0.23 (0.03)
11	nonanal	1102 ^a,b,c,d^	1100 ^c^	0.02 (0.01)	0.01 (0.01)	0.02 (0.01)	0.03 (0.01)	0.02 (0.01)	0.01 (0.01)
12	3,4-dimethylcyclohexan-1-ol	1115 ^a,b^	1126 ^a^	0.03 (0.01)	0.05 (0.01)	0.04 (0.01)	0.06 (0.03)	0.03 (0.01)	0.04 (0.01)
13	phenylethanol	1121 ^a,b^	1121 ^a^	0.64 (0.03)	0.63 (0.02)	0.71 (0.02)	0.72 (0.03)	0.75 (0.02)	0.74 (0.04)
14	decanal	1195 ^a,b,c,d^	1201 ^c^	0.05 (0.01)	0.06 (0.01)	0.03 (0.01)	0.02 (0.01)	0.01 (0.01)	0.02 (0.01)
15	β-cyclocitral	1221 ^c^	1217 ^c^	0.02 (0.01)	0.04 (0.01)	0.05 (0.01)	0.03 (0.03)	0.02 (0.01)	0.06 (0.01)
16	2-phenoxyethan-1-ol	1225 ^a,b^	1226 ^a^	0.37 (0.04)	0.38 (0.04)	0.39 (0.04)	0.40 (0.01)	0.43 (0.04)	0.42 (0.02)
17	bicycloelemene	1316 ^a^	1330 ^a^	0.09 (0.01)	0.10 (0.01)	0.06 (0.01)	0.07 (0.01)	0.08 (0.02)	0.09 (0.01)
18	δ-elemene	1324 ^a,b,c^	1335 ^c^	2.13 (0.05)	2.22 (0.03)	2.18 (0.05)	2.09 (0.01)	2.04 (0.03)	1.76 (0.05)
19	204[M+](5) 121(100) 93(89)	1343	ND	0.42 (0.04)	0.44 (0.03)	0.46 (0.01)	0.39 (0.03)	0.41 (0.02)	0.45 (0.03)
20	200[M+](39) 159(100) 117(95)	1345	ND	0.74 (0.02)	0.81 (0.01)	0.80 (0.01)	0.71 (0.01)	0.73 (0.04)	0.75 (0.03)
21	202[M+](13) 81(100) 96(73)	1350	ND	0.19 (0.04)	0.17 (0.05)	0.20 (0.03)	0.21 (0.06)	0.24 (0.05)	0.18 (0.04)
22	204[M+](10) 119(100) 91(84)	1353	ND	0.08 (0.02)	0.09 (0.02)	0.10 (0.01)	0.08 (0.02)	0.06 (0.01)	0.05 (0.04)
23	anastreptene	1370 ^a^	1370 ^a^	25.41 (0.06)	25.32 (0.06)	25.29 (0.06)	25.30 (0.04)	25.24 (0.04)	25.26 (0.02)
24	204[M+](5) 81(100) 93(96)	1384	ND	0.13 (0.02)	0.14 (0.02)	0.15 (0.02)	0.18 (0.03)	0.20 (0.03)	0.21 (0.03)
25	β-elemene	1391 ^a,b,c^	1389 ^c^	2.46 (0.04)	2.38 (0.04)	2.21 (0.04)	2.18 (0.03)	1.99 (0.03)	2.01 (0.04)
26	204[M+](13) 157(100) 185(84)	1398	ND	0.24 (0.03)	0.19 (0.05)	0.20 (0.03)	0.22 (0.06)	0.24 (0.04)	0.22 (0.03)
27	204[M+](13) 157(100) 185(84)	1417	ND	0.25 (0.03)	0.27 (0.03)	0.26 (0.03)	0.24 (0.02)	0.26 (0.03)	0.27 (0.02)
28	204[M+](19) 135(100) 105(82)	1423	ND	0.33 (0.05)	0.36 (0.01)	0.31 (0.06)	0.30 (0.04)	0.29 (0.03)	0.27 (0.01)
29	204[M+](9) 91(100) 105(93)	1425	ND	0.12 (0.02)	0.08 (0.02)	0.09 (0.02)	0.07 (0.01)	0.06 (0.01)	0.10 (0.01)
30	(-)-aristolene	1429 ^a,b,c,d^	1428 ^a^	0.65 (0.04)	0.63 (0.01)	0.76 (0.04)	0.77 (0.05)	0.80 (0.02)	0.73 (0.01)
31	204[M+](9) 107(100) 79(43)	1432	ND	0.21 (0.03)	0.26 (0.05)	0.27 (0.03)	0.30 (0.02)	0.31 (0.04)	0.27 (0.04)
32	γ-maaliene	1435 ^a,b^	1427 ^a^	0.52 (0.06)	0.55 (0.04)	0.57 (0.03)	0.61 (0.06)	0.54 (0.03)	0.56 (0.06)
33	α-maaliene	1443 ^a,b^	1442 ^a^	0.35 (0.03)	0.34 (0.02)	0.33 (0.05)	0.34 (0.02)	0.47 (0.03)	0.49 (0.03)
34	aromandendrene	1445 ^a,b^	1447 ^a^	6.87 (0.02)	6.78 (0.04)	7.21 (0.03)	7.44 (0.04)	6.45 (0.05)	7.54 (0.03)
35	selina-5,11-diene	1447 ^a,b^	1454 ^a^	0.63 (0.04)	0.67 (0.02)	0.72 (0.04)	0.75 (0.05)	0.81 (0.02)	0.76 (0.04)
36	dehydroaromadendrene	1456^c^	1460 ^c^	1.49 (0.04)	1.39 (0.06)	1.40 (0.03)	1.61 (0.03)	1.57 (0.04)	1.52 (0.03)
37	1,2,9,10-tetradehydroaristolane	1461	ND	1.29 (0.02)	1.04 (0.03)	1.00 (0.04)	0.98 (0.01)	1.24 (0.03)	1.27 (0.02)
38	204[M+](15) 91(100) 105(84)	1465	ND	0.27 (0.05)	0.43 (0.04)	0.45 (0.05)	0.30 (0.02)	0.36 (0.02)	0.42 (0.03)
39	204[M+](18) 128(100) 143(95)	1469	ND	0.33 (0.03)	0.37 (0.02)	0.38 (0.06)	0.42 (0.01)	0.33 (0.03)	0.43 (0.02)
40	γ-gurjunene	1474 ^c,d^	1475 ^c^	0.49 (0.04)	0.52 (0.04)	0.39 (0.03)	0.41 (0.05)	0.51 (0.02)	0.55 (0.06)
41	γ-muurolene	1477^c^	1478 ^c^	0.22 (0.03)	0.19 (0.04)	0.20 (0.05)	0.23 (0.04)	0.26 (0.04)	0.27 (0.02)
42	δ-selinene	1488 ^c^	1492 ^c^	2.17 (0.04)	2.10 (0.03)	2.09 (0.02)	2.22 (0.02)	2.25 (0.04)	2.16 (0.04)
43	ledene	1492 ^a,b,c^	1496 ^c^	2.11 (0.03)	1.87 (0.02)	1.92 (0.04)	1.95 (0.02)	1.90 (0.02)	1.87 (0.05)
44	204[M+](38) 105(100) 93(96)	1495	ND	0.16 (0.02)	0.18 (0.05)	0.21 (0.03)	0.24 (0.05)	0.26 (0.05)	0.27 (0.03)
45	bicyclogermacrene	1499 ^a,b,c^	1500 ^c^	17.63 (0.04)	18.02 (0.05)	17.54 (0.06)	17.43 (0.03)	17.73 (0.03)	16.27 (0.03)
46	204[M+](19) 93(100) 91(95)	1505	ND	0.38 (0.01)	0.55 (0.03)	0.57 (0.03)	0.61 (0.04)	0.37 (0.04)	0.46 (0.02)
47	202[M+](25) 133(100) 91(89)	1509	ND	0.20 (0.03)	0.09 (0.03)	0.11 (0.02)	0.12 (0.03)	0.13 (0.03)	0.15 (0.01)
48	206[M+](14) 191(100) 57(38)	1514	ND	0.09 (0.03)	0.13 (0.04)	0.15 (0.04)	0.08 (0.04)	0.25 (0.04)	0.31 (0.05)
49	202[M+](33) 131(100) 145(53)	1518	ND	0.50 (0.04)	0.44 (0.03)	0.43 (0.04)	0.37 (0.03)	0.35 (0.03)	0.45 (0.04)
50	δ-cadinene	1524 ^a,b,c^	1522 ^c^	0.09 (0.01)	0.13 (0.03)	0.14 (0.01)	0.17 (0.02)	0.12 (0.02)	0.13 (0.02)
51	204[M+](5) 91(100) 131(95)	1530	ND	0.16 (0.04)	0.17 (0.02)	0.19 (0.03)	0.14 (0.04)	0.16 (0.04)	0.18 (0.04)
52	200[M+](54) 185(100) 143(91)	1535	ND	-	-	-	-	-	-
53	4,5,9,10-dehydro-isolongifolene	1544 ^a,b^	1544 ^a^	4.28 (0.04)	4.35 (0.04)	4.29 (0.03)	4.57 (0.05)	4.51 (0.04)	4.32 (0.06)
54	202[M+](4) 128(100) 157(95)	1547	ND	0.74 (0.03)	0.75 (0.03)	0.68 (0.04)	0.83 (0.03)	0.78 (0.03)	0.71 (0.03)
55	200[M+](8) 171(100) 186(79)	1551	ND	0.09 (0.02)	0.08 (0.01)	0.11 (0.01)	0.09 (0.01)	0.08 (0.02)	0.09 (0.04)
56	200[M+](91) 129(100) 157(88)	1556	ND	0.06 (0.03)	0.04 (0.01)	0.09 (0.04)	0.03 (0.03)	0.06 (0.01)	0.06 (0.01)
57	204[M+](8) 143(100) 157(98)	1559	ND	0.06 (0.01)	0.02 (0.01)	0.07 (0.01)	0.06 (0.03)	0.05 (0.01)	0.06 (0.01)
58	204[M+](82) 173(100) 189(94)	1563	ND	1.20 (0.04)	1.25 (0.03)	1.23 (0.04)	1.19 (0.04)	1.20 (0.03)	1.22 (0.03)
59	palustrol	1567 ^c^	1567 ^c^	5.32 (0.04)	5.43 (0.03)	5.22 (0.04)	5.09 (0.01)	5.49 (0.03)	5.23 (0.04)
60	200[M+](11) 79(100) 93(95)	1570	ND	0.41 (0.03)	0.46 (0.04)	0.53 (0.03)	0.50 (0.03)	0.61 (0.04)	0.43 (0.02)
61	204[M+](31) 81(100) 109(88)	1573	ND	2.32 (0.02)	1.79 (0.04)	1.95 (0.02)	1.87 (0.03)	2.14 (0.03)	2.29 (0.03)
62	spathulenol	1576 ^a,b,c^	1577 ^c^	2.62 (0.05)	2.69 (0.06)	2.57 (0.05)	2.72 (0.04)	2.67 (0.03)	2.63 (0.03)
63	200[M+](56) 185(100) 143(63)	1581	ND	2.41 (0.04)	2.39 (0.03)	2.57 (0.04)	2.45 (0.01)	2.58 (0.02)	2.44 (0.04)
64	202[M+](4) 91(100) 79(82)	1587	ND	0.30 (0.04)	0.27 (0.03)	0.37 (0.02)	0.26 (0.04)	0.30 (0.04)	0.31 (0.03)
65	globulol	1599 ^a,b,c,d^	1590 ^c^	1.33 (0.02)	1.46 (0.02)	1.27 (0.03)	1.39 (0.03)	1.49 (0.03)	1.50 (0.04)
66	200[M+](8) 198(100) 183(84)	1605	ND	-	-	-	-	-	-
67	220[M+](2) 145(100) 200(93)	1609	ND	0.11 (0.03)	0.08 (0.02)	0.10 (0.02)	0.09 (0.04)	0.08 (0.02)	0.06 (0.03)
68	(+)-bisabola-2,10-diene[1,9]oxide	1615 ^a,b^	1596 ^a^	0.30 (0.03)	0.29 (0.03)	0.31 (0.03)	0.32 (0.04)	0.28 (0.05)	0.27 (0.02)
69	208[M+](3) 95(100) 85(95)	1621	ND	0.62 (0.03)	0.63 (0.04)	0.70 (0.03)	0.46 (0.03)	0.53 (0.04)	0.61 (0.02)
70	ledene oxide-(II)	1631 ^a,b^	1631 ^a^	-	-	-	-	-	-
71	isospathulenol	1635 ^a,b^	1633 ^a^	0.32 (0.04)	0.38 (0.04)	0.34 (0.03)	0.29 (0.02)	0.39 (0.02)	0.37 (0.01)
72	220[M+](18) 91(100) 105(83)	1639	ND	0.78 (0.04)	0.75 (0.06)	0.71 (0.03)	0.73 (0.03)	0.77 (0.03)	0.75 (0.03)
73	cubenol	1642 ^a,b,c,d^	1645 ^c^	0.11 (0.06)	0.10 (0.03)	0.08 (0.02)	0.07 (0.01)	0.09 (0.02)	0.10 (0.03)
74	220[M+](21) 91(100) 105(82)	1651	ND	0.04 (0.03)	0.05 (0.03)	0.04 (0.01)	0.03 (0.01)	0.05 (0.01)	0.03 (0.01)
75	222[M+](3) 179(100) 121(92)	1655	ND	0.05 (0.03)	0.04 (0.01)	0.03 (0.01)	0.06 (0.01)	0.07 (0.01)	0.02 (0.01)
76	germacra-4(15),5,10(14)-trien-1-α-ol	1660 ^c^	1685 ^c^	0.87 (0.04)	0.90 (0.03)	0.87 (0.04)	0.92 (0.03)	0.95 (0.01)	0.83 (0.04)
77	216[M+](31) 145(100) 91(97)	1699	ND	0.15 (0.04)	0.17 (0.04)	0.19 (0.02)	0.20 (0.03)	0.22 (0.04)	0.25 (0.04)
78	1,4-dimethyl-7-(1-methylethyl)-azulene	1790 ^c^	1779 ^c^	1.07 (0.05)	1.16 (0.02)	1.24 (0.03)	1.29 (0.04)	1.33 (0.06)	1.09 (0.04)
79	14-hydroxy-δ-cadinene	1797 ^c^	1803 ^c^	0.16 (0.01)	0.17 (0.02)	0.05 (0.01)	0.08 (0.04)	0.11 (0.03)	0.16 (0.04)
	Total			98.83 (2.30)	98.74 (2.18)	98.57 (2.18)	98.93 (2.13)	99.71 (2.13)	98.50 (2.13)
	% identified			84.69 (1.29)	84.80 (1.20)	83.87 (1.26)	85.10 (1.17)	85.17 (1.17)	83.73 (1.23)
	including:								
	aliphatics			1.27 (0.14)	1.27 (0.13)	1.20 (0.17)	1.32 (0.14)	1.32 (0.17)	1.34 (0.16)
	aromatics			2.41 (0.15)	2.30 (0.17)	2.36 (0.13)	2.44 (0.12)	2.49 (0.15)	2.55 (0.14)
	monoterpene hydrocarbons			0.01 (0.01)	0.01 (0.00)	0.01 (0.01)	0.02 (0.04)	0.03 (0.01)	0.04 (0.01)
	monoterpenoid hydrocarbons			0.02 (0.01)	0.04 (0.01)	0.05 (0.01)	0.03 (0.03)	0.02 (0.01)	0.06 (0.01)
	sesquiterpene hydrocarbons			69.95 (0.69)	69,76 (0.63)	69.54 (0.69)	70.41 (0.62)	69.84 (0.61)	68.65 (0.62)
	sesquiterpenoid hydrocarbons			11.03 (0.29)	11.42 (0.26)	10.71 (0.25)	10.88 (0.22)	11.47 (0.22)	11.09 (0.25)

- less than 0.01%. * The names of terpenes and terpenoids according to IUPAC terminology are given in Table S5. ** Retention index on Quadex 007-5MS column. *** Literature retention index. ND, no data. **** For abbreviations of samples, see Table 1. Standard deviation in brackets. Identification of compounds by MS databases (^a^ NIST 2011, ^b^ NIST Chemistry WebBook, ^c^ Adams 4 Library, ^d^ Pherobase).

**Table 4:** (**a**) Volatile compounds detected in the samples collected in autumn (CI-25–CI-30). (**b**) Volatile compounds detected in the samples collected in autumn (CI-31–CI-36).

(a)									
No.	Compounds *	RI **	RI ***	Code ****					
				CI-25	CI-26	CI-27	CI-28	CI-29	CI-30
1	propan-1-ol	<700 ^a,b^	483 ^a^	1.42 (0.03)	1.23 (0.03)	1.32 (0.03)	1.43 (0.03)	1.36 (0.05)	1.45 (0.05)
2	pentanal	705 ^a,b,c,d^	704 ^c^	0.39 (0.04)	0.45 (0.01)	0.41 (0.03)	0.39 (0.04)	0.42 (0.03)	0.47 (0.03)
3	hexanal	802 ^a,b,c,d^	801 ^c^	0.43 (0.02)	0.39 (0.01)	0.42 (0.03)	0.43 (0.02)	0.40 (0.05)	0.38 (0.04)
4	hexan-1-ol	867 ^a,b^	869 ^a^	0.32 (0.03)	0.29 (0.04)	0.26 (0.04)	0.25 (0.04)	0.30 (0.03)	0.31 (0.03)
5	heptanal	902 ^a,b,c,d^	901 ^c^	0.03 (0.01)	0.02 (0.01)	0.05 (0.01)	0.01 (0.01)	0.03 (0.01)	0.02 (0.01)
6	α-pinene	939 ^a,b,c^	932 ^c^	0.02 (0.01)	0.05 (0.02)	0.05 (0.01)	0.04 (0.01)	0.03 (0.01)	0.04 (0.01)
7	benzaldehyde	940 ^a,b,c^	952 ^c^	0.53 (0.04)	0.48 (0.03)	0.41 (0.04)	0.44 (0.02)	0.45 (0.02)	0.43 (0.03)
8	2-ethylhexan-1-ol	1023 ^a,b^	1025 ^a^	0.16 (0.02)	0.21 (0.03)	0.25 (0.02)	0.17 (0.02)	0.20 (0.05)	0.23 (0.03)
9	phenylmethanol	1028 ^a,b,c^	1026 ^c^	1.08 (0.04)	0.98 (0.03)	1.02 (0.02)	1.00 (0.04)	0.99 (0.03)	1.03 (0.02)
10	phenylacetaldehyde	1044 ^a,b^	1044 ^a^	1.75 (0.03)	1.65 (0.03)	1.72 (0.03)	1.70 (0.02)	1.68 (0.03)	1.75 (0.03)
11	nonanal	1102 ^a,b,c,d^	1100 ^c^	0.12 (0.01)	0.13 (0.04)	0.10 (0.02)	0.11 (0.04)	0.09 (0.02)	0.10 (0.03)
12	3,4-dimethylcyclohexan-1-ol	1115 ^a,b^	1126 ^a^	0.09 (0.02)	0.12 (0.02)	0.09 (0.01)	0.08 (0.02)	0.10 (0.03)	0.11 (0.04)
13	phenylethanol	1121 ^a,b^	1121 ^a^	0.60 (0.05)	0.57 (0.04)	0.55 (0.04)	0.59 (0.03)	0.57 (0.03)	0.61 (0.03)
14	decanal	1195 ^a,b,c,d^	1201 ^c^	0.07 (0.01)	0.06 (0.01)	0.09 (0.01)	0.05 (0.01)	0.07 (0.01)	0.09 (0.02)
15	β-cyclocitral	1221 ^c^	1217 ^c^	0.07 (0.01)	0.03 (0.01)	0.05 (0.01)	0.04 (0.01)	0.03 (0.01)	0.05 (0.01)
16	2-phenoxyethan-1-ol	1225 ^a,b^	1226 ^a^	1.28 (0.05)	1.14 (0.02)	1.32 (0.05)	1.15 (0.04)	1.30 (0.04)	1.29 (0.02)
17	bicycloelemene	1316 ^a^	1330 ^a^	0.05 (0.01)	0.09 (0.02)	0.08 (0.02)	0.08 (0.02)	0.10 (0.03)	0.06 (0.01)
18	δ-elemene	1324 ^a,b,c^	1335 ^c^	0.74 (0.05)	0.69 (0.04)	0.71 (0.04)	0.68 (0.05)	0.70 (0.02)	0.73 (0.05)
19	204[M+](5) 121(100) 93(89)	1343	ND	0.11 (0.03)	0.15 (0.02)	0.12 (0.02)	0.10 (0.02)	0.13 (0.05)	0.15 (0.02)
20	200[M+](39) 159(100) 117(95)	1345	ND	1.33 (0.05)	1.27 (0.04)	1.22 (0.05)	1.30 (0.03)	1.25 (0.05)	1.26 (0.04)
21	202[M+](13) 81(100) 96(73)	1350	ND	0.09 (0.02)	0.14 (0.03)	0.12 (0.02)	0.10 (0.03)	0.11 (0.02)	0.09 (0.01)
22	204[M+](10) 119(100) 91(84)	1353	ND	0.08 (0.02)	0.11 (0.01)	0.09 (0.01)	0.09 (0.01)	0.10 (0.04)	0.11 (0.03)
23	anastreptene	1370 ^a^	1370 ^a^	15.73 (0.05)	15.98 (0.05)	15.51 (0.06)	16.01 (0.03)	15.48 (0.04)	15.27 (0.04)
24	204[M+](5) 81(100) 93(96)	1384	ND	0.12 (0.03)	0.23 (0.03)	0.18 (0.01)	0.13 (0.04)	0.15 (0.03)	0.20 (0.03)
25	β-elemene	1391 ^a,b,c^	1389 ^c^	1.32 (0.03)	1.24 (0.04)	1.29 (0.02)	1.30 (0.04)	1.25 (0.04)	1.27 (0.05)
26	204[M+](13) 157(100) 185(84)	1398	ND	0.19 (0.02)	0.28 (0.02)	0.23 (0.04)	0.18 (0.03)	0.20 (0.03)	0.23 (0.03)
27	204[M+](13) 157(100) 185(84)	1417	ND	0.34 (0.03)	0.31 (0.05)	0.37 (0.04)	0.33 (0.02)	0.30 (0.05)	0.35 (0.01)
28	204[M+](19) 135(100) 105(82)	1423	ND	0.19 (0.02)	0.14 (0.02)	0.12 (0.03)	0.11 (0.05)	0.13 (0.03)	0.19 (0.03)
29	204[M+](9) 91(100) 105(93)	1425	ND	0.04 (0.01)	0.06 (0.01)	0.03 (0.01)	0.01 (0.01)	0.03 (0.01)	0.05 (0.01)
30	(-)-aristolene	1429 ^a,b,c,d^	1428 ^a^	1.12 (0.04)	1.23 (0.03)	1.18 (0.05)	1.21 (0.02)	1.19 (0.03)	1.11 (0.03)
31	204[M+](9) 107(100) 79(43)	1432	ND	0.16 (0.04)	0.09 (0.01)	0.11 (0.05)	0.10 (0.04)	0.11 (0.02)	0.12 (0.02)
32	γ-maaliene	1435 ^a,b^	1427 ^a^	0.28 (0.03)	0.18 (0.02)	0.23 (0.02)	0.25 (0.03)	0.19 (0.03)	0.23 (0.04)
33	α-maaliene	1443 ^a,b^	1442 ^a^	0.18 (0.02)	0.27 (0.04)	0.29 (0.04)	0.19 (0.03)	0.25 (0.02)	0.23 (0.03)
34	aromandendrene	1445 ^a,b^	1447 ^a^	3.35 (0.05)	3.15 (0.04)	3.21 (0.02)	3.23 (0.04)	3.21 (0.04)	3.18 (0.04)
35	selina-5,11-diene	1447 ^a,b^	1454 ^a^	0.31 (0.05)	0.26 (0.03)	0.32 (0.04)	0.33 (0.03)	0.27 (0.03)	0.30 (0.03)
36	dehydroaromadendrene	1456 ^c^	1460 ^c^	1.05 (0.02)	1.24 (0.02)	1.15 (0.04)	1.12 (0.05)	1.06 (0.04)	1.16 (0.04)
37	1,2,9,10-tetradehydroaristolane	1461	ND	0.44 (0.04)	0.54 (0.05)	0.47 (0.03)	0.45 (0.03)	0.51 (0.03)	0.47 (0.03)
38	204[M+](15) 91(100) 105(84)	1465	ND	0.38 (0.03)	0.26 (0.05)	0.21 (0.02)	0.25 (0.02)	0.27 (0.04)	0.33 (0.02)
39	204[M+](18) 128(100) 143(95)	1469	ND	0.32 (0.03)	0.36 (0.02)	0.39 (0.03)	0.33 (0.03)	0.39 (0.03)	0.34 (0.02)
40	γ-gurjunene	1474 ^c,d^	1475 ^c^	0.34 (0.04)	0.28 (0.04)	0.33 (0.05)	0.29 (0.02)	0.34 (0.02)	0.33 (0.02)
41	γ-muurolene	1477 ^c^	1478 ^c^	0.14 (0.03)	0.09 (0.03)	0.11 (0.02)	0.09 (0.01)	0.12 (0.02)	0.08 (0.01)
42	δ-selinene	1488 ^c^	1492 ^c^	1.08 (0.05)	0.98 (0.03)	1.12 (0.04)	1.00 (0.04)	1.11 (0.02)	1.09 (0.01)
43	ledene	1492 ^a,b,c^	1496 ^c^	1.59 (0.03)	1.21 (0.04)	1.38 (0.03)	1.47 (0.04)	1.55 (0.05)	1.38 (0.04)
44	204[M+](38) 105(100) 93(96)	1495	ND	0.08 (0.02)	0.09 (0.03)	0.05 (0.01)	0.06 (0.01)	0.09 (0.01)	0.10 (0.03)
45	bicyclogermacrene	1499 ^a,b,c^	1500 ^c^	7.87 (0.03)	7.23 (0.05)	6.99 (0.04)	7.59 (0.04)	7.68 (0.04)	7.36 (0.05)
46	204[M+](19) 93(100) 91(95)	1505	ND	0.09 (0.02)	0.19 (0.03)	0.14 (0.03)	0.10 (0.03)	0.13 (0.03)	0.18 (0.01)
47	202[M+](25) 133(100) 91(89)	1509	ND	0.24 (0.03)	0.13 (0.02)	0.16 (0.02)	0.19 (0.04)	0.21 (0.05)	0.16 (0.02)
48	206[M+](14) 191(100) 57(38)	1514	ND	0.17 (0.02)	0.09 (0.03)	0.15 (0.02)	0.11 (0.03)	0.09 (0.01)	0.16 (0.04)
49	202[M+](33) 131(100) 145(53)	1518	ND	0.21 (0.04)	0.14 (0.02)	0.20 (0.02)	0.19 (0.02)	0.16 (0.02)	0.21 (0.02)
50	δ-cadinene	1524 ^a,b,c^	1522^c^	0.38 (0.03)	0.29 (0.03)	0.34 (0.03)	0.30 (0.05)	0.33 (0.04)	0.36 (0.02)
51	204[M+](5) 91(100) 131(95)	1530	ND	0.12 (0.04)	0.06 (0.01)	0.08 (0.02)	0.09 (0.01)	0.07 (0.02)	0.11 (0.04)
52	200[M+](54) 185(100) 143(91)	1535	ND	0.20 (0.03)	0.16 (0.04)	0.22 (0.03)	0.21 (0.04)	0.18 (0.02)	0.23 (0.03)
53	4,5,9,10-dehydro-isolongifolene	1544 ^a,b^	1544 ^a^	8.71 (0.04)	8.03 (0.05)	8.53 (0.02)	8.24 (0.05)	8.47 (0.04)	8.68 (0.04)
54	202[M+](4) 128(100) 157(95)	1547	ND	1.43 (0.03)	1.63 (0.04)	1.54 (0.04)	1.44 (0.05)	1.61 (0.03)	1.59 (0.05)
55	200[M+](8) 171(100) 186(79)	1551	ND	0.22 (0.02)	0.17 (0.03)	0.18 (0.05)	0.21 (0.03)	0.19 (0.02)	0.16 (0.01)
56	200[M+](91) 129(100) 157(88)	1556	ND	0.09 (0.02)	0.11 (0.04)	0.13 (0.04)	0.10 (0.02)	0.11 (0.05)	0.09 (0.02)
57	204[M+](8) 143(100) 157(98)	1559	ND	0.05 (0.02)	0.03 (0.03)	0.08 (0.03)	0.06 (0.04)	0.04 (0.01)	0.05 (0.01)
58	204[M+](82) 173(100) 189(94)	1563	ND	1.55 (0.05)	1.65 (0.02)	1.62 (0.04)	1.56 (0.03)	1.64 (0.02)	1.63 (0.02)
59	palustrol	1567 ^c^	1567 ^c^	9.79 (0.05)	9.86 (0.05)	9.93 (0.05)	9.81 (0.03)	9.89 (0.02)	9.91 (0.05)
60	200[M+](11) 79(100) 93(95)	1570	ND	0.57 (0.04)	0.46 (0.05)	0.51 (0.03)	0.45 (0.04)	0.50 (0.02)	0.56 (0.05)
61	204[M+](31) 81(100) 109(88)	1573	ND	0.90 (0.03)	0.87 (0.04)	0.92 (0.03)	0.89 (0.03)	0.93 (0.05)	0.86 (0.06)
62	spathulenol	1576 ^a,b,c^	1577 ^c^	4.96 (0.05)	5.01 (0.03)	5.06 (0.04)	5.00 (0.02)	4.98 (0.05)	5.03 (0.03)
63	200[M+](56) 185(100) 143(63)	1581	ND	5.64 (0.05)	5.24 (0.05)	5.43 (0.03)	5.36 (0.05)	5.48 (0.06)	5.57 (0.04)
64	202[M+](4) 91(100) 79(82)	1587	ND	0.51 (0.02)	0.46 (0.03)	0.48 (0.02)	0.44 (0.03)	0.52 (0.03)	0.49 (0.02)
65	globulol	1599 ^a,b,c,d^	1590 ^c^	2.97 (0.04)	3.06 (0.02)	3.15 (0.03)	3.00 (0.02)	2.98 (0.04)	3.05 (0.05)
66	200[M+](8) 198(100) 183(84)	1605	ND	0.28 (0.03)	0.35 (0.04)	0.21 (0.04)	0.19 (0.04)	0.29 (0.02)	0.27 (0.03)
67	220[M+](2) 145(100) 200(93)	1609	ND	2.22 (0.01)	2.01 (0.03)	2.14 (0.04)	2.18 (0.02)	2.14 (0.05)	1.99 (0.06)
68	(+)-bisabola-2,10-diene[1,9]oxide	1615 ^a,b^	1596 ^a^	0.22 (0.04)	0.16 (0.03)	0.13 (0.02)	0.18 (0.05)	0.20 (0.05)	0.21 (0.03)
69	208[M+](3) 95(100) 85(95)	1621	ND	1.09 (0.03)	1.57 (0.04)	1.38 (0.04)	1.10 (0.05)	1.22 (0.06)	1.48 (0.04)
70	ledene oxide-(II)	1631 ^a,b^	1631 ^a^	0.22 (0.02)	0.16 (0.03)	0.27 (0.03)	0.26 (0.06)	0.16 (0.03)	0.18 (0.03)
71	isospathulenol	1635 ^a,b^	1633 ^a^	0.86 (0.05)	0.94 (0.02)	0.99 (0.04)	1.01 (0.03)	0.95 (0.04)	0.87 (0.02)
72	220[M+](18) 91(100) 105(83)	1639	ND	1.56 (0.03)	1.64 (0.05)	1.52 (0.03)	1.62 (0.04)	1.57 (0.03)	1.60 (0.03)
73	cubenol	1642 ^a,b,c,d^	1645 ^c^	0.54 (0.02)	0.44 (0.03)	0.41 (0.02)	0.55 (0.03)	0.43 (0.02)	0.39 (0.03)
74	220[M+](21) 91(100) 105(82)	1651	ND	0.10 (0.02)	0.10 (0.02)	0.08 (0.02)	0.09 (0.02)	0.08 (0.01)	0.11 (0.04)
75	222[M+](3) 179(100) 121(92)	1655	ND	0.05 (0.02)	0.05 (0.01)	0.03 (0.01)	0.02 (0.01)	0.04 (0.01)	0.06 (0.02)
76	germacra-4(15),5,10(14)-trien-1-α-ol	1660 ^c^	1685 ^c^	0.99 (0.05)	0.83 (0.02)	1.02 (0.04)	1.01 (0.03)	0.95 (0.02)	0.87 (0.04)
77	216[M+](31) 145(100) 91(97)	1699	ND	0.61 (0.05)	0.53 (0.05)	0.67 (0.03)	0.55 (0.02)	0.60 (0.03)	0.64 (0.05)
78	1,4-dimethyl-7-(1-methylethyl)-azulene	1790 ^c^	1779 ^c^	3.02 (0.06)	3.56 (0.05)	3.23 (0.02)	3.11 (0.01)	3.45 (0.02)	3.27 (0.06)
79	14-hydroxy-δ-cadinene	1797 ^c^	1803 ^c^	0.40 (0.03)	0.36 (0.06)	0.41 (0.05)	0.39 (0.02)	0.42 (0.04)	0.37 (0.03)
	Total			98.34 (2.47)	96.29 (2.43)	97.06 (2.35)	96.27 (2.35)	97.30 (2.42)	97.52 (2.39)
	% identified			77.01 (1.47)	75.16 (1.37)	75.95 (1.35)	76.03 (1.32)	76.24 (1.36)	75.80 (1.38)
	including:								
	aliphatics			3.03 (0.19)	2.90 (0.20)	2.99 (0.20)	2.92 (0.23)	2.97 (0.28)	3.16 (0.28)
	aromatics			5.24 (0.21)	4.82 (0.15)	5.02 (0.18)	4.88 (0.15)	4.99 (0.15)	5.11 (0.13)
	monoterpene hydrocarbons			0.02 (0.01)	0.05 (0.02)	0.05 (0.01)	0.04 (0.01)	0.03 (0.01)	0.04 (0.01)
	monoterpenoid hydrocarbons			0.07 (0.01)	0.03 (0.01)	0.05 (0.01)	0.04 (0.01)	0.03 (0.01)	0.05 (0.01)
	sesquiterpene hydrocarbons			47.70 (0.70)	46.54 (0.70)	46.47 (0.63)	46.94 (0.63)	47.26 (0.60)	46.56 (0.64)
	sesquiterpenoid hydrocarbons			20.95 (0.35)	20.82 (0.29)	21.37 (0.32)	21.21 (0.29)	20.96 (0.31)	20.88 (0.31)
(**b**)									
**No.**	Compounds *	RI **	RI ***	Code ****					
				**CI-31**	**CI-32**	**CI-33**	**CI-34**	**CI-35**	**CI-36**
1	propan-1-ol	<700 ^a,b^	483 ^a^	1.37 (0.03)	1.21 (0.04)	1.33 (0.05)	1.41 (0.04)	1.39 (0.04)	1.30 (0.05)
2	pentanal	705 ^a,b,c,d^	704 ^c^	0.43 (0.03)	0.40 (0.03)	0.36 (0.05)	0.46 (0.02)	0.42 (0.03)	0.41 (0.03)
3	hexanal	802 ^a,b,c,d^	801 ^c^	0.42 (0.02)	0.45 (0.03)	0.39 (0.04)	0.40 (0.02)	0.42 (0.01)	0.43 (0.01)
4	hexan-1-ol	867 ^a,b^	869 ^a^	0.27 (0.04)	0.29 (0.04)	0.33 (0.03)	0.29 (0.03)	0.27 (0.03)	0.26 (0.02)
5	heptanal	902 ^a,b,c,d^	901 ^c^	0.04 (0.01)	0.03 (0.01)	0.05 (0.01)	0.01 (0.01)	0.04 (0.01)	0.05 (0.01)
6	α-pinene	939 ^a,b,c^	932 ^c^	0.05 (0.01)	0.02 (0.01)	0.03 (0.01)	0.04 (0.01)	0.05 (0.01)	0.05 (0.01)
7	benzaldehyde	940 ^a,b,c^	952 ^c^	0.48 (0.03)	0.50 (0.03)	0.49 (0.05)	0.52 (0.04)	0.47 (0.03)	0.53 (0.03)
8	2-ethylhexan-1-ol	1023 ^a,b^	1025 ^a^	0.25 (0.04)	0.19 (0.02)	0.18 (0.03)	0.21 (0.03)	0.25 (0.04)	0.19 (0.03)
9	phenylmethanol	1028 ^a,b,c^	1026 ^c^	1.10 (0.04)	1.11 (0.04)	0.97 (0.06)	1.03 (0.03)	1.06 (0.02)	1.01 (0.04)
10	phenylacetaldehyde	1044 ^a,b^	1044 ^a^	1.63 (0.03)	1.69 (0.04)	1.71 (0.03)	1.74 (0.05)	1.67 (0.03)	1.70 (0.03)
11	nonanal	1102 ^a,b,c,d^	1100 ^c^	0.12 (0.04)	0.11 (0.03)	0.13 (0.04)	0.14 (0.02)	0.09 (0.02)	0.10 (0.03)
12	3,4-dimethylcyclohexan-1-ol	1115 ^a,b^	1126 ^a^	0.09 (0.02)	0.08 (0.01)	0.13 (0.03)	0.10 (0.03)	0.09 (0.01)	0.10 (0.01)
13	phenylethanol	1121 ^a,b^	1121 ^a^	0.60 (0.03)	0.62 (0.05)	0.59 (0.02)	0.55 (0.02)	0.54 (0.05)	0.57 (0.02)
14	decanal	1195 ^a,b,c,d^	1201 ^c^	0.10 (0.02)	0.09 (0.02)	0.07 (0.01)	0.05 (0.01)	0.06 (0.01)	0.07 (0.01)
15	β-cyclocitral	1221 ^c^	1217 ^c^	0.07 (0.01)	0.06 (0.01)	0.05 (0.01)	0.03 (0.01)	0.03 (0.01)	0.05 (0.01)
16	2-phenoxyethan-1-ol	1225 ^a,b^	1226 ^a^	1.16 (0.05)	1.11 (0.02)	1.35 (0.04)	1.29 (0.03)	1.31 (0.02)	1.21 (0.02)
17	bicycloelemene	1316 ^a^	1330 ^a^	0.07 (0.02)	0.08 (0.01)	0.06 (0.01)	0.04 (0.01)	0.05 (0.01)	0.09 (0.02)
18	δ-elemene	1324 ^a,b,c^	1335 ^c^	0.69 (0.05)	0.67 (0.02)	0.74 (0.04)	0.73 (0.02)	0.71 (0.03)	0.69 (0.05)
19	204[M+](5) 121(100) 93(89)	1343	ND	0.13 (0.02)	0.11 (0.02)	0.12 (0.05)	0.13 (0.02)	0.15 (0.01)	0.14 (0.02)
20	200[M+](39) 159(100) 117(95)	1345	ND	1.25 (0.03)	1.27 (0.06)	1.32 (0.06)	1.29 (0.04)	1.31 (0.05)	1.30 (0.04)
21	202[M+](13) 81(100) 96(73)	1350	ND	0.12 (0.03)	0.15 (0.03)	0.13 (0.03)	0.10 (0.04)	0.13 (0.04)	0.14 (0.04)
22	204[M+](10) 119(100) 91(84)	1353	ND	0.08 (0.01)	0.09 (0.02)	0.09 (0.02)	0.10 (0.03)	0.11 (0.04)	0.09 (0.01)
23	anastreptene	1370 ^a^	1370 ^a^	15.72 (0.05)	15.64 (0.04)	15.34 (0.04)	15.28 (0.03)	15.69 (0.04)	15.70 (0.04)
24	204[M+](5) 81(100) 93(96)	1384	ND	0.25 (0.04)	0.19 (0.03)	0.18 (0.03)	0.13 (0.05)	0.15 (0.02)	0.21 (0.03)
25	β-elemene	1391 ^a,b,c^	1389 ^c^	1.29 (0.04)	1.27 (0.03)	1.33 (0.03)	1.35 (0.05)	1.31 (0.05)	1.29 (0.05)
26	204[M+](13) 157(100) 185(84)	1398	ND	0.25 (0.03)	0.21 (0.02)	0.19 (0.02)	0.22 (0.02)	0.25 (0.04)	0.27 (0.03)
27	204[M+](13) 157(100) 185(84)	1417	ND	0.36 (0.02)	0.31 (0.02)	0.30 (0.02)	0.34 (0.04)	0.36 (0.03)	0.37 (0.01)
28	204[M+](19) 135(100) 105(82)	1423	ND	0.20 (0.05)	0.18 (0.05)	0.15 (0.05)	0.14 (0.02)	0.16 (0.04)	0.18 (0.03)
29	204[M+](9) 91(100) 105(93)	1425	ND	0.06 (0.01)	0.04 (0.01)	0.04 (0.01)	0.02 (0.01)	0.03 (0.01)	0.02 (0.01)
30	(-)-aristolene	1429 ^a,b,c,d^	1428 ^a^	1.20 (0.03)	1.19 (0.04)	1.25 (0.04)	1.19 (0.04)	1.18 (0.03)	1.22 (0.03)
31	204[M+](9) 107(100) 79(43)	1432	ND	0.15 (0.04)	0.14 (0.02)	0.11 (0.02)	0.13 (0.03)	0.16 (0.04)	0.15 (0.02)
32	γ-maaliene	1435 ^a,b^	1427 ^a^	0.19 (0.03)	0.20 (0.04)	0.23 (0.04)	0.27 (0.02)	0.25 (0.03)	0.17 (0.04)
33	α-maaliene	1443 ^a,b^	1442 ^a^	0.30 (0.03)	0.29 (0.04)	0.21 (0.04)	0.24 (0.03)	0.27 (0.05)	0.28 (0.03)
34	aromandendrene	1445 ^a,b^	1447 ^a^	3.16 (0.04)	3.37 (0.04)	3.30 (0.04)	3.29 (0.05)	3.18 (0.03)	3.33 (0.04)
35	selina-5,11-diene	1447 ^a,b^	1454 ^a^	0.32 (0.03)	0.29 (0.02)	0.26 (0.02)	0.27 (0.02)	0.31 (0.02)	0.26 (0.03)
36	dehydroaromadendrene	1456 ^c^	1460 ^c^	1.19 (0.05)	1.21 (0.05)	1.23 (0.05)	1.27 (0.04)	1.08 (0.03)	1.11 (0.04)
37	1,2,9,10-tetradehydroaristolane	1461	ND	0.49 (0.03)	0.50 (0.05)	0.53 (0.03)	0.46 (0.03)	0.43 (0.02)	0.41 (0.03)
38	204[M+](15) 91(100) 105(84)	1465	ND	0.31 (0.02)	0.29 (0.02)	0.36 (0.02)	0.24 (0.02)	0.39 (0.03)	0.26 (0.02)
39	204[M+](18) 128(100) 143(95)	1469	ND	0.29 (0.03)	0.38 (0.04)	0.40 (0.03)	0.36 (0.02)	0.32 (0.04)	0.38 (0.02)
40	γ-gurjunene	1474 ^c,d^	1475 ^c^	0.29 (0.02)	0.28 (0.04)	0.34 (0.02)	0.33 (0.03)	0.31 (0.04)	0.27 (0.02)
41	γ-muurolene	1477 ^c^	1478 ^c^	0.13 (0.03)	0.10 (0.03)	0.13 (0.03)	0.15 (0.02)	0.09 (0.01)	0.11 (0.05)
42	δ-selinene	1488 ^c^	1492 ^c^	1.07 (0.04)	0.99 (0.04)	1.02 (0.04)	0.96 (0.02)	1.05 (0.05)	1.14 (0.03)
43	ledene	1492 ^a,b,c^	1496 ^c^	1.33 (0.04)	1.18 (0.03)	1.57 (0.03)	1.46 (0.02)	1.35 (0.03)	1.29 (0.04)
44	204[M+](38) 105(100) 93(96)	1495	ND	0.06 (0.01)	0.09 (0.01)	0.04 (0.01)	0.11 (0.03)	0.08 (0.01)	0.07 (0.01)
45	bicyclogermacrene	1499 ^a,b,c^	1500 ^c^	7.48 (0.05)	7.83 (0.03)	7.16 (0.03)	7.79 (0.02)	7.64 (0.03)	7.75 (0.05)
46	204[M+](19) 93(100) 91(95)	1505	ND	0.20 (0.03)	0.16 (0.02)	0.14 (0.02)	0.13 (0.03)	0.14 (0.02)	0.16 (0.01)
47	202[M+](25) 133(100) 91(89)	1509	ND	0.23 (0.04)	0.17 (0.03)	0.20 (0.04)	0.15 (0.02)	0.17 (0.05)	0.23 (0.02)
48	206[M+](14) 191(100) 57(38)	1514	ND	0.18 (0.03)	0.14 (0.02)	0.12 (0.03)	0.09 (0.02)	0.12 (0.01)	0.10 (0.04)
49	202[M+](33) 131(100) 145(53)	1518	ND	0.17 (0.02)	0.18 (0.03)	0.19 (0.04)	0.20 (0.05)	0.22 (0.04)	0.18 (0.02)
50	δ-cadinene	1524 ^a,b,c^	1522 ^c^	0.28 (0.05)	0.30 (0.02)	0.31 (0.03)	0.27 (0.04)	0.36 (0.05)	0.38 (0.02)
51	204[M+](5) 91(100) 131(95)	1530	ND	0.05 (0.01)	0.09 (0.01)	0.10 (0.04)	0.13 (0.03)	0.12 (0.03)	0.11 (0.04)
52	200[M+](54) 185(100) 143(91)	1535	ND	0.25 (0.04)	0.17 (0.02)	0.19 (0.03)	0.17 (0.04)	0.22 (0.03)	0.19 (0.03)
53	4,5,9,10-dehydro-isolongifolene	1544 ^a,b^	1544 ^a^	8.70 (0.05)	8.64 (0.04)	8.59 (0.02)	8.43 (0.05)	8.66 (0.02)	8.72 (0.04)
54	202[M+](4) 128(100) 157(95)	1547	ND	1.48 (0.03)	1.59 (0.03)	1.43 (0.02)	1.55 (0.04)	1.65 (0.04)	1.55 (0.03)
55	200[M+](8) 171(100) 186(79)	1551	ND	0.19 (0.03)	0.20 (0.03)	0.23 (0.02)	0.19 (0.01)	0.18 (0.03)	0.17 (0.02)
56	200[M+](91) 129(100) 157(88)	1556	ND	0.10 (0.02)	0.13 (0.04)	0.08 (0.01)	0.09 (0.02)	0.12 (0.03)	0.11 (0.02)
57	204[M+](8) 143(100) 157(98)	1559	ND	0.07 (0.01)	0.06 (0.01)	0.08 (0.01)	0.04 (0.01)	0.06 (0.04)	0.05 (0.01)
58	204[M+](82) 173(100) 189(94)	1563	ND	1.59 (0.03)	1.60 (0.02)	1.57 (0.02)	1.63 (0.02)	1.50 (0.03)	1.61 (0.02)
59	palustrol	1567 ^c^	1567 ^c^	9.90 (0.03)	9.94 (0.02)	9.78 (0.01)	9.82 (0.03)	9.86 (0.03)	9.88 (0.05)
60	200[M+](11) 79(100) 93(95)	1570	ND	0.54 (0.04)	0.51 (0.02)	0.49 (0.03)	0.47 (0.02)	0.53 (0.05)	0.56 (0.05)
61	204[M+](31) 81(100) 109(88)	1573	ND	0.91 (0.03)	0.93 (0.03)	0.87 (0.03)	0.90 (0.03)	0.88 (0.03)	0.94 (0.02)
62	spathulenol	1576 ^a,b,c^	1577 ^c^	5.11 (0.03)	5.05 (0.02)	4.91 (0.04)	4.93 (0.02)	4.98 (0.02)	4.99 (0.03)
63	200[M+](56) 185(100) 143(63)	1581	ND	5.61 (0.05)	5.37 (0.03)	5.29 (0.03)	5.54 (0.03)	5.59 (0.04)	5.64 (0.04)
64	202[M+](4) 91(100) 79(82)	1587	ND	0.47 (0.03)	0.46 (0.02)	0.51 (0.03)	0.49 (0.02)	0.47 (0.02)	0.48 (0.02)
65	globulol	1599 ^a,b,c,d^	1590 ^c^	3.11 (0.02)	3.14 (0.03)	3.03 (0.05)	2.99 (0.03)	3.07 (0.03)	2.94 (0.05)
66	200[M+](8) 198(100) 183(84)	1605	ND	0.21 (0.04)	0.26 (0.02)	0.23 (0.02)	0.22 (0.03)	0.36 (0.03)	0.31 (0.05)
67	220[M+](2) 145(100) 200(93)	1609	ND	2.21 (0.02)	2.19 (0.03)	2.17 (0.04)	2.11 (0.04)	2.16 (0.02)	2.03 (0.06)
68	(+)-bisabola-2,10-diene[1,9]oxide	1615 ^a,b^	1596 ^a^	0.16 (0.03)	0.17 (0.03)	0.14 (0.02)	0.19 (0.02)	0.21 (0.03)	0.22 (0.03)
69	208[M+](3) 95(100) 85(95)	1621	ND	1.33 (0.03)	1.54 (0.04)	1.47 (0.05)	1.23 (0.03)	1.42 (0.04)	1.37 (0.04)
70	ledene oxide-(II)	1631 ^a,b^	1631 ^a^	0.21 (0.02)	0.25 (0.02)	0.19 (0.03)	0.22 (0.04)	0.17 (0.03)	0.26 (0.03)
71	isospathulenol	1635 ^a,b^	1633 ^a^	0.91 (0.03)	0.89 (0.03)	0.93 (0.03)	0.98 (0.05)	0.94 (0.03)	0.88 (0.02)
72	220[M+](18) 91(100) 105(83)	1639	ND	1.59 (0.04)	1.63 (0.02)	1.51 (0.03)	1.58 (0.01)	1.53 (0.01)	1.62 (0.01)
73	cubenol	1642 ^a,b,c,d^	1645 ^c^	0.42 (0.03)	0.51 (0.03)	0.56 (0.04)	0.40 (0.02)	0.43 (0.03)	0.52 (0.01)
74	220[M+](21) 91(100) 105(82)	1651	ND	0.10 (0.03)	0.08 (0.02)	0.09 (0.01)	0.10 (0.01)	0.11 (0.02)	0.07 (0.02)
75	222[M+](3) 179(100) 121(92)	1655	ND	0.05 (0.01)	0.03 (0.01)	0.04 (0.01)	0.03 (0.01)	0.05 (0.01)	0.06 (0.01)
76	germacra-4(15),5,10(14)-trien-1-α-ol	1660 ^c^	1685 ^c^	0.89 (0.03)	0.96 (0.03)	1.03 (0.04)	0.99 (0.05)	0.94 (0.02)	0.86 (0.02)
77	216[M+](31) 145(100) 91(97)	1699	ND	0.68 (0.02)	0.58 (0.04)	0.53 (0.03)	0.56 (0.05)	0.69 (0.03)	0.61 (0.04)
78	1,4-dimethyl-7-(1-methylethyl)-azulene	1790 ^c^	1779 ^c^	3.43 (0.04)	3.52 (0.02)	3.50 (0.04)	3.49 (0.06)	3.27 (0.04)	3.33 (0.03)
79	14-hydroxy-δ-cadinene	1797 ^c^	1803 ^c^	0.36 (0.03)	0.42 (0.04)	0.40 (0.03)	0.38 (0.03)	0.36 (0.02)	0.41 (0.02)
	Total			98.30 (2.39)	98.36 (2.20)	97.19 (2.38)	97.35 (2.23)	98.24 (2.27)	98.26 (2.21)
	% identified			76.58 (1.42)	76.84 (1.31)	76.23 (1.42)	76.44 (1.29)	76.31 (1.22)	76.53 (1.30)
	including:								
	aliphatics			3.09 (0.25)	2.85 (0.23)	2.97 (0.29)	3.07 (0.21)	3.03 (0.20)	2.91 (0.20)
	aromatics			4.97 (0.18)	5.03 (0.18)	5.11 (0.20)	5.13 (0.17)	5.05 (0.15)	5.02 (0.14)
	monoterpene hydrocarbons			0.05 (0.01)	0.02 (0.01)	0.03 (0.01)	0.04 (0.01)	0.05 (0.01)	0.05 (0.01)
	monoterpenoid hydrocarbons			0.07 (0.01)	0.06 (0.01)	0.05 (0.01)	0.03 (0.01)	0.03 (0.01)	0.05 (0.01)
	sesquiterpene hydrocarbons			47.33 (0.72)	47.55 (0.63)	47.10 (0.62)	47.27 (0.60)	47.19 (0.61)	47.54 (0.68)
	sesquiterpenoid hydrocarbons			21.07 (0.25)	21.33 (0.25)	20.97 (0.29)	20.90 (0.29)	20.96 (0.24)	20.96 (0.26)

- less than 0.01%. * The names of terpenes and terpenoids according to IUPAC terminology are given in Table S5. ** Retention index on Quadex 007-5MS column. *** Literature retention index. ND, no data. **** For abbreviations of samples, see Table 1. Standard deviation in brackets. Identification of compounds by MS databases (^a^ NIST 2011, ^b^ NIST Chemistry WebBook, ^c^ Adams 4 Library, ^d^ Pherobase).

**Table 5:** Volatile compounds detected in the in vitro samples (CI-37–CI-38).

No.	Compounds *	RI **	RI ***	Code ****	
				CI-37	CI-38
1	propan-1-ol	<700 ^a,b^	483 ^a^	0.34 (0.03)	0.29 (0.04)
2	pentanal	705 ^a,b,c,d^	704 ^c^	1.76 (0.04)	1.57 (0.02)
3	hexanal	802 ^a,b,c,d^	801 ^c^	3.26 (0.02)	3.14 (0.02)
4	hexan-1-ol	867 ^a,b^	869 ^a^	0.33 (0.03)	0.37 (0.03)
5	heptanal	902 ^a,b,c,d^	901 ^c^	0.06 (0.01)	0.11 (0.02)
6	α-pinene	939 ^a,b,c^	932 ^c^	0.04 (0.01)	0.06 (0.02)
7	benzaldehyde	940 ^a,b,c^	952 ^c^	0.50 (0.02)	0.47 (0.03)
8	2-ethylhexan-1-ol	1023 ^a,b^	1025 ^a^	0.07 (0.01)	0.09 (0.02)
9	phenylmethanol	1028 ^a,b,c^	1026 ^c^	5.47 (0.04)	5.12 (0.04)
10	phenylacetaldehyde	1044 ^a,b^	1044 ^a^	0.23 (0.03)	0.27 (0.03)
11	nonanal	1102 ^a,b,c,d^	1100 ^c^	0.14 (0.02)	0.11 (0.02)
12	3,4-dimethylcyclohexan-1-ol	1115 ^a,b^	1126 ^a^	0.02 (0.01)	0.03 (0.01)
13	phenylethanol	1121 ^a,b^	1121 ^a^	0.15 (0.02)	0.18 (0.04)
14	decanal	1195 ^a,b,c,d^	1201 ^c^	0.14 (0.02)	0.15 (0.02)
15	β-cyclocitral	1221 ^c^	1217 ^c^	0.27 (0.03)	0.33 (0.03)
16	2-phenoxyethan-1-ol	1225 ^a,b^	1226 ^a^	4.61 (0.05)	4.47 (0.04)
17	bicycloelemene	1316 ^a^	1330 ^a^	0.02 (0.01)	0.02 (0.01)
18	δ-elemene	1324 ^a,b,c^	1335 ^c^	0.49 (0.03)	0.39 (0.06)
19	204[M+](5) 121(100) 93(89)	1343	ND	0.05 (0.01)	0.07 (0.02)
20	200[M+](39) 159(100) 117(95)	1345	ND	0.05 (0.01)	0.06 (0.01)
21	202[M+](13) 81(100) 96(73)	1350	ND	0.22 (0.02)	0.29 (0.03)
22	204[M+](10) 119(100) 91(84)	1353	ND	0.05 (0.01)	0.07 (0.01)
23	anastreptene	1370 ^a^	1370 ^a^	31.14 (0.06)	29.98 (0.05)
24	204[M+](5) 81(100) 93(96)	1384	ND	0.09 (0.01)	0.13 (0.02)
25	β-elemene	1391 ^a,b,c^	1389 ^c^	0.55 (0.03)	0.65 (0.04)
26	204[M+](13) 157(100) 185(84)	1398	ND	0.08 (0.01)	0.09 (0.01)
27	204[M+](13) 157(100) 185(84)	1417	ND	0.08 (0.01)	0.07 (0.01)
28	204[M+](19) 135(100) 105(82)	1423	ND	0.12 (0.02)	0.15 (0.02)
29	204[M+](9) 91(100) 105(93)	1425	ND	0.15 (0.01)	0.13 (0.02)
30	(-)-aristolene	1429 ^a,b,c,d^	1428 ^a^	0.75 (0.03)	0.82 (0.04)
31	204[M+](9) 107(100) 79(43)	1432	ND	0.30 (0.04)	0.34 (0.03)
32	γ-maaliene	1435 ^a,b^	1427 ^a^	0.38 (0.03)	0.41 (0.04)
33	α-maaliene	1443 ^a,b^	1442 ^a^	0.12 (0.02)	0.15 (0.05)
34	aromandendrene	1445 ^a,b^	1447 ^a^	5.04 (0.05)	5.12 (0.06)
35	selina-5,11-diene	1447 ^a,b^	1454 ^a^	0.22 (0.05)	0.29 (0.03)
36	dehydroaromadendrene	1456 ^c^	1460 ^c^	2.35 (0.02)	2.14 (0.03)
37	1,2,9,10-tetradehydroaristolane	1461	ND	0.91 (0.04)	1.01 (0.02)
38	204[M+](15) 91(100) 105(84)	1465	ND	0.40 (0.03)	0.45 (0.04)
39	204[M+](18) 128(100) 143(95)	1469	ND	0.06 (0.01)	0.09 (0.02)
40	γ-gurjunene	1474 ^c,d^	1475 ^c^	0.19 (0.04)	0.21 (0.05)
41	γ-muurolene	1477 ^c^	1478 ^c^	0.07 (0.01)	0.04 (0.01)
42	δ-selinene	1488 ^c^	1492 ^c^	2.63 (0.05)	2.47 (0.03)
43	ledene	1492 ^a,b,c^	1496 ^c^	1.88 (0.03)	1.93 (0.02)
44	204[M+](38) 105(100) 93(96)	1495	ND	0.18 (0.02)	0.22 (0.02)
45	bicyclogermacrene	1499 ^a,b,c^	1500 ^c^	12.91 (0.03)	13.01 (0.04)
46	204[M+](19) 93(100) 91(95)	1505	ND	0.11 (0.02)	0.15 (0.03)
47	202[M+](25) 133(100) 91(89)	1509	ND	0.03 (0.01)	0.05 (0.01)
48	206[M+](14) 191(100) 57(38)	1514	ND	0.22 (0.02)	0.27 (0.04)
49	202[M+](33) 131(100) 145(53)	1518	ND	0.15 (0.01)	0.12 (0.03)
50	δ-cadinene	1524 ^a,b,c^	1522 ^c^	0.04 (0.01)	0.06 (0.01)
51	204[M+](5) 91(100) 131(95)	1530	ND	0.03 (0.01)	0.05 (0.01)
52	200[M+](54) 185(100) 143(91)	1535	ND	-	-
53	4,5,9,10-dehydro-isolongifolene	1544 ^a,b^	1544 ^a^	2.00 (0.04)	2.12 (0.03)
54	202[M+](4) 128(100) 157(95)	1547	ND	0.15 (0.03)	0.21 (0.03)
55	200[M+](8) 171(100) 186(79)	1551	ND	0.10 (0.02)	0.13 (0.04)
56	200[M+](91) 129(100) 157(88)	1556	ND	0.09 (0.02)	0.11 (0.02)
57	204[M+](8) 143(100) 157(98)	1559	ND	0.23 (0.02)	0.18 (0.03)
58	204[M+](82) 173(100) 189(94)	1563	ND	0.97 (0.05)	1.01 (0.03)
59	palustrol	1567 ^c^	1567 ^c^	4.95 (0.05)	5.06 (0.04)
60	200[M+](11) 79(100) 93(95)	1570	ND	0.35 (0.04)	0.28 (0.04)
61	204[M+](31) 81(100) 109(88)	1573	ND	0.74 (0.03)	0.81 (0.04)
62	spathulenol	1576 ^a,b,c^	1577 ^c^	0.54 (0.05)	0.44 (0.03)
63	200[M+](56) 185(100) 143(63)	1581	ND	2.72 (0.03)	2.81 (0.03)
64	202[M+](4) 91(100) 79(82)	1587	ND	0.18 (0.02)	0.21 (0.03)
65	globulol	1599 ^a,b,c,d^	1590 ^c^	1.51 (0.04)	1.41 (0.04)
66	200[M+](8) 198(100) 183(84)	1605	ND	-	-
67	220[M+](2) 145(100) 200(93)	1609	ND	0.11 (0.01)	0.13 (0.04)
68	(+)-bisabola-2,10-diene[1,9]oxide	1615 ^a,b^	1596 ^a^	0.31 (0.04)	0.29 (0.03)
69	208[M+](3) 95(100) 85(95)	1621	ND	0.62 (0.03)	0.67 (0.02)
70	ledene oxide-(II)	1631 ^a,b^	1631 ^a^	-	-
71	isospathulenol	1635 ^a,b^	1633 ^a^	0.27 (0.02)	0.37 (0.02)
72	220[M+](18) 91(100) 105(83)	1639	ND	0.81 (0.03)	0.93 (0.04)
73	cubenol	1642 ^a,b,c,d^	1645 ^c^	0.05 (0.01)	0.03 (0.01)
74	220[M+](21) 91(100) 105(82)	1651	ND	0.06 (0.01)	0.09 (0.01)
75	222[M+](3) 179(100) 121(92)	1655	ND	0.05 (0.01)	0.07 (0.01)
76	germacra-4(15),5,10(14)-trien-1-α-ol	1660 ^c^	1685 ^c^	0.23 (0.05)	0.32 (0.03)
77	216[M+](31) 145(100) 91(97)	1699	ND	0.61 (0.05)	0.59 (0.03)
78	1,4-dimethyl-7-(1-methylethyl)-azulene	1790 ^c^	1779 ^c^	0.59 (0.03)	0.64 (0.04)
79	14-hydroxy-δ-cadinene	1797 ^c^	1803 ^c^	0.26 (0.03)	0.33 (0.02)
	Total			97.95 (1.97)	97.50 (2.13)
	% identified			87.79 (1.29)	86.47 (1.31)
	including:				
	aliphatics			6.12 (0.19)	5.86 (0.20)
	aromatics			10.96 (0.16)	10.51 (0.18)
	monoterpene hydrocarbons			0.04 (0.01)	0.06 (0.02)
	monoterpenoid hydrocarbons			0.27 (0.03)	0.33 (0.03)
	sesquiterpene hydrocarbons			62.28 (0.61)	61.46 (0.66)
	sesquiterpenoid hydrocarbons			8.12 (0.29)	8.25 (0.22)

- less than 0.01%. * The names of terpenes and terpenoids according to IUPAC terminology are given in Table S5. ** Retention index on Quadex 007-5MS column. *** Literature retention index. ND, no data. **** For abbreviations of samples, see Table 1. Standard deviation in brackets. Identification of compounds by MS databases (^a^ NIST 2011, ^b^ NIST Chemistry WebBook, ^c^ Adams 4 Library, ^d^ Pherobase).

**Table 6:** Values of literature retention index (RI **) and mean values calculated based on the results obtained for individual seasons and in vitro samples presented in Table 2, Table 3, Table 4 and Table 5.

No.	Compounds *	RI **	RI ***	Spring (May)	Summer (July)	Autumn (September)	In Vitro Culture
1	propan-1-ol	<700 ^a,b^	483 ^a^	1.10 (0.08)	0.40 (0.06)	1.35 (0.08)	0.32 (0.04)
2	pentanal	705 ^a,b,c,d^	704 ^c^	0.33 (0.05)	0.31 (0.03)	0.42 (0.03)	1.67 (0.13)
3	hexanal	802 ^a,b,c,d^	801 ^c^	0.20 (0.02)	0.07 (0.02)	0.41 (0.02)	3.20 (0.08)
4	hexan-1-ol	867 ^a,b^	869 ^a^	0.50 (0.04)	0.32 (0.02)	0.29 (0.03)	0.35 (0.03)
5	heptanal	902 ^a,b,c,d^	901 ^c^	0.05 (0.03)	0.04 (0.01)	0.03 (0.01)	0.09 (0.04)
6	α-pinene	939 ^a,b,c^	932 ^c^	0.03 (0.02)	0.02 (0.01)	0.04 (0.01)	0.05 (0.01)
7	benzaldehyde	940 ^a,b,c^	952 ^c^	0.20 (0.04)	0.09 (0.02)	0.48 (0.04)	0.49 (0.02)
8	2-ethylhexan-1-ol	1023 ^a,b^	1025 ^a^	0.08 (0.03)	0.04 (0.01)	0.21 (0.03)	0.08 (0.01)
9	phenylmethanol	1028 ^a,b,c^	1026 ^c^	1.11 (0.06)	1.03 (0.05)	1.03 (0.05)	5.30 (0.25)
10	phenylacetaldehyde	1044 ^a,b^	1044 ^a^	1.17 (0.05)	0.21 (0.02)	1.70 (0.04)	0.25 (0.03)
11	nonanal	1102 ^a,b,c,d^	1100 ^c^	0.10 (0.03)	0.02 (0.01)	0.11 (0.02)	0.13 (0.02)
12	3,4-dimethylcyclohexan-1-ol	1115 ^a,b^	1126 ^a^	0.10 (0.02)	0.03 (0.02)	0.10 (0.02)	0.03 (0.01)
13	phenylethanol	1121 ^a,b^	1121 ^a^	0.13 (0.02)	0.69 (0.04)	0.58 (0.03)	0.17 (0.02)
14	decanal	1195 ^a,b,c,d^	1201 ^c^	0.06 (0.02)	0.03 (0.02)	0.07 (0.02)	0.15 (0.01)
15	β-cyclocitral	1221 ^c^	1217 ^c^	0.07 (0.02)	0.04 (0.01)	0.05 (0.01)	0.30 (0.04)
16	2-phenoxyethan-1-ol	1225 ^a,b^	1226 ^a^	0.94 (0.05)	0.41 (0.02)	1.24 (0.08)	4.54 (0.10)
17	bicycloelemene	1316 ^a^	1330 ^a^	0.12 (0.03)	0.08 (0.01)	0.07 (0.02)	0.02 (0.00)
18	δ-elemene	1324 ^a,b,c^	1335 ^c^	0.76 (0.06)	1.98 (0.26)	0.71 (0.02)	0.44 (0.07)
19	204[M+](5) 121(100) 93(89)	1343	ND	0.19 (0.04)	0.42 (0.05)	0.13 (0.02)	0.06 (0.01)
20	200[M+](39) 159(100) 117(95)	1345	ND	1.05 (0.06)	0.72 (0.12)	1.28 (0.03)	0.06 (0.01)
21	202[M+](13) 81(100) 96(73)	1350	ND	0.12 (0.04)	0.20 (0.02)	0.12 (0.02)	0.26 (0.05)
22	204[M+](10) 119(100) 91(84)	1353	ND	0.06 (0.02)	0.07 (0.02)	0.10 (0.01)	0.06 (0.01)
23	anastreptene	1370 ^a^	1370 ^a^	18.07 (0.12)	25.26 (0.10)	15.61 (0.25)	30.56 (0.82)
24	204[M+](5) 81(100) 93(96)	1384	ND	0.20 (0.04)	0.16 (0.03)	0.18 (0.04)	0.11 (0.03)
25	β-elemene	1391 ^a,b,c^	1389 ^c^	2.77 (0.06)	2.21 (0.24)	1.29 (0.03)	0.60 (0.07)
26	204[M+](13) 157(100) 185(84)	1398	ND	0.28 (0.06)	0.22 (0.03)	0.23 (0.03)	0.09 (0.01)
27	204[M+](13) 157(100) 185(84)	1417	ND	0.28 (0.07)	0.25 (0.03)	0.34 (0.03)	0.08 (0.01)
28	204[M+](19) 135(100) 105(82)	1423	ND	0.21 (0.05)	0.30 (0.05)	0.16 (0.03)	0.14 (0.02)
29	204[M+](9) 91(100) 105(93)	1425	ND	0.04 (0.02)	0.08 (0.02)	0.04 (0.02)	0.14 (0.01)
30	(-)-aristolene	1429 ^a,b,d^	1428 ^a^	1.04 (0.07)	0.72 (0.05)	1.19 (0.04)	0.79 (0.05)
31	204[M+](9) 107(100) 79(43)	1432	ND	0.17 (0.04)	0.24 (0.04)	0.13 (0.02)	0.32 (0.03)
32	γ-maaliene	1435 ^a^	1427 ^a^	0.50 (0.03)	0.50 (0.09)	0.22 (0.04)	0.40 (0.02)
33	α-maaliene	1443 ^a^	1442 ^a^	0.32 (0.04)	0.41 (0.08)	0.25 (0.04)	0.14 (0.02)
34	aromandendrene	1445 ^a,b^	1447 ^a^	3.18 (0.10)	6.96 (0.74)	3.25 (0.08)	5.08 (0.06)
35	selina-5,11-diene	1447 ^a,b^	1454 ^a^	0.55 (0.08)	0.73 (0.09)	0.29 (0.03)	0.26 (0.05)
36	dehydroaromadendrene	1456 ^c^	1460 ^c^	1.14 (0.08)	1.49 (0.08)	1.16 (0.07)	2.25 (0.15)
37	1,2,9,10-tetradehydroaristolane	1461	ND	0.46 (0.04)	1.14 (0.13)	0.48 (0.04)	0.96 (0.07)
38	204[M+](15) 91(100) 105(84)	1465	ND	0.35 (0.07)	0.36 (0.06)	0.30 (0.06)	0.43 (0.04)
39	204[M+](18) 128(100) 143(95)	1469	ND	0.31 (0.04)	0.37 (0.05)	0.36 (0.03)	0.08 (0.02)
40	γ-gurjunene	1474 ^c,d^	1475 ^c^	0.48 (0.05)	0.46 (0.05)	0.31(0.03)	0.20 (0.01)
41	γ-muurolene	1477 ^c^	1478 ^c^	0.16 (0.04)	0.22 (0.04)	0.11 (0.02)	0.06 (0.02)
42	δ-selinene	1488 ^c^	1492 ^c^	1.42 (0.12)	2.17 (0.07)	1.05 (0.06)	2.55 (0.11)
43	ledene	1492 ^a,b,c^	1496 ^c^	1.63 (0.06)	1.98 (0.09)	1.40 (0.14)	1.91 (0.04)
44	204[M+](38) 105(100) 93(96)	1495	ND	0.28 (0.08)	0.21 (0.05)	0.08 (0.02)	0.20 (0.03)
45	bicyclogermacrene	1499 ^a,b,c^	1500 ^c^	8.37 (0.09)	17.35 (0.60)	7.53 (0.29)	12.96 (0.07)
46	204[M+](19) 93(100) 91(95)	1505	ND	0.18 (0.05)	0.45 (0.14)	0.15 (0.03)	0.13 (0.03)
47	202[M+](25) 133(100) 91(89)	1509	ND	0.20 (0.04)	0.12 (0.04)	0.19 (0.04)	0.04 (0.01)
48	206[M+](14) 191(100) 57(38)	1514	ND	0.22 (0.04)	0.18 (0.09)	0.13 (0.03)	0.25 (0.04)
49	202[M+](33) 131(100) 145(53)	1518	ND	0.19 (0.03)	0.41 (0.05)	0.19 (0.02)	0.14 (0.02)
50	δ-cadinene	1524 ^a,b,c^	1522 ^c^	0.31 (0.04)	0.12 (0.03)	0.33 (0.04)	0.05 (0.01)
51	204[M+](5) 91(100) 131(95)	1530	ND	0.10 (0.03)	0.17 (0.03)	0.09 (0.03)	0.04 (0.01)
52	200[M+](54) 185(100) 143(91)	1535	ND	0.15 (0.03)	-	0.20 (0.03)	-
53	4,5,9,10-dehydro-isolongifolene	1544 ^a,b^	1544 ^a^	6.29 (0.12)	4.49 (0.24)	8.53 (0.21)	2.06 (0.08)
54	202[M+](4) 128(100) 157(95)	1547	ND	0.69 (0.04)	0.75 (0.06)	1.54 (0.08)	0.18 (0.04)
55	200[M+](8) 171(100) 186(79)	1551	ND	0.19 (0.05)	0.09 (0.02)	0.19 (0.02)	0.12 (0.02)
56	200[M+](91) 129(100) 157(88)	1556	ND	0.09 (0.04)	0.06 (0.02)	0.11 (0.02)	0.10 (0.01)
57	204[M+](8) 143(100) 157(98)	1559	ND	0.08 (0.03)	0.05 (0.02)	0.06 (0.02)	0.21 (0.04)
58	204[M+](82) 173(100) 189(94)	1563	ND	1.42 (0.06)	1.21 (0.02)	1.60 (0.04)	0.99 (0.03)
59	palustrol	1567 ^c^	1567 ^c^	8.31 (0.10)	5.33 (0.15)	9.86 (0.05)	5.01 (0.08)
60	200[M+](11) 79(100) 93(95)	1570	ND	0.71 (0.15)	0.52 (0.09)	0.51 (0.04)	0.32 (0.05)
61	204[M+](31) 81(100) 109(88)	1573	ND	2.03 (0.07)	2.02 (0.27)	0.90 (0.03)	0.78 (0.05)
62	spathulenol	1576 ^a,b,c^	1577 ^c^	7.70 (0.24)	2.65 (0.06)	5.00 (0.06)	0.49 (0.07)
63	200[M+](56) 185(100) 143(63)	1581	ND	3.61 (0.06)	2.48 (0.08)	5.48 (0.14)	2.77 (0.06)
64	202[M+](4) 91(100) 79(82)	1587	ND	0.49 (0.05)	0.31 (0.03)	0.48 (0.02)	0.20 (0.02)
65	globulol	1599 ^a,b,c,d^	1590 ^c^	3.05 (0.12)	1.37 (0.13)	3.04 (0.07)	1.46 (0.07)
66	200[M+](8) 198(100) 183(84)	1605	ND	0.23 (0.04)	-	0.27 (0.06)	-
67	220[M+](2) 145(100) 200(93)	1609	ND	1.20 (0.06)	0.09 (0.01)	2.13 (0.08)	0.12 (0.01)
68	(+)-bisabola-2,10-diene[1,9]oxide	1615 ^a,b^	1596 ^a^	0.17 (0.05)	0.30 (0.02)	0.18 (0.03)	0.30 (0.01)
69	208[M+](3) 95(100) 85(95)	1621	ND	0.75 (0.07)	0.56 (0.16)	1.35 (0.16)	0.65 (0.04)
70	ledene oxide-(II)	1631 ^a,b^	1631 ^a^	0.23 (0.03)	-	0.21 (0.04)	-
71	isospathulenol	1635 ^a,b^	1633 ^a^	0.57 (0.05)	0.35 (0.04)	0.93 (0.05)	0.32 (0.07)
72	220[M+](18) 91(100) 105(83)	1639	ND	1.87 (0.14)	0.74 (0.03)	1.58 (0.04)	0.87 (0.08)
73	cubenol	1642 ^a,b,c,d^	1645 ^c^	0.48 (0.06)	0.08 (0.02)	0.47 (0.06)	0.04 (0.01)
74	220[M+](21) 91(100) 105(82)	1651	ND	0.10 (0.02)	0.04 (0.01)	0.09 (0.01)	0.08 (0.02)
75	222[M+](3) 179(100) 121(92)	1655	ND	0.03 (0.02)	0.05 (0.01)	0.04 (0.01)	0.06 (0.01)
76	germacra-4(15),5,10(14)-trien-1-α-ol	1660 ^c^	1685 ^c^	0.59 (0.05)	0.89 (0.03)	0.95 (0.07)	0.28 (0.06)
77	216[M+](31) 145(100) 91(97)	1699	ND	0.43 (0.04)	0.20 (0.04)	0.60 (0.06)	0.60 (0.01)
78	1,4-dimethyl-7-(1-methylethyl)-azulene	1790 ^c^	1779 ^c^	2.70 (0.06)	1.22 (0.10)	3.35 (0.17)	0.62 (0.04)
79	14-hydroxy-δ-cadinene	1797 ^c^	1803 ^c^	0.34 (0.06)	0.12 (0.05)	0.39 (0.02)	0.30 (0.05)
	Total			96.38 (4.42)	98.43 (5.75)	97.60 (3.96)	97.91 (3.90)
	% identified			77.88 (2.63)	84.33 (3.96)	76.27 (2.59)	87.23 (3.02)
	including:						
	aliphatics			2.52 (0.32)	1.26 (0.20)	2.99 (0.26)	6.02 (0.37)
	aromatics			3.55 (0.22)	2.43 (0.15)	5.03 (0.24)	10.75 (0.42)
	monoterpene hydrocarbons			0.03 (0.02)	0.02 (0.01)	0.04 (0.01)	0.05 (0.01)
	monoterpenoid hydrocarbons			0.07 (0.02)	0.04 (0.01)	0.05 (0.01)	0.30 (0.01)
	sesquiterpene hydrocarbons			50.27 (1.29)	69.49 (3.09)	47.13 (1.62)	61.91 (1.76)
	sesquiterpenoid hydrocarbons			21.44 (0.76)	11.09 (0.50)	21.03 (0.45)	8.20 (0.42)

- less than 0.01%. * The names of terpenes and terpenoids according to IUPAC terminology are given in Table S5. ** Retention index on Quadex 007-5MS column. *** Literature retention index. ND, no data. Standard deviation in brackets. Identification of compounds by MS databases (^a^ NIST 2011, ^b^ NIST Chemistry WebBook, ^c^ Adams 4 Library, ^d^ Pherobase).

**Table 7:** Ranges of variability of the main compounds indicated by PCA in *Calypogeia integristipula* samples across seasons.

No.	Compounds	Spring	Summer	Autumn	In Vitro
Highest loading (≥95%) in PC1					
1	2-propanol	0.95–1.19	0.29–0.48	1.21–1.45	0.29–0.34
10	benzeneacetaldehyde	1.09–1.24	0.18–0.25	1.63–1.75	0.23–0.27
23	anastreptene	17.87–18.23	25.06–25.41	15.27–16.01	29.98–31.14
30	(-)-aristolene	0.91–1.18	0.63–0.80	1.11–1.25	0.75–0.82
42	δ-selinene	1.24–1.58	2.01–2.25	0.96–1.14	2.47–2.63
45	bicyclogermacrene	8.24–8.51	16.27–18.09	6.99–7.87	12.91–13.01
52	200[M+](54) 185(100) 143(91)	0.10–0.21	0.00–0.00	0.16–0.25	0.00–0.00
59	palustrol	8.14–8.46	5.09–5.58	9.78–9.94	4.95–5.06
65	globulol	2.87–3.32	1.07–1.52	2.94–3.15	1.41–1.51
67	220[M+](2) 145(100) 200(93)	1.11–1.31	0.06–0.11	1.99–2.22	0.11–0.13
78	1,4-dimethyl-7-(1-methylethyl)-azulene	2.59–2.77	1.06–1.33	3.02–3.56	0.59–0.64
Highest loading (≥75%) in PC2					
2	pentanal	0.25–0.39	0.26–0.35	0.36–0.47	1.57–1.76
3	hexanal	0.16–0.23	0.04–0.09	0.38–0.45	3.14–3.26
9	benzenemethanol	1.03–1.21	0.96–1.11	0.97–1.11	5.12–5.47
16	phenoxyethanol	0.87–1.01	0.37–0.45	1.11–1.35	4.47–4.61
39	204[M+](18) 128(100) 143(95)	0.26–0.37	0.25–0.44	0.29–0.40	0.06–0.09
57	204[M+](8) 143(100) 157(98)	0.04–0.12	0.02–0.07	0.03–0.08	0.18–0.23
Highest loading (≥85%) in PC3					
4	1-hexanol	0.41–0.56	0.29–0.35	0.25–0.33	0.33–0.37
13	benzeneethanol	0.09–0.16	0.63–0.75	0.54–0.62	0.15–0.18
76	germacra-4(15),5,10(14)-trien-1-α-ol	0.47–0.65	0.83–0.95	0.83–1.03	0.23–0.32

## Data Availability

The data presented in this study are available on request from the corresponding author.

## Supplementary Materials

The following supporting information can be downloaded at: https://www.mdpi.com/article/10.3390/molecules28217276/s1, Figure S1. Line plot of the principal component PC1 for the examined samples of *Calypogeia integristipula* based on all 79 detected compounds. The red lines represent +/−3.00 standard deviations. SD: 6.315; Figure S2. Line plot of the principal component PC2 for the examined samples of *Calypogeia integristipula* based on all 79 detected compounds. The red lines represent +/−3.00 standard deviations. SD: 3.832; Figure S3. Line plot of the principal component PC3 for the examined samples of *Calypogeia integristipula* based on all 79 detected compounds. The red lines represent +/−3.00 standard deviations. SD: 3.199; Figure S4. Linear plot of the lodgings for the first principal component PC1; Figure S5. Linear plot of the lodgings for the second principal component PC2; Figure S6. Linear plot of the lodgings for the third principal component PC3; Table S1. The *Calypogeia integristipula* sampling data in 2021 used for studies divided into collecting season; Table S2a. Volatile compounds detected in the samples collected in spring (CI-39–CI-44); Table S2b. Volatile compounds detected in the samples collected in spring (CI-45–CI-50); Table S3a. Volatile compounds detected in the samples collected in summer (CI-51–CI-56); Table S3b. Volatile compounds detected in the samples collected in summer (CI-56–CI-62); Table S4a. Volatile compounds detected in the samples collected in autumn (CI-63–CI-68); Table S4b. Volatile compounds detected in the samples collected in autumn (CI-69–CI-74); Table S5. IUPAC name for detected terpenes and terpenoids; Figure S7. Three-dimensional PCA scatter plot based on all 79 detected compounds in samples of *Calypogeia integristipula* collected in spring, summer, and autumn in 2022 and 2021 and in vitro. The percentage of explained variance (R2X) is 52.5% for PC1, 14.8% for PC2, 12.8% for PC3, and predictive ability (Q2) is 48.2%, 16.7%, and 25.2%, respectively; Figure S8. Line plot of the component PC1 for the examined samples of *Calypogeia integristipula* collected in 2021 and 2022 based on all 79 detected compounds. The red lines represent +/−3.00 standard deviations. SD: 6.637 (Figure S7); Figure S9. Line plot of the component PC2 for the examined samples of *Calypogeia integristipula* collected in 2021 and 2022 based on all 79 detected compounds. The red lines represent +/−3.00 standard deviations. SD: 3.416 (Figure S7); Figure S10. Line plot of the component PC3 for the examined samples of *Calypogeia integristipula* collected in 2021 and 2022 based on all 79 detected compounds. The red lines represent +/−3.00 standard deviations. SD: 3.182 (Figure S7); Figure S11. Linear plot of the lodgings for the first principal component PC1 (Figure S7); Figure S12. Linear plot of the lodgings for the second principal component PC2 (Figure S7); Figure S13. Linear plot of the lodgings for the third principal component PC3 (Figure S7).
