# Supplementary material for: Seasonal Variability of Volatile Components in Calypogeia integristipula
Source: Molecules. 2023 Oct 26;28(21):7276. doi: 10.3390/molecules28217276 (PMC10649560; doi:10.3390/molecules28217276)
Supplement: Supplementary file 1 [file molecules-28-07276-s001.zip › molecules-2618251-supplementary.pdf]

molecules

MDPI

Supplementary Material

Seasonal Variability on Volatile Components in *Calypogeia integristipula*

Rafał Wawrzyniak [1,*], Małgorzata Guzowska [1], Wiesław Wasiak [1], Beata Jasiewicz [1], Alina Bączkiewicz [2] and Katarzyna Buczkowska [2]

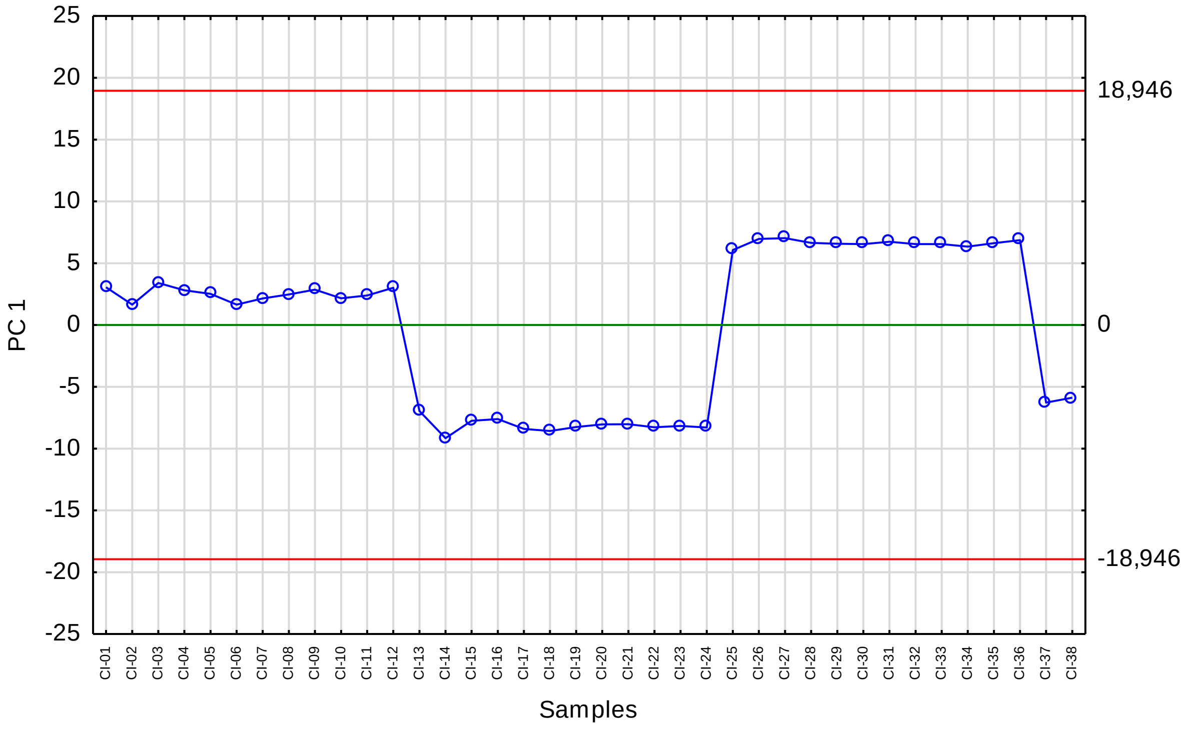

Figure S1. Line plot of the principal component PC1 for the examined samples of *Calypogeia integristipula* based on all 79 detected compounds. The red lines represent +/-3.00 standard deviations. SD: 6.315.

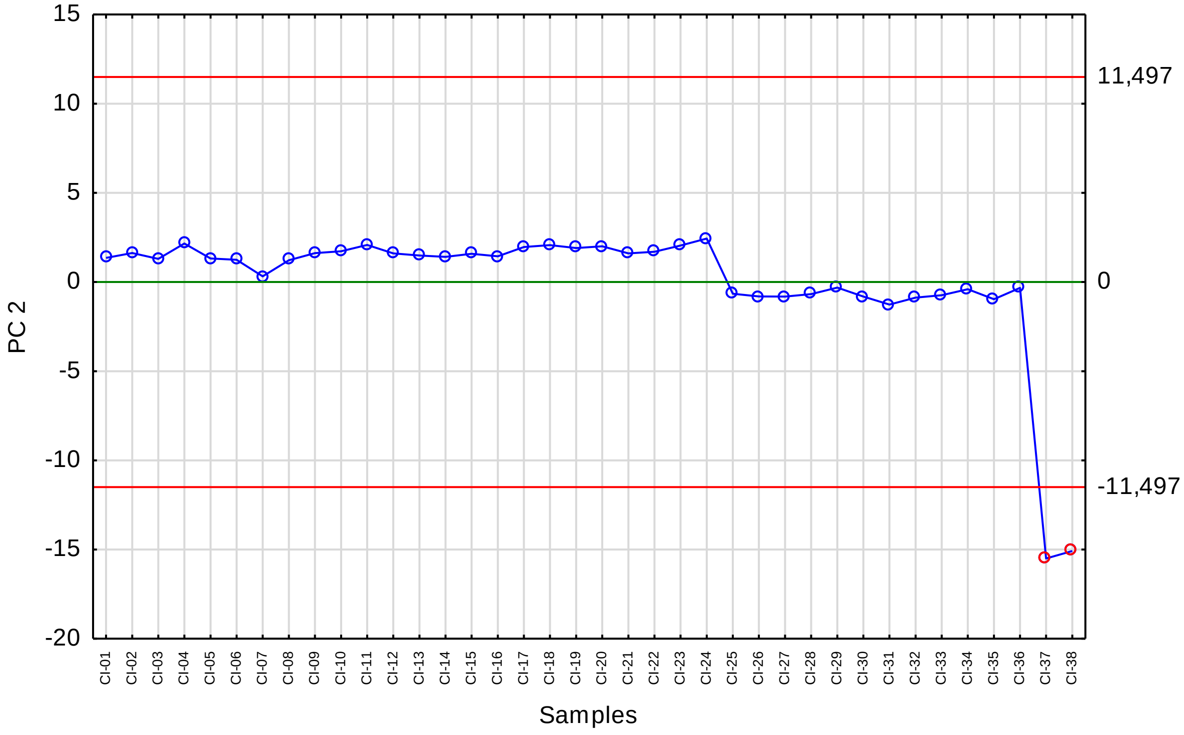

Figure S2. Line plot of the principal component PC2 for the examined samples of *Calypogeia integristipula* based on all 79 detected compounds. The red lines represent +/-3.00 standard deviations. SD: 3.832.

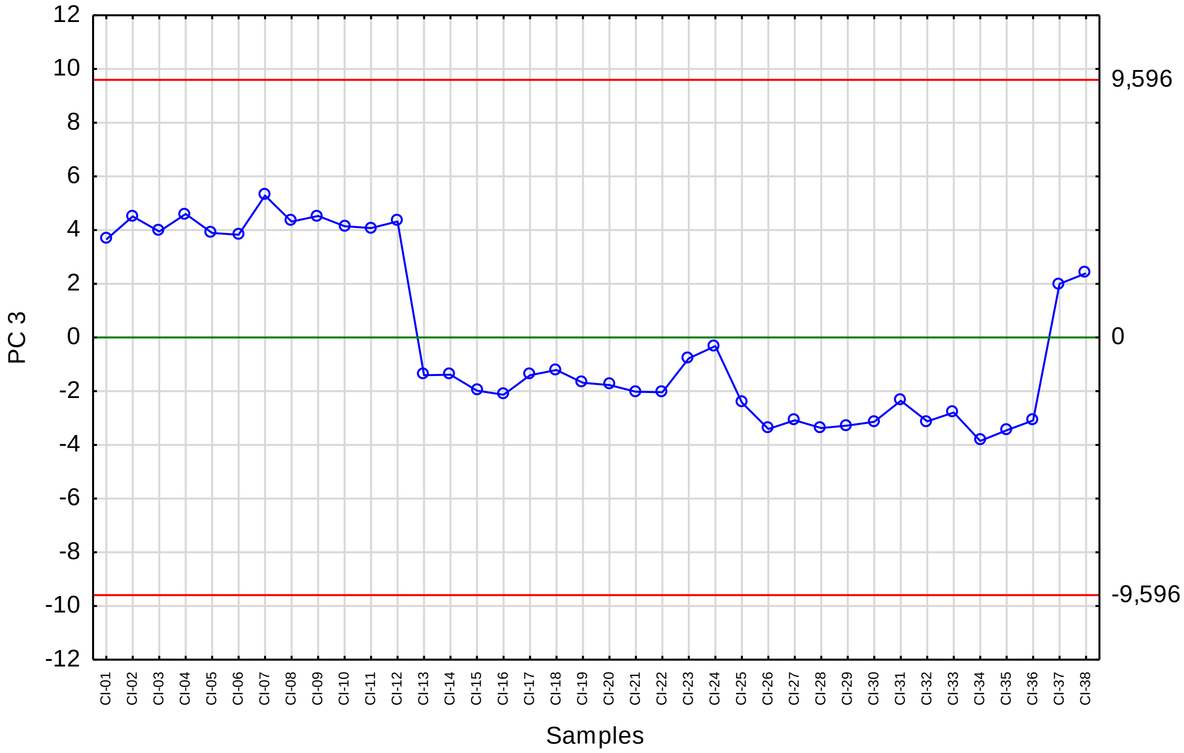

Figure S3. Line plot of the principal component PC3 for the examined samples of *Calypogeia integristipula* based on all 79 detected compounds. The red lines represent +/-3.00 standard deviations. SD: 3.199.

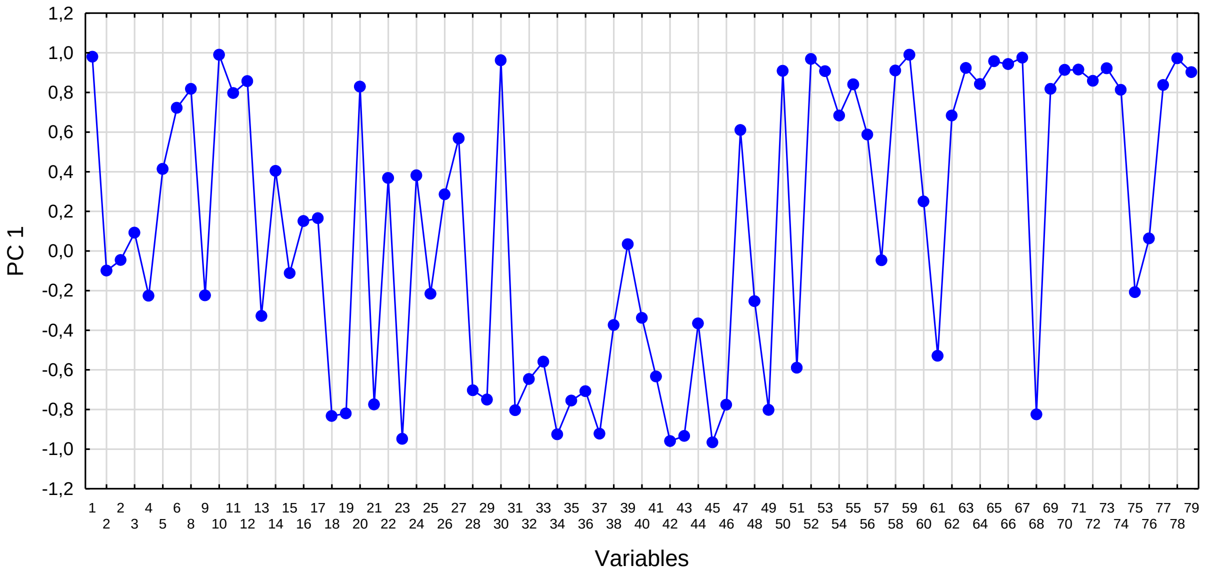

Figure S4. Linear plot of the lodgings for the first principal component PC1.

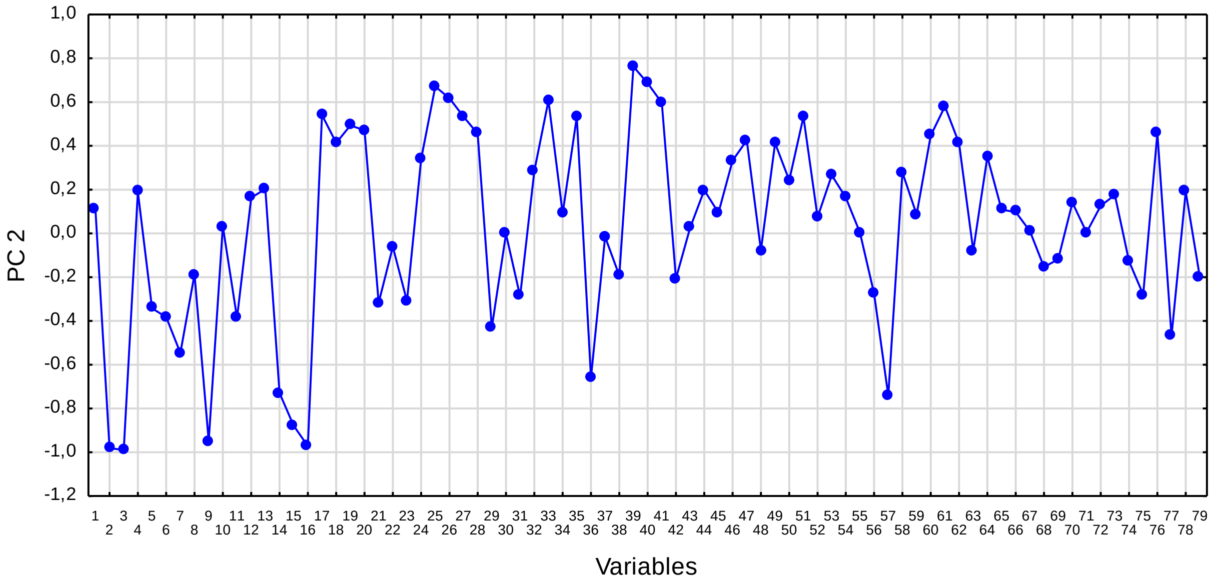

Figure S5. Linear plot of the lodgings for the second principal component PC2.

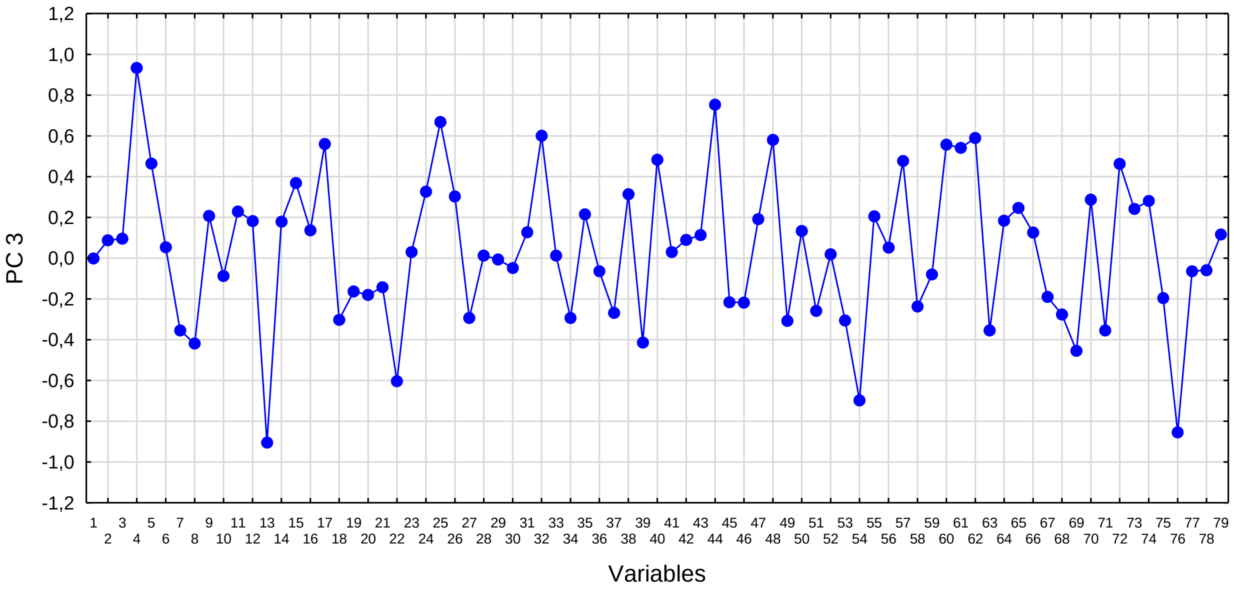

Figure S6. Linear plot of the lodgings for the third principal component PC3.

Table S1. The *Calypogeia integristipula* sampling data in 2021 year used for studies divided into collecting season.

Sample Code	Collection Place	Geographical Coordinates	Date month year
	**Spring**		
CI-39	South-Eastern Poland, Bieszczady Mts, Rozsypaniec	49°03'35.5"N, 22°46'10.7"E	05.2021
CI-40	Southern Poland, Tatry Mts, Morskie Oko	49°12'00.0"N, 20°04'24.3"E	05.2021
CI-41	Southern Poland, Tatry Mts, Gąsienicowa Valley	49°14'45.8"N, 20°00'25.1"E	05.2021
CI-42	Southern Poland, Tatry Mts, Kościeliska Valley	49°15'18.3"N, 19°51'55.4"E	05.2021
CI-43	Southern Poland, Małe Pieniny Mts	49°22'56.8"N, 20°33'18.9"E	05.2021
CI-44	Southern Poland, Pieniny Mts, Potok Kotłowy stream	49°24'22.5"N, 20°24'02.1"E	05.2021
CI-45	Southern Poland, Gorce Mts, Ochotnica Dolna	49°32'04.5"N, 20°19'00.0"E	05.2021
CI-46	Central Poland, Wielkopolska, Antonin	51°30'59.8"N, 17°50'58.1"E	05.2021
CI-47	Western Poland, Lubuskie, Nabłoto	51°47'31.4"N, 14°46'55.8"E	05.2021
CI-48	North-Eastern Poland, Suwałki Lake District, Lake Łempis	54°02'38.2"N, 23°28'10.8"E	05.2021
CI-49	North-Western Poland, Pomerania, Lake Czarne	54°22'50.7"N, 18°12'07.6"E	05.2021
CI-50	North-Western Poland, Pomerania, Lake Lubygość	54°24'46.7"N, 17°58'44.0"E	05.2021
	**Summer**		
CI-51	South-Eastern Poland, Bieszczady Mts, Rozsypaniec	49°03'35.5"N, 22°46'10.7"E	07.2021
CI-52	Southern Poland, Tatry Mts, Morskie Oko	49°12'00.0"N, 20°04'24.3"E	07.2021
CI-53	Southern Poland, Tatry Mts, Gąsienicowa Valley	49°14'45.8"N, 20°00'25.1"E	07.2021
CI-54	Southern Poland, Tatry Mts, Kościeliska Valley	49°15'18.3"N, 19°51'55.4"E	07.2021
CI-55	Southern Poland, Małe Pieniny Mts	49°22'56.8"N, 20°33'18.9"E	07.2021
CI-56	Southern Poland, Pieniny Mts, Potok Kotłowy stream	49°24'22.5"N, 20°24'02.1"E	07.2021
CI-57	Southern Poland, Gorce Mts, Ochotnica Dolna	49°32'04.5"N, 20°19'00.0"E	07.2021
CI-58	Central Poland, Wielkopolska, Antonin	51°30'59.8"N, 17°50'58.1"E	07.2021
CI-59	Western Poland, Lubuskie, Nabłoto	51°47'31.4"N, 14°46'55.8"E	07.2021
CI-60	North-Eastern Poland, Suwałki Lake District, Lake Łempis	54°02'38.2"N, 23°28'10.8"E	07.2021
CI-61	North-Western Poland, Pomerania, Lake Czarne	54°22'50.7"N, 18°12'07.6"E	07.2021
CI-62	North-Western Poland, Pomerania, Lake Lubygość	54°24'46.7"N, 17°58'44.0"E	07.2021
	**Autumn**		
CI-63	South-Eastern Poland, Bieszczady Mts, Rozsypaniec	49°03'35.5"N, 22°46'10.7"E	09.2021
CI-64	Southern Poland, Tatry Mts, Morskie Oko	49°12'00.0"N, 20°04'24.3"E	09.2021
CI-65	Southern Poland, Tatry Mts, Gąsienicowa Valley	49°14'45.8"N, 20°00'25.1"E	09.2021
CI-66	Southern Poland, Tatry Mts, Kościeliska Valley	49°15'18.3"N, 19°51'55.4"E	09.2021
CI-67	Southern Poland, Małe Pieniny Mts	49°22'56.8"N, 20°33'18.9"E	09.2021
CI-68	Southern Poland, Pieniny Mts, Potok Kotłowy stream	49°24'22.5"N, 20°24'02.1"E	09.2021
CI-69	Southern Poland, Gorce Mts, Ochotnica Dolna	49°32'04.5"N, 20°19'00.0"E	09.2021
CI-70	Central Poland, Wielkopolska, Antonin	51°30'59.8"N, 17°50'58.1"E	09.2021
CI-71	Western Poland, Lubuskie, Nabłoto	51°47'31.4"N, 14°46'55.8"E	09.2021
CI-72	North-Eastern Poland, Suwałki Lake District, Lake Łempis	54°02'38.2"N, 23°28'10.8"E	09.2021
CI-73	North-Western Poland, Pomerania, Lake Czarne	54°22'50.7"N, 18°12'07.6"E	09.2021
CI-74	North-Western Poland, Pomerania, Lake Lubygość	54°24'46.7"N, 17°58'44.0"E	09.2021

Table S2a. Volatile compounds detected in the samples collected in spring (CI-39 – CI-44).

No.	Compounds*	RI**	RI***	Code****					
				CI-39	CI-40	CI-41	CI-42	CI-43	CI-44
1	propan-1-ol	<700[a,b]	483[a]	1.09 (0.03)	1.12 (0.02)	1.15 (0.01)	1.12 (0.02)	1.01 (0.03)	0.98 (0.03)
2	pentanal	705[a,b,c,d]	704[c]	0.41 (0.02)	0.35 (0.04)	0.41 (0.02)	0.36 (0.03)	0.22 (0.02)	0.33 (0.02)
3	hexanal	802[a,b,c,d]	801[c]	0.21 (0.04)	0.24 (0.03)	0.19 (0.03)	0.23 (0.01)	0.25 (0.02)	0.21 (0.02)
4	hexan-1-ol	867[a,b]	869[a]	0.47 (0.06)	0.51 (0.06)	0.47 (0.03)	0.47 (0.03)	0.45 (0.03)	0.39 (0.03)
5	heptanal	902[a,b,c,d]	901[c]	0.07 (0.02)	0.08 (0.01)	0.03 (0.02)	0.04 (0.02)	0.07 (0.01)	0.11 (0.01)
6	α-pinene	939[a,b,c]	932[c]	0.03 (0.01)	0.02 (0.01)	0.02 (0.01)	0.06 (0.01)	0.02 (0.01)	0.03 (0.01)
7	benzaldehyde	940[a,b,c]	952[c]	0.21 (0.02)	0.18 (0.05)	0.13 (0.02)	0.23 (0.02)	0.18 (0.02)	0.15 (0.02)
8	2-ethylhexan-1-ol	1023[a,b]	1025[a]	0.07 (0.03)	0.10 (0.01)	0.04 (0.02)	0.05 (0.03)	0.12 (0.03)	0.12 (0.03)
9	phenylmethanol	1028[a,b,c]	1026[c]	1.13 (0.02)	1.15 (0.05)	1.23 (0.03)	1.09 (0.03)	1.15 (0.02)	1.11 (0.04)
10	phenylacetaldehyde	1044[a,b]	1044[a]	1.15 (0.03)	1.22 (0.02)	1.13 (0.02)	1.09 (0.04)	1.21 (0.05)	1.09 (0.04)
11	nonanal	1102[a,b,c,d]	1100[c]	0.13 (0.02)	0.09 (0.04)	0.12 (0.04)	0.10 (0.01)	0.10 (0.02)	0.13 (0.02)
12	3,4-dimethylcyclohexan-1-ol	1115[a,b]	1126[a]	0.10 (0.02)	0.07 (0.05)	0.10 (0.02)	0.08 (0.01)	0.10 (0.01)	0.06 (0.03)
13	phenylethanol	1121[a,b]	1121[a]	0.13 (0.02)	0.12 (0.05)	0.11 (0.01)	0.14 (0.02)	0.12 (0.03)	0.15 (0.03)
14	decanal	1195[a,b,c,d]	1201[c]	0.06 (0.01)	0.04 (0.02)	0.02 (0.01)	0.06 (0.01)	0.05 (0.02)	0.05 (0.01)
15	β-cyclocitral	1221[c]	1217[c]	0.09 (0.02)	0.06 (0.01)	0.06 (0.03)	0.08 (0.01)	0.06 (0.01)	0.09 (0.02)
16	2-phenoxyethan-1-ol	1225[a,b]	1226[a]	0.97 (0.03)	0.78 (0.03)	1.01 (0.02)	0.98 (0.02)	0.95 (0.04)	0.92 (0.04)
17	bicycloelemene	1316[a]	1330[a]	0.15 (0.02)	0.10 (0.02)	0.09 (0.01)	0.15 (0.03)	0.12 (0.02)	0.11 (0.02)
18	δ-elemene	1324[a,b,c]	1335[c]	0.73 (0.03)	0.75 (0.03)	0.69 (0.02)	0.82 (0.04)	0.83 (0.03)	0.85 (0.03)
19	204[M+](5) 121(100) 93(89)	1343	ND	0.19 (0.02)	0.21 (0.02)	0.16 (0.03)	0.22 (0.03)	0.21 (0.02)	0.25 (0.03)
20	200[M+](39) 159(100) 117(95)	1345	ND	1.11 (0.06)	1.08 (0.04)	0.98 (0.04)	1.01 (0.05)	1.13 (0.04)	1.06 (0.04)
21	202[M+](13) 81(100) 96(73)	1350	ND	0.12 (0.01)	0.10 (0.02)	0.13 (0.01)	0.15 (0.01)	0.09 (0.01)	0.07 (0.01)
22	204[M+](10) 119(100) 91(84)	1353	ND	0.09 (0.01)	0.06 (0.01)	0.03 (0.02)	0.02 (0.01)	0.10 (0.02)	0.05 (0.01)
23	anastreptene	1370[a]	1370[a]	18.01 (0.05)	18.06 (0.04)	17.92 (0.04)	17.99 (0.06)	18.21 (0.05)	18.05 (0.06)
24	204[M+](5) 81(100) 93(96)	1384	ND	0.23 (0.01)	0.16 (0.02)	0.16 (0.03)	0.21 (0.02)	0.21 (0.01)	0.16 (0.04)
25	β-elemene	1391[a,b,c]	1389[c]	2.73 (0.02)	2.79 (0.03)	2.74 (0.04)	2.69 (0.03)	2.85 (0.04)	2.82 (0.03)
26	204[M+](13) 157(100) 185(84)	1398	ND	0.33 (0.01)	0.26 (0.04)	0.19 (0.03)	0.31 (0.01)	0.33 (0.03)	0.34 (0.01)
27	204[M+](13) 157(100) 185(84)	1417	ND	0.37 (0.02)	0.18 (0.02)	0.25 (0.02)	0.35 (0.02)	0.19 (0.01)	0.25 (0.04)
28	204[M+](19) 135(100) 105(82)	1423	ND	0.14 (0.01)	0.27 (0.03)	0.16 (0.04)	0.32 (0.02)	0.21 (0.03)	0.14 (0.01)
29	204[M+](9) 91(100) 105(93)	1425	ND	0.04 (0.01)	0.07 (0.02)	0.02 (0.01)	0.01 (0.01)	0.05 (0.01)	0.08 (0.02)
30	(-)-aristolene	1429[a,b,c,d]	1428[a]	1.05 (0.03)	1.06 (0.04)	0.97 (0.03)	1.02 (0.05)	1.15 (0.02)	1.08 (0.03)
31	204[M+](9) 107(100) 79(43)	1432	ND	0.16 (0.02)	0.20 (0.03)	0.15 (0.02)	0.15 (0.02)	0.21 (0.02)	0.19 (0.04)
32	γ-maaliene	1435[a,b]	1427[a]	0.51 (0.03)	0.51 (0.01)	0.48 (0.04)	0.47 (0.03)	0.48 (0.01)	0.45 (0.04)
33	α-maaliene	1443[a,b]	1442[a]	0.36 (0.03)	0.35 (0.03)	0.33 (0.04)	0.31 (0.02)	0.29 (0.03)	0.38 (0.01)
34	aromandendrene	1445[a,b]	1447[a]	3.33 (0.05)	3.12 (0.04)	3.22 (0.03)	3.24 (0.04)	3.05 (0.04)	3.11 (0.04)
35	selina-5,11-diene	1447[a,b]	1454[a]	0.42 (0.01)	0.61 (0.02)	0.44 (0.02)	0.41 (0.02)	0.62 (0.03)	0.64 (0.04)
36	dehydroaromadendrene	1456[c]	1460[c]	1.12 (0.05)	1.12 (0.03)	1.03 (0.03)	0.98 (0.05)	1.18 (0.04)	1.25 (0.05)
37	1,2,9,10-tetradehydroaristolane	1461	ND	0.45 (0.01)	0.41 (0.04)	0.39 (0.04)	0.41 (0.03)	0.47 (0.01)	0.48 (0.03)
38	204[M+](15) 91(100) 105(84)	1465	ND	0.36 (0.03)	0.29 (0.05)	0.29 (0.03)	0.29 (0.02)	0.45 (0.02)	0.53 (0.02)
39	204[M+](18) 128(100) 143(95)	1469	ND	0.34 (0.02)	0.35 (0.03)	0.33 (0.03)	0.33 (0.03)	0.33 (0.03)	0.36 (0.04)
40	γ-gurjunene	1474[c,d]	1475[c]	0.51 (0.03)	0.51 (0.03)	0.41 (0.04)	0.43 (0.02)	0.53 (0.02)	0.57 (0.02)
41	γ-muurolene	1477[c]	1478[c]	0.12 (0.01)	0.18 (0.02)	0.15 (0.03)	0.19 (0.01)	0.21 (0.01)	0.23 (0.05)
42	δ-selinene	1488[c]	1492[c]	1.39 (0.04)	1.55 (0.03)	1.32 (0.04)	1.42 (0.05)	1.42 (0.03)	1.31 (0.03)
43	ledene	1492[a,b,c]	1496[c]	1.48 (0.03)	1.63 (0.04)	1.59 (0.03)	1.62 (0.05)	1.54 (0.04)	1.82 (0.03)
44	204[M+](38) 105(100) 93(96)	1495	ND	0.16 (0.01)	0.32 (0.06)	0.16 (0.02)	0.39 (0.03)	0.18 (0.02)	0.32 (0.02)
45	bicyclogermacrene	1499[a,b,c]	1500[c]	8.34 (0.03)	8.41 (0.05)	8.23 (0.05)	8.34 (0.05)	8.31 (0.04)	8.62 (0.05)
46	204[M+](19) 93(100) 91(95)	1505	ND	0.11 (0.02)	0.21 (0.02)	0.15 (0.01)	0.21 (0.01)	0.19 (0.02)	0.19 (0.03)
47	202[M+](25) 133(100) 91(89)	1509	ND	0.23 (0.02)	0.17 (0.01)	0.21 (0.03)	0.14 (0.02)	0.25 (0.01)	0.22 (0.02)
48	206[M+](14) 191(100) 57(38)	1514	ND	0.19 (0.01)	0.22 (0.02)	0.24 (0.03)	0.16 (0.03)	0.31 (0.03)	0.21 (0.03)
49	202[M+](33) 131(100) 145(53)	1518	ND	0.21 (0.03)	0.14 (0.04)	0.13 (0.02)	0.22 (0.02)	0.21 (0.02)	0.18 (0.01)
50	δ-cadinene	1524[a,b,c]	1522[c]	0.34 (0.02)	0.32 (0.06)	0.29 (0.03)	0.34 (0.02)	0.28 (0.04)	0.32 (0.04)
51	204[M+](5) 91(100) 131(95)	1530	ND	0.07 (0.01)	0.09 (0.02)	0.10 (0.01)	0.12 (0.02)	0.11 (0.03)	0.11 (0.03)
52	200[M+](54) 185(100) 143(91)	1535	ND	0.19 (0.04)	0.15 (0.03)	0.13 (0.04)	0.15 (0.01)	0.10 (0.03)	0.09 (0.02)
53	4,5,9,10-dehydro-isolongifolene	1544[a,b]	1544[a]	6.14 (0.03)	6.24 (0.02)	6.23 (0.05)	6.31 (0.05)	5.99 (0.05)	6.37 (0.05)
54	202[M+](4) 128(100) 157(95)	1547	ND	0.70 (0.02)	0.69 (0.03)	0.71 (0.04)	0.69 (0.03)	0.71 (0.04)	0.68 (0.03)

55	200[M+](8) 171(100) 186(79)	1551	ND	0.25 (0.03)	0.10 (0.01)	0.25 (0.03)	0.22 (0.02)	0.32 (0.03)	0.15 (0.03)
56	200[M+](91) 129(100) 157(88)	1556	ND	0.10 (0.01)	0.06 (0.02)	0.12 (0.02)	0.10 (0.01)	0.12 (0.02)	0.16 (0.02)
57	204[M+](8) 143(100) 157(98)	1559	ND	0.03 (0.01)	0.09 (0.01)	0.03 (0.01)	0.06 (0.01)	0.10 (0.01)	0.12 (0.03)
58	204[M+](82) 173(100) 189(94)	1563	ND	1.39 (0.03)	1.42 (0.03)	1.42 (0.04)	1.39 (0.03)	1.39 (0.04)	1.42 (0.04)
59	palustrol	1567[c]	1567[c]	8.24 (0.04)	8.17 (0.04)	8.35 (0.03)	8.22 (0.05)	8.13 (0.05)	8.14 (0.06)
60	200[M+](11) 79(100) 93(95)	1570	ND	0.48 (0.01)	0.83 (0.03)	0.79 (0.02)	0.72 (0.03)	0.71 (0.03)	0.53 (0.03)
61	204[M+](31) 81(100) 109(88)	1573	ND	2.13 (0.06)	2.06 (0.03)	2.21 (0.01)	1.92 (0.02)	1.98 (0.02)	1.91 (0.02)
62	spathulenol	1576[a,b,c]	1577[c]	7.87 (0.05)	7.88 (0.04)	7.89 (0.05)	7.45 (0.04)	7.86 (0.05)	7.45 (0.05)
63	200[M+](56) 185(100) 143(63)	1581	ND	3.66 (0.05)	3.62 (0.03)	3.26 (0.03)	3.71 (0.03)	3.62 (0.03)	3.63 (0.03)
64	202[M+](4) 91(100) 79(82)	1587	ND	0.47 (0.03)	0.52 (0.03)	0.57 (0.04)	0.55 (0.02)	0.41 (0.01)	0.49 (0.02)
65	globulol	1599[a,b,c,d]	1590[c]	3.16 (0.03)	3.06 (0.02)	3.33 (0.02)	2.95 (0.02)	3.03 (0.03)	3.10 (0.03)
66	200[M+](8) 198(100) 183(84)	1605	ND	0.18 (0.02)	0.18 (0.03)	0.22 (0.03)	0.21 (0.04)	0.22 (0.02)	0.20 (0.02)
67	220[M+](2) 145(100) 200(93)	1609	ND	1.23 (0.02)	1.15 (0.03)	1.21 (0.02)	1.12 (0.04)	1.12 (0.02)	1.21 (0.05)
68	(+)-bisabola-2,10-diene[1,9]oxide	1615[a,b]	1596[a]	0.24 (0.01)	0.12 (0.01)	0.16 (0.02)	0.09 (0.02)	0.21 (0.02)	0.22 (0.04)
69	208[M+](3) 95(100) 85(95)	1621	ND	0.79 (0.05)	0.63 (0.02)	0.92 (0.04)	0.76 (0.05)	0.71 (0.03)	0.83 (0.03)
70	ledene oxide-(II)	1631[a,b]	1631[a]	0.21 (0.02)	0.23 (0.02)	0.33 (0.02)	0.21 (0.03)	0.23 (0.01)	0.19 (0.04)
71	isospathulenol	1635[a,b]	1633[a]	0.63 (0.01)	0.55 (0.03)	0.58 (0.01)	0.65 (0.03)	0.62 (0.03)	0.56 (0.02)
72	220[M+](18) 91(100) 105(83)	1639	ND	1.62 (0.03)	1.92 (0.04)	2.01 (0.02)	1.91 (0.04)	1.93 (0.03)	2.01 (0.03)
73	cubenol	1642[a,b,c,d]	1645[c]	0.47 (0.01)	0.46 (0.02)	0.55 (0.03)	0.57 (0.02)	0.48 (0.02)	0.56 (0.04)
74	220[M+](21) 91(100) 105(82)	1651	ND	0.11 (0.01)	0.08 (0.01)	0.12 (0.02)	0.13 (0.01)	0.12 (0.01)	0.08 (0.03)
75	222[M+](3) 179(100) 121(92)	1655	ND	0.06 (0.02)	0.02 (0.01)	0.02 (0.01)	0.05 (0.01)	0.06 (0.01)	0.02 (0.01)
76	germacra-4(15),5,10(14)-trien-1-α-ol	1660[c]	1685[c]	0.54 (0.03)	0.59 (0.03)	0.59 (0.02)	0.61 (0.02)	0.64 (0.03)	0.66 (0.05)
77	216[M+](31) 145(100) 91(97)	1699	ND	0.43 (0.02)	0.44 (0.04)	0.41 (0.03)	0.42 (0.03)	0.52 (0.02)	0.44 (0.04)
78	1,4-dimethyl-7-(1-methylethyl)-azulene	1790[c]	1779[c]	2.69 (0.06)	2.71 (0.05)	2.72 (0.02)	2.73 (0.04)	2.69 (0.04)	2.73 (0.03)
79	14-hydroxy-δ-cadinene	1797[c]	1803[c]	0.42 (0.02)	0.26 (0.03)	0.31 (0.03)	0.29 (0.02)	0.33 (0.02)	0.26 (0.02)
	Total			96.24 (1.96)	96.22 (2.30)	95.79 (2.08)	95.81 (2.07)	96.71 (2.00)	96.93 (2.36)
	% Identified			77.97 (1.19)	77.88 (1.37)	77.55 (1.20)	77.09 (1.26)	77.81 (1.22)	78.25 (1.43)
	Including:								
	Aliphatics			2.61 (0.25)	2.60 (0.28)	2.53 (0.20)	2.51 (0.17)	2.37 (0.19)	2.38 (0.20)
	Aromatics			3.59 (012)	3.45 (0.20)	3.61 (0.10)	3.53 (0.13)	3.61 (0.16)	3.42 (0.17)
	Monoterpene hydrocarbons			0.03 (0.01)	0.02 (0.01)	0.02 (0.01)	0.06 (0.01)	0.02 (0.01)	0.03 (0.01)
	Monoterpenoide hydrocarbons			0.09 (0.02)	0.06 (0.01)	0.06 (0.03)	0.08 (0.01)	0.06 (0.01)	0.09 (0.02)
	Sesquiterpene hydrocarbons			49.87 (0.58)	50.43 (0.63)	49,24 (0.63)	49,87 (0.69)	50.22 (0.59)	51.19 (0.68)
	Sesquiterpenoide hydrocarbons			21.78 (0.22)	21.32 (0.24)	22.09 (0.23)	21.04 (0.25)	21.53 (0.26)	21.14 (0.35)

- less than 0.01%. * The names of terpenes and terpenoids according to IUPAC terminology are given in Table S5. ** Retention index on Quadex 007-5MS column. *** Literature retention index. ND No data. **** For abbreviations of samples see Table 1. ( ) standard deviation. Identification of compounds by MS databases ([a] - NIST 2011, [b] - NIST Chemistry WebBook, [c] - Adams 4 Library, [d] - Pherobase).

Table S2b. Volatile compounds detected in the samples collected in spring (CI-45 – CI-50).

No.	Compounds*	RI**	RI***	Code****					
				CI-45	CI-46	CI-47	CI-48	CI-49	CI-50
1	propan-1-ol	<700[a,b]	483[a]	1.13 (0.03)	1.08 (0.04)	1.05 (0.03)	1.13 (0.03)	1.13 (0.02)	1.01 (0.02)
2	pentanal	705[a,b,c,d]	704[c]	0.41 (0.03)	0.29 (0.02)	0.29 (0.02)	0.35 (0.02)	0.41 (0.03)	0.27 (0.02)
3	hexanal	802[a,b,c,d]	801[c]	0.23 (0.04)	0.17 (0.04)	0.20 (0.04)	0.22 (0.03)	0.18 (0.02)	0.19 (0.03)
4	hexan-1-ol	867[a,b]	869[a]	0.48 (0.03)	0.49 (0.03)	0.44 (0.03)	0.54 (0.02)	0.47 (0.03)	0.52 (0.02)
5	heptanal	902[a,b,c,d]	901[c]	0.09 (0.01)	0.07 (0.01)	0.08 (0.01)	0.03 (0.01)	0.06 (0.01)	0.06 (0.01)
6	α-pinene	939[a,b,c]	932[c]	0.06 (0.01)	0.03 (0.01)	0.04 (0.01)	0.03 (0.01)	0.01 (0.01)	0.05 (0.01)
7	benzaldehyde	940[a,b,c]	952[c]	0.13 (0.02)	0.22 (0.04)	0.21 (0.02)	0.29 (0.01)	0.15 (0.02)	0.26 (0.03)
8	2-ethylhexan-1-ol	1023[a,b]	1025[a]	0.06 (0.03)	0.15 (0.01)	0.15 (0.01)	0.08 (0.02)	0.07 (0.03)	0.05 (0.01)
9	phenylmethanol	1028[a,b,c]	1026[c]	1.06 (0.05)	1.16 (0.04)	1.23 (0.03)	1.05 (0.04)	1.05 (0.03)	1.13 (0.04)
10	phenylacetaldehyde	1044[a,b]	1044[a]	1.33 (0.03)	1.17 (0.04)	1.00 (0.04)	1.09 (0.04)	1.20 (0.04)	1.11 (0.03)
11	nonanal	1102[a,b,c,d]	1100[c]	0.08 (0.01)	0.08 (0.02)	0.17 (0.01)	0.16 (0.03)	0.15 (0.03)	0.12 (0.01)
12	3,4-dimethylcyclohexan-1-ol	1115[a,b]	1126[a]	0.07 (0.01)	0.13 (0.03)	0.11 (0.03)	0.15 (0.02)	0.12 (0.02)	0.08 (0.01)
13	phenylethanol	1121[a,b]	1121[a]	0.09 (0.03)	0.12 (0.04)	0.15 (0.03)	0.15 (0.01)	0.11 (0.01)	0.13 (0.03)
14	decanal	1195[a,b,c,d]	1201[c]	0.10 (0.03)	0.09 (0.02)	0.07 (0.02)	0.03 (0.01)	0.07 (0.02)	0.12 (0.03)
15	β-cyclocitral	1221[c]	1217[c]	0.05 (0.01)	0.10 (0.01)	0.09 (0.01)	0.12 (0.03)	0.06 (0.01)	0.07 (0.02)
16	2-phenoxyethan-1-ol	1225[a,b]	1226[a]	0.91 (0.04)	0.96 (0.05)	1.01 (0.05)	1.03 (0.05)	0.91 (0.03)	0.96 (0.04)
17	bicycloelemene	1316[a]	1330[a]	0.15 (0.02)	0.19 (0.04)	0.15 (0.01)	0.11 (0.01)	0.14 (0.02)	0.12 (0.02)
18	δ-elemene	1324[a,b,c]	1335[c]	0.81 (0.02)	0.81 (0.05)	0.77 (0.03)	0.73 (0.05)	0.72 (0.03)	0.66 (0.03)
19	204[M+](5) 121(100) 93(89)	1343	ND	0.14 (0.03)	0.17 (0.02)	0.25 (0.04)	0.26 (0.04)	0.17 (0.03)	0.15 (0.01)
20	200[M+](39) 159(100) 117(95)	1345	ND	1.13 (0.04)	1.21 (0.03)	0.99 (0.03)	1.04 (0.03)	1.06 (0.04)	0.99 (0.02)
21	202[M+](13) 81(100) 96(73)	1350	ND	0.11 (0.01)	0.16 (0.05)	0.17 (0.01)	0.18 (0.02)	0.16 (0.02)	0.11 (0.01)
22	204[M+](10) 119(100) 91(84)	1353	ND	0.05 (0.01)	0.10 (0.01)	0.08 (0.01)	0.05 (0.01)	0.02 (0.01)	0.09 (0.01)
23	anastreptene	1370[a]	1370[a]	18.16 (0.07)	17.98 (0.06)	17.92 (0.07)	18.25 (0.05)	17.96 (0.05)	18.12 (0.06)
24	204[M+](5) 81(100) 93(96)	1384	ND	0.24 (0.03)	0.16 (0.03)	0.26 (0.03)	0.32 (0.02)	0.18 (0.02)	0.22 (0.03)
25	β-elemene	1391[a,b,c]	1389[c]	2.69 (0.03)	2.89 (0.05)	2.82 (0.04)	2.86 (0.04)	2.69 (0.04)	2.79 (0.04)
26	204[M+](13) 157(100) 185(84)	1398	ND	0.21 (0.02)	0.31 (0.04)	0.36 (0.01)	0.32 (0.03)	0.43 (0.03)	0.23 (0.03)
27	204[M+](13) 157(100) 185(84)	1417	ND	0.18 (0.01)	0.28 (0.03)	0.29 (0.03)	0.34 (0.03)	0.45 (0.04)	0.28 (0.03)
28	204[M+](19) 135(100) 105(82)	1423	ND	0.19 (0.01)	0.19 (0.05)	0.28 (0.04)	0.15 (0.03)	0.24 (0.03)	0.25 (0.04)
29	204[M+](9) 91(100) 105(93)	1425	ND	0.08 (0.02)	0.05 (0.01)	0.05 (0.01)	0.08 (0.01)	0.09 (0.03)	0.03 (0.01)
30	(-)-aristolene	1429[a,b,c,d]	1428[a]	0.93 (0.04)	0.96 (0.05)	1.02 (0.02)	1.06 (0.05)	1.10 (0.06)	1.13 (0.03)
31	204[M+](9) 107(100) 79(43)	1432	ND	0.15 (0.03)	0.19 (0.05)	0.23 (0.03)	0.25 (0.02)	0.11 (0.02)	0.15 (0.02)
32	γ-maaliene	1435[a,b]	1427[a]	0.44 (0.05)	0.52 (0.04)	0.54 (0.02)	0.57 (0.03)	0.62 (0.04)	0.53 (0.04)
33	α-maaliene	1443[a,b]	1442[a]	0.32 (0.03)	0.31 (0.04)	0.29 (0.01)	0.34 (0.04)	0.34 (0.04)	0.34 (0.02)
34	aromandendrene	1445[a,b]	1447[a]	2.98 (0.04)	3.33 (0.05)	3.13 (0.03)	3.12 (0.05)	3.39 (0.05)	3.31 (0.04)
35	selina-5,11-diene	1447[a,b]	1454[a]	0.52 (0.02)	0.53 (0.02)	0.56 (0.02)	0.61 (0.02)	0.74 (0.04)	0.55 (0.05)
36	dehydroaromadendrene	1456[c]	1460[c]	1.16 (0.06)	1.11 (0.03)	1.25 (0.03)	1.18 (0.04)	1.23 (0.02)	1.03 (0.02)
37	1,2,9,10-tetradehydroaristolane	1461	ND	0.39 (0.04)	0.42 (0.05)	0.44 (0.02)	0.43 (0.06)	0.47 (0.02)	0.55 (0.03)
38	204[M+](15) 91(100) 105(84)	1465	ND	0.47 (0.03)	0.51 (0.05)	0.31 (0.03)	0.25 (0.03)	0.22 (0.02)	0.30 (0.02)
39	204[M+](18) 128(100) 143(95)	1469	ND	0.32 (0.05)	0.32 (0.03)	0.29 (0.02)	0.32 (0.04)	0.34 (0.01)	0.32 (0.02)
40	γ-gurjunene	1474[c,d]	1475[c]	0.47 (0.02)	0.37 (0.03)	0.57 (0.03)	0.52 (0.02)	0.48 (0.04)	0.51 (0.02)
41	γ-muurolene	1477[c]	1478[c]	0.13 (0.01)	0.13 (0.02)	0.11 (0.01)	0.22 (0.03)	0.21 (0.03)	0.18 (0.01)
42	δ-selinene	1488[c]	1492[c]	1.26 (0.03)	1.49 (0.04)	1.63 (0.02)	1.52 (0.04)	1.53 (0.04)	1.53 (0.05)
43	ledene	1492[a,b,c]	1496[c]	1.76 (0.02)	1.65 (0.03)	1.58 (0.04)	1.63 (0.03)	1.49 (0.03)	1.59 (0.04)
44	204[M+](38) 105(100) 93(96)	1495	ND	0.35 (0.03)	0.32 (0.02)	0.29 (0.03)	0.33 (0.03)	0.34 (0.03)	0.25 (0.03)
45	bicyclogermacrene	1499[a,b,c]	1500[c]	8.44 (0.05)	8.35 (0.05)	8.55 (0.05)	8.31 (0.05)	8.31 (0.05)	8.37 (0.06)
46	204[M+](19) 93(100) 91(95)	1505	ND	0.19 (0.02)	0.16 (0.01)	0.15 (0.02)	0.18 (0.03)	0.21 (0.03)	0.15 (0.02)
47	202[M+](25) 133(100) 91(89)	1509	ND	0.17 (0.03)	0.15 (0.02)	0.27 (0.03)	0.29 (0.03)	0.14 (0.04)	0.27 (0.03)
48	206[M+](14) 191(100) 57(38)	1514	ND	0.24 (0.02)	0.25 (0.03)	0.21 (0.05)	0.18 (0.04)	0.23 (0.02)	0.22 (0.01)
49	202[M+](33) 131(100) 145(53)	1518	ND	0.18 (0.04)	0.23 (0.04)	0.25 (0.02)	0.23 (0.03)	0.26 (0.02)	0.19 (0.03)
50	δ-cadinene	1524[a,b,c]	1522[c]	0.31 (0.03)	0.34 (0.02)	0.34 (0.03)	0.19 (0.03)	0.27 (0.03)	0.34 (0.04)
51	204[M+](5) 91(100) 131(95)	1530	ND	0.10 (0.02)	0.08 (0.01)	0.07 (0.04)	0.15 (0.01)	0.14 (0.02)	0.16 (0.02)
52	200[M+](54) 185(100) 143(91)	1535	ND	0.12 (0.01)	0.16 (0.02)	0.23 (0.03)	0.13 (0.02)	0.13 (0.03)	0.18 (0.03)
53	4,5,9,10-dehydro-isolongifolene	1544[a,b]	1544[a]	6.31 (0.06)	6.28 (0.07)	6.23 (0.05)	6.39 (0.07)	6.42 (0.05)	6.27 (0.06)
54	202[M+](4) 128(100) 157(95)	1547	ND	0.68 (0.03)	0.63 (0.04)	0.69 (0.03)	0.71 (0.05)	0.77 (0.04)	0.63 (0.04)

55	200[M+](8) 171(100) 186(79)	1551	ND	0.19 (0.02)	0.19 (0.02)	0.25 (0.03)	0.18 (0.03)	0.18 (0.03)	0.22 (0.02)
56	200[M+](91) 129(100) 157(88)	1556	ND	0.15 (0.02)	0.13 (0.03)	0.09 (0.01)	0.05 (0.02)	0.03 (0.01)	0.08 (0.02)
57	204[M+](8) 143(100) 157(98)	1559	ND	0.11 (0.01)	0.05 (0.01)	0.08 (0.01)	0.10 (0.02)	0.12 (0.04)	0.15 (0.03)
58	204[M+](82) 173(100) 189(94)	1563	ND	1.36 (0.04)	1.38 (0.05)	1.52 (0.04)	1.34 (0.04)	1.52 (0.03)	1.53 (0.05)
59	palustrol	1567[c]	1567[c]	8.29 (0.02)	8.44 (0.07)	8.46 (0.06)	8.42 (0.06)	8.33 (0.05)	8.33 (0.06)
60	200[M+](11) 79(100) 93(95)	1570	ND	0.86 (0.05)	0.77 (0.04)	0.76 (0.02)	0.52 (0.03)	0.57 (0.03)	0.91 (0.04)
61	204[M+](31) 81(100) 109(88)	1573	ND	2.03 (0.03)	2.12 (0.04)	2.03 (0.04)	2.03 (0.05)	2.13 (0.05)	2.13 (0.03)
62	spathulenol	1576[a,b,c]	1577[c]	7.58 (0.05)	7.83 (0.05)	7.69 (0.07)	7.70 (0.03)	7.31 (0.03)	7.58 (0.05)
63	200[M+](56) 185(100) 143(63)	1581	ND	3.69 (0.03)	3.52 (0.03)	3.73 (0.04)	3.62 (0.04)	3.48 (0.04)	3.72 (0.04)
64	202[M+](4) 91(100) 79(82)	1587	ND	0.55 (0.04)	0.43 (0.02)	0.41 (0.03)	0.54 (0.01)	0.52 (0.02)	0.52 (0.02)
65	globulol	1599[a,b,c,d]	1590[c]	3.01 (0.05)	3.12 (0.04)	2.79 (0.04)	2.92 (0.04)	2.99 (0.04)	3.16 (0.04)
66	200[M+](8) 198(100) 183(84)	1605	ND	0.24 (0.02)	0.33 (0.06)	0.22 (0.03)	0.18 (0.02)	0.26 (0.03)	0.32 (0.02)
67	220[M+](2) 145(100) 200(93)	1609	ND	1.23 (0.03)	1.16 (0.04)	1.32 (0.04)	1.33 (0.02)	1.23 (0.03)	1.34 (0.04)
68	(+)-bisabola-2,10-diene[1,9]oxide	1615[a,b]	1596[a]	0.19 (0.04)	0.26 (0.03)	0.14 (0.02)	0.16 (0.03)	0.17 (0.03)	0.22 (0.02)
69	208[M+](3) 95(100) 85(95)	1621	ND	0.79 (0.03)	0.81 (0.06)	0.73 (0.03)	0.67 (0.05)	0.72 (0.05)	0.76 (0.04)
70	ledene oxide-(II)	1631[a,b]	1631[a]	0.26 (0.02)	0.21 (0.03)	0.23 (0.02)	0.25 (0.04)	0.26 (0.03)	0.34 (0.03)
71	isospathulenol	1635[a,b]	1633[a]	0.63 (0.04)	0.52 (0.03)	0.52 (0.04)	0.51 (0.05)	0.66 (0.05)	0.61 (0.04)
72	220[M+](18) 91(100) 105(83)	1639	ND	1.99 (0.05)	1.66 (0.04)	1.76 (0.05)	1.86 (0.04)	2.04 (0.03)	1.99 (0.05)
73	cubenol	1642[a,b,c,d]	1645[c]	0.47 (0.04)	0.40 (0.03)	0.43 (0.03)	0.42 (0.02)	0.52 (0.03)	0.43 (0.04)
74	220[M+](21) 91(100) 105(82)	1651	ND	0.08 (0.03)	0.07 (0.02)	0.14 (0.02)	0.10 (0.03)	0.13 (0.02)	0.11 (0.02)
75	222[M+](3) 179(100) 121(92)	1655	ND	0.09 (0.01)	0.03 (0.01)	0.02 (0.01)	0.03 (0.01)	0.02 (0.01)	0.05 (0.01)
76	germacra-4(15),5,10(14)-trien-1-α-ol	1660[c]	1685[c]	0.51 (0.02)	0.53 (0.04)	0.59 (0.02)	0.58 (0.04)	0.58 (0.02)	0.62 (0.04)
77	216[M+](31) 145(100) 91(97)	1699	ND	0.42 (0.06)	0.47 (0.04)	0.54 (0.02)	0.41 (0.03)	0.47 (0.03)	0.42 (0.02)
78	1,4-dimethyl-7-(1-methylethyl)-azulene	1790[c]	1779[c]	2.73 (0.02)	2.63 (0.06)	2.76 (0.03)	2.62 (0.05)	2.53 (0.02)	2.75 (0.04)
79	14-hydroxy-δ-cadinene	1797[c]	1803[c]	0.24 (0.01)	0.35 (0.03)	0.40 (0.02)	0.43 (0.02)	0.41 (0.05)	0.36 (0.03)
	Total			96.50 (2.32)	97.01 (2.70)	97.52 (2.23)	97.22 (2.46)	97.13 (2.37)	97.87 (2.32)
	% Identified			77.42 (1.36)	78.26 (1.60)	78.20 (1.27)	78.50 (1.47)	78.02 (1.39)	78.45 (1.41)
	Including:								
	Aliphatics			2.65 (0.22)	2.55 (0.22)	2.56 (0.20)	2.69 (0.19)	2.66 (0.21)	2.42 (0.16)
	Aromatics			3.52 (0.17)	3.63 (0.21)	3.60 (0.17)	3.61 (0.15)	3.42 (0.13)	3.59 (0.17)
	Monoterpene hydrocarbons			0.06 (0.01)	0.03 (0.01)	0.04 (0.01)	0.03 (0.01)	0.01 (0.01)	0.05 (0.01)
	Monoterpenoide hydrocarbons			0.05 (0.01)	0.10 (0.01)	0.09 (0.01)	0.12 (0.03)	0.06 (0.01)	0.07 (0.02)
	Sesquiterpene hydrocarbons			49.96 (0.66)	50.29 (0.80)	50.66 (0.56)	50.66 (0.76)	50.64 (0.70)	50.67 (0.70)
	Sesquiterpenoide hydrocarbons			21.18 (0.29)	21.66 (0.35)	21.25 (0.32)	21.39 (0.33)	21.23 (0.33)	21.65 (0.35)

- less than 0.01%. * The names of terpenes and terpenoids according to IUPAC terminology are given in Table S5. ** Retention index on Quadex 007-5MS column. *** Literature retention index. ND No data. **** For abbreviations of samples see Table 1. ( ) standard deviation. Identification of compounds by MS databases ([a] - NIST 2011, [b] - NIST Chemistry WebBook, [c] - Adams 4 Library, [d] - Pherobase).

Table S3a. Volatile compounds detected in the samples collected in summer (CI-51 – CI-56).

No.	Compounds*	RI**	RI***	Code****					
				CI-51	CI-52	CI-53	CI-54	CI-55	CI-56
1	propan-1-ol	<700[a,b]	483[a]	0.41 (0.02)	0.43 (0.03)	0.41 (0.03)	0.44 (0.03)	0.32 (0.03)	0.28 (0.03)
2	pentanal	705[a,b,c,d]	704[c]	0.35 (0.03)	0.29 (0.03)	0.32 (0.02)	0.29 (0.02)	0.34 (0.03)	0.31 (0.01)
3	hexanal	802[a,b,c,d]	801[c]	0.10 (0.03)	0.06 (0.01)	0.05 (0.01)	0.11 (0.01)	0.07 (0.01)	0.06 (0.02)
4	hexan-1-ol	867[a,b]	869[a]	0.35 (0.03)	0.35 (0.02)	0.31 (0.02)	0.29 (0.02)	0.33 (0.02)	0.32 (0.02)
5	heptanal	902[a,b,c,d]	901[c]	0.06 (0.02)	0.05 (0.01)	0.04 (0.01)	0.04 (0.01)	0.04 (0.01)	0.06 (0.01)
6	α-pinene	939[a,b,c]	932[c]	0.03 (0.01)	0.03 (0.01)	0.02 (0.01)	0.03 (0.01)	0.03 (0.01)	0.02 (0.01)
7	benzaldehyde	940[a,b,c]	952[c]	0.12 (0.03)	0.08 (0.01)	0.08 (0.02)	0.11 (0.02)	0.08 (0.02)	0.09 (0.01)
8	2-ethylhexan-1-ol	1023[a,b]	1025[a]	0.05 (0.01)	0.05 (0.01)	0.03 (0.01)	0.02 (0.01)	0.05 (0.02)	0.06 (0.01)
9	phenylmethanol	1028[a,b,c]	1026[c]	1.07 (0.03)	1.12 (0.04)	0.95 (0.03)	1.01 (0.03)	1.12 (0.02)	1.05 (0.03)
10	phenylacetaldehyde	1044[a,b]	1044[a]	0.23 (0.02)	0.19 (0.03)	0.22 (0.02)	0.20 (0.02)	0.20 (0.01)	0.23 (0.02)
11	nonanal	1102[a,b,c,d]	1100[c]	0.03 (0.01)	0.03 (0.01)	0.01 (0.01)	0.01 (0.01)	0.03 (0.01)	0.04 (0.01)
12	3,4-dimethylcyclohexan-1-ol	1115[a,b]	1126[a]	0.04 (0.01)	0.01 (0.01)	0.03 (0.01)	0.02 (0.01)	0.01 (0.01)	0.03 (0.01)
13	phenylethanol	1121[a,b]	1121[a]	0.72 (0.04)	0.72 (0.04)	0.65 (0.04)	0.71 (0.03)	0.65 (0.03)	0.72 (0.05)
14	decanal	1195[a,b,c,d]	1201[c]	0.03 (0.01)	0.03 (0.01)	0.01 (0.01)	0.02 (0.01)	0.03 (0.01)	0.06 (0.01)
15	β-cyclocitral	1221[c]	1217[c]	0.05 (0.01)	0.04 (0.01)	0.04 (0.01)	0.03 (0.01)	0.06 (0.01)	0.02 (0.01)
16	2-phenoxyethan-1-ol	1225[a,b]	1226[a]	0.43 (0.02)	0.39 (0.04)	0.39 (0.04)	0.39 (0.04)	0.36 (0.04)	0.04 (0.03)
17	bicycloelemene	1316[a]	1330[a]	0.09 (0.01)	0.11 (0.02)	0.09 (0.03)	0.07 (0.03)	0.07 (0.02)	0.07 (0.01)
18	δ-elemene	1324[a,b,c]	1335[c]	1.33 (0.05)	2.23 (0.03)	1.92 (0.02)	1.91 (0.04)	2.05 (0.05)	2.06 (0.06)
19	204[M+](5) 121(100) 93(89)	1343	ND	0.42 (0.03)	0.42 (0.02)	0.39 (0.02)	0.43 (0.03)	0.49 (0.02)	0.49 (0.02)
20	200[M+](39) 159(100) 117(95)	1345	ND	0.92 (0.05)	0.53 (0.03)	0.65 (0.03)	0.59 (0.03)	0.93 (0.03)	0.71 (0.03)
21	202[M+](13) 81(100) 96(73)	1350	ND	0.18 (0.01)	0.23 (0.02)	0.19 (0.04)	0.23 (0.02)	0.23 (0.02)	0.25 (0.02)
22	204[M+](10) 119(100) 91(84)	1353	ND	0.08 (0.01)	0.05 (0.01)	0.06 (0.01)	0.07 (0.02)	0.04 (0.01)	0.06 (0.03)
23	anastreptene	1370[a]	1370[a]	25.13 (0.05)	25.06 (0.05)	25.09 (0.06)	25.37 (0.06)	25.13 (0.06)	25.24 (0.06)
24	204[M+](5) 81(100) 93(96)	1384	ND	0.16 (0.05)	0.15 (0.04)	0.11 (0.02)	0.14 (0.02)	0.16 (0.02)	0.18 (0.04)
25	β-elemene	1391[a,b,c]	1389[c]	2.62 (0.03)	1.92 (0.03)	2.03 (0.03)	2.01 (0.04)	2.42 (0.03)	2.49 (0.03)
26	204[M+](13) 157(100) 185(84)	1398	ND	0.33 (0.02)	0.17 (0.02)	0.21 (0.02)	0.20 (0.03)	0.24 (0.02)	0.26 (0.01)
27	204[M+](13) 157(100) 185(84)	1417	ND	0.24 (0.03)	0.31 (0.02)	0.18 (0.02)	0.25 (0.03)	0.27 (0.02)	0.28 (0.02)
28	204[M+](19) 135(100) 105(82)	1423	ND	0.25 (0.01)	0.28 (0.03)	0.34 (0.02)	0.23 (0.03)	0.22 (0.03)	0.26 (0.02)
29	204[M+](9) 91(100) 105(93)	1425	ND	0.09 (0.01)	0.09 (0.01)	0.07 (0.01)	0.09 (0.02)	0.10 (0.01)	0.12 (0.04)
30	(-)-aristolene	1429[a,b,c,d]	1428[a]	0.72 (0.03)	0.73 (0.04)	0.77 (0.03)	0.69 (0.04)	0.73 (0.03)	0.68 (0.03)
31	204[M+](9) 107(100) 79(43)	1432	ND	0.22 (0.04)	0.32 (0.03)	0.23 (0.02)	0.23 (0.04)	0.25 (0.03)	0.18 (0.03)
32	γ-maaliene	1435[a,b]	1427[a]	0.39 (0.03)	0.62 (0.03)	0.38 (0.03)	0.39 (0.03)	0.62 (0.04)	0.49 (0.03)
33	α-maaliene	1443[a,b]	1442[a]	0.31 (0.02)	0.49 (0.03)	0.35 (0.03)	0.47 (0.03)	0.47 (0.05)	0.49 (0.03)
34	aromandendrene	1445[a,b]	1447[a]	6.32 (0.05)	7.92 (0.04)	6.17 (0.04)	7.45 (0.03)	7.12 (0.06)	6.99 (0.04)
35	selina-5,11-diene	1447[a,b]	1454[a]	0.53 (0.03)	0.85 (0.02)	0.65 (0.03)	0.82 (0.02)	0.83 (0.02)	0.81 (0.03)
36	dehydroaromadendrene	1456[c]	1460[c]	1.47 (0.02)	1.62 (0.03)	1.45 (0.04)	1.51 (0.03)	1.43 (0.02)	1.57 (0.02)
37	1,2,9,10-tetradehydroaristolane	1461	ND	1.36 (0.03)	0.94 (0.02)	1.26 (0.03)	1.12 (0.03)	1.23 (0.03)	1.26 (0.03)
38	204[M+](15) 91(100) 105(84)	1465	ND	0.35 (0.02)	0.42 (0.02)	0.32 (0.02)	0.33 (0.02)	0.38 (0.04)	0.36 (0.04)
39	204[M+](18) 128(100) 143(95)	1469	ND	0.44 (0.02)	0.26 (0.03)	0.41 (0.02)	0.41 (0.03)	0.37 (0.02)	0.46 (0.02)
40	γ-gurjunene	1474[c,d]	1475[c]	0.46 (0.03)	0.48 (0.02)	0.39 (0.02)	0.40 (0.03)	0.38 (0.04)	0.43 (0.03)
41	γ-muurolene	1477[c]	1478[c]	0.21 (0.04)	0.25 (0.03)	0.20 (0.02)	0.17 (0.03)	0.27 (0.03)	0.23 (0.03)
42	δ-selinene	1488[c]	1492[c]	2.19 (0.05)	2.26 (0.05)	2.06 (0.04)	2.25 (0.04)	2.21 (0.03)	2.23 (0.04)
43	ledene	1492[a,b,c]	1496[c]	1.99 (0.03)	2.11 (0.03)	2.00 (0.04)	2.09 (0.02)	2.01 (0.02)	2.01 (0.03)
44	204[M+](38) 105(100) 93(96)	1495	ND	0.26 (0.02)	0.12 (0.02)	0.23 (0.03)	0.16 (0.02)	0.16 (0.01)	0.29 (0.03)
45	bicyclogermacrene	1499[a,b,c]	1500[c]	17.68 (0.06)	17.12 (0.05)	17.61 (0.06)	16.78 (0.06)	16.94 (0.06)	17.12 (0.06)
46	204[M+](19) 93(100) 91(95)	1505	ND	0.22 (0.03)	0.59 (0.04)	0.42 (0.02)	0.42 (0.02)	0.55 (0.02)	0.45 (0.03)
47	202[M+](25) 133(100) 91(89)	1509	ND	0.08 (0.01)	0.16 (0.02)	0.09 (0.01)	0.10 (0.01)	0.13 (0.03)	0.19 (0.02)
48	206[M+](14) 191(100) 57(38)	1514	ND	0.12 (0.02)	0.33 (0.03)	0.12 (0.02)	0.25 (0.02)	0.35 (0.03)	0.17 (0.02)
49	202[M+](33) 131(100) 145(53)	1518	ND	0.45 (0.03)	0.41 (0.04)	0.38 (0.03)	0.37 (0.02)	0.41 (0.03)	0.45 (0.02)
50	δ-cadinene	1524[a,b,c]	1522[c]	0.10 (0.01)	0.16 (0.02)	0.12 (0.04)	0.15 (0.02)	0.12 (0.02)	0.09 (0.01)
51	204[M+](5) 91(100) 131(95)	1530	ND	0.22 (0.02)	0.09 (0.02)	0.15 (0.04)	0.20 (0.02)	0.22 (0.03)	0.13 (0.02)
52	200[M+](54) 185(100) 143(91)	1535	ND	-	-	-	-	-	-
53	4,5,9,10-dehydro-isolongifolene	1544[a,b]	1544[a]	4.65 (0.04)	4.08 (0.03)	4.45 (0.03)	4.44 (0.03)	4.32 (0.03)	4.35 (0.03)
54	202[M+](4) 128(100) 157(95)	1547	ND	0.86 (0.03)	0.71 (0.02)	0.81 (0.02)	0.71 (0.03)	0.73 (0.02)	0.81 (0.01)

55	200[M+](8) 171(100) 186(79)	1551	ND	0.09 (0.01)	0.09 (0.02)	0.06 (0.02)	0.11 (0.01)	0.11 (0.02)	0.07 (0.01)
56	200[M+](91) 129(100) 157(88)	1556	ND	0.07 (0.02)	0.06 (0.01)	0.08 (0.01)	0.08 (0.01)	0.08 (0.01)	0.03 (0.01)
57	204[M+](8) 143(100) 157(98)	1559	ND	0.06 (0.02)	0.04 (0.01)	0.03 (0.01)	0.03 (0.01)	0.06 (0.01)	0.07 (0.01)
58	204[M+](82) 173(100) 189(94)	1563	ND	1.25 (0.05)	1.25 (0.03)	1.23 (0.04)	1.25 (0.03)	1.26 (0.03)	1.21 (0.03)
59	palustrol	1567[c]	1567[c]	5.16 (0.04)	5.54 (0.03)	5.52 (0.03)	5.36 (0.04)	5.29 (0.03)	5.34 (0.06)
60	200[M+](11) 79(100) 93(95)	1570	ND	0.67 (0.03)	0.46 (0.02)	0.54 (0.02)	0.47 (0.02)	0.61 (0.04)	0.54 (0.03)
61	204[M+](31) 81(100) 109(88)	1573	ND	2.45 (0.02)	1.89 (0.03)	2.19 (0.03)	2.23 (0.02)	1.92 (0.04)	1.87 (0.03)
62	spathulenol	1576[a,b,c]	1577[c]	2.62 (0.04)	2.64 (0.05)	2.69 (0.03)	2.69 (0.03)	2.64 (0.05)	2.63 (0.04)
63	200[M+](56) 185(100) 143(63)	1581	ND	2.43 (0.03)	2.57 (0.06)	2.46 (0.03)	2.42 (0.02)	2.41 (0.03)	2.58 (0.03)
64	202[M+](4) 91(100) 79(82)	1587	ND	0.32 (0.01)	0.35 (0.02)	0.36 (0.02)	0.29 (0.02)	0.32 (0.03)	0.29 (0.02)
65	globulol	1599[a,b,c,d]	1590[c]	1.48 (0.04)	1.09 (0.03)	1.46 (0.02)	1.36 (0.04)	1.29 (0.03)	1.33 (0.02)
66	200[M+](8) 198(100) 183(84)	1605	ND	-	-	-	-	-	-
67	220[M+](2) 145(100) 200(93)	1609	ND	0.08 (0.01)	0.08 (0.01)	0.09 (0.01)	0.09 (0.02)	0.11 (0.02)	0.08 (0.01)
68	(+)-bisabola-2,10-diene[1,9]oxide	1615[a,b]	1596[a]	0.32 (0.03)	0.33 (0.04)	0.35 (0.02)	0.26 (0.02)	0.29 (0.03)	0.29 (0.03)
69	208[M+](3) 95(100) 85(95)	1621	ND	0.69 (0.03)	0.12 (0.03)	0.62 (0.03)	0.59 (0.03)	0.51 (0.02)	0.53 (0.03)
70	ledene oxide-(II)	1631[a,b]	1631[a]	-	-	-	-	-	-
71	isospathulenol	1635[a,b]	1633[a]	0.27 (0.05)	0.30 (0.04)	0.33 (0.04)	0.35 (0.04)	0.39 (0.02)	0.37 (0.02)
72	220[M+](18) 91(100) 105(83)	1639	ND	0.82 (0.03)	0.78 (0.05)	0.71 (0.03)	0.76 (0.05)	0.69 (0.03)	0.75 (0.03)
73	cubenol	1642[a,b,c,d]	1645[c]	0.12 (0.01)	0.05 (0.01)	0.07 (0.02)	0.09 (0.01)	0.06 (0.01)	0.04 (0.01)
74	220[M+](21) 91(100) 105(82)	1651	ND	0.09 (0.02)	0.04 (0.01)	0.06 (0.01)	0.03 (0.01)	0.02 (0.01)	0.03 (0.01)
75	222[M+](3) 179(100) 121(92)	1655	ND	0.06 (0.01)	0.06 (0.01)	0.04 (0.01)	0.04 (0.01)	0.05 (0.01)	0.04 (0.02)
76	germacra-4(15),5,10(14)-trien-1-α-ol	1660[c]	1685[c]	0.89 (0.05)	0.93 (0.04)	0.91 (0.04)	0.91 (0.04)	0.87 (0.04)	0.95 (0.03)
77	216[M+](31) 145(100) 91(97)	1699	ND	0.17 (0.02)	0.25 (0.03)	0.16 (0.03)	0.21 (0.02)	0.21 (0.03)	0.20 (0.04)
78	1,4-dimethyl-7-(1-methylethyl)-azulene	1790[c]	1779[c]	1.12 (0.06)	1.27 (0.05)	1.25 (0.03)	1.23 (0.03)	1.30 (0.04)	1.24 (0.05)
79	14-hydroxy-δ-cadinene	1797[c]	1803[c]	0.23 (0.02)	0.02 (0.01)	0.18 (0.02)	0.15 (0.02)	0.12 (0.02)	0.17 (0.01)
	Total			98.97 (2.05)	98.67 (2.00)	97.30 (1.89)	98.22 (1.91)	98.91 (1.97)	98.75 (1.94)
	% Identified			83.83 (1.28)	84.99 (1.19)	83.31 (1.17)	84.21 (1.17)	84.32 (1.20)	84.36 (1.16)
	Including:								
	Aliphatics			1.42 (0.17)	1.30 (0.14)	1.21 (0.13)	1.24 (0.13)	1.22 (0.15)	1.22 (0.13)
	Aromatics			2.57 (0.14)	2.50 (0.16)	2.29 (0.15)	2.42 (0.14)	2.41 (0.12)	2.13 (0.14)
	Monoterpene hydrocarbons			0.03 (0.01)	0.03 (0.01)	0.02 (0.01)	0.03 (0.01)	0.03 (0.01)	0.02 (0.01)
	Monoterpenoide hydrocarbons			0.05 (0.01)	0.04 (0.01)	0.04 (0.01)	0.03 (0.01)	0.06 (0.01)	0.02 (0.01)
	Sesquiterpene hydrocarbons			68.67 (0.67)	70.22 (0.62)	68.24 (0.65)	69.32 (0.64)	69.65 (0.68)	69.85 (0.65)
	Sesquiterpenoide hydrocarbons			11.09 (0.28)	10.90 (0.25)	11.51 (0.22)	11.17 (0.24)	10.95 (0.23)	11.12 (0.22)

- less than 0.01%. * The names of terpenes and terpenoids according to IUPAC terminology are given in Table S5. ** Retention index on Quadex 007-5MS column. *** Literature retention index. ND No data. **** For abbreviations of samples see Table 1. ( ) standard deviation. Identification of compounds by MS databases ([a] - NIST 2011, [b] - NIST Chemistry WebBook, [c] - Adams 4 Library, [d] - Pherobase).

Table S3b. Volatile compounds detected in the samples collected in summer (CI-57 – CI-62).

No.	Compounds*	RI**	RI***	Code****					
				CI-57	CI-58	CI-59	CI-60	CI-61	CI-62
1	propan-1-ol	<700[a,b]	483[a]	0.45 (0.03)	0.42 (0.03)	0.38 (0.02)	0.39 (0.03)	0.40 (0.02)	0.41 (0.02)
2	pentanal	705[a,b,c,d]	704[c]	0.28 (0.02)	0.30 (0.02)	0.33 (0.04)	0.34 (0.02)	0.29 (0.03)	0.32 (0.04)
3	hexanal	802[a,b,c,d]	801[c]	0.05 (0.01)	0.08 (0.01)	0.09 (0.01)	0.10 (0.01)	0.11 (0.01)	0.08 (0.02)
4	hexan-1-ol	867[a,b]	869[a]	0.29 (0.03)	0.31 (0.03)	0.34 (0.02)	0.35 (0.02)	0.31 (0.02)	0.36 (0.03)
5	heptanal	902[a,b,c,d]	901[c]	0.05 (0.01)	0.06 (0.01)	0.03 (0.01)	0.05 (0.01)	0.06 (0.01)	0.05 (0.01)
6	α-pinene	939[a,b,c]	932[c]	0.01 (0.01)	0.01 (0.01)	0.01 (0.01)	0.03 (0.01)	0.04 (0.01)	0.06 (0.01)
7	benzaldehyde	940[a,b,c]	952[c]	0.07 (0.01)	0.11 (0.02)	0.09 (0.01)	0.09 (0.02)	0.09 (0.01)	0.09 (0.02)
8	2-ethylhexan-1-ol	1023[a,b]	1025[a]	0.03 (0.01)	0.03 (0.01)	0.04 (0.01)	0.04 (0.01)	0.05 (0.01)	0.06 (0.01)
9	phenylmethanol	1028[a,b,c]	1026[c]	1.03 (0.02)	1.01 (0.05)	1.08 (0.04)	1.08 (0.03)	1.02 (0.03)	1.04 (0.03)
10	phenylacetaldehyde	1044[a,b]	1044[a]	0.25 (0.03)	0.18 (0.02)	0.21 (0.02)	0.21 (0.01)	0.22 (0.02)	0.25 (0.03)
11	nonanal	1102[a,b,c,d]	1100[c]	0.03 (0.01)	0.01 (0.01)	0.01 (0.01)	0.02 (0.01)	0.03 (0.01)	0.02 (0.01)
12	3,4-dimethylcyclohexan-1-ol	1115[a,b]	1126[a]	0.04 (0.01)	0.06 (0.01)	0.05 (0.01)	0.06 (0.02)	0.04 (0.01)	0.03 (0.01)
13	phenylethanol	1121[a,b]	1121[a]	0.71 (0.02)	0.74 (0.02)	0.73 (0.02)	0.73 (0.02)	0.73 (0.01)	0.71 (0.04)
14	decanal	1195[a,b,c,d]	1201[c]	0.03 (0.01)	0.03 (0.01)	0.03 (0.01)	0.02 (0.01)	0.02 (0.01)	0.03 (0.01)
15	β-cyclocitral	1221[c]	1217[c]	0.04 (0.01)	0.04 (0.01)	0.06 (0.01)	0.03 (0.01)	0.03 (0.01)	0.05 (0.01)
16	2-phenoxyethan-1-ol	1225[a,b]	1226[a]	0.37 (0.03)	0.40 (0.03)	0.41 (0.03)	0.40 (0.01)	0.41 (0.02)	0.39 (0.02)
17	bicycloelemene	1316[a]	1330[a]	0.10 (0.01)	0.07 (0.01)	0.07 (0.01)	0.09 (0.01)	0.08 (0.01)	0.09 (0.01)
18	δ-elemene	1324[a,b,c]	1335[c]	2.19 (0.04)	2.18 (0.03)	2.21 (0.04)	1.97 (0.04)	1.82 (0.04)	1.85 (0.05)
19	204[M+](5) 121(100) 93(89)	1343	ND	0.43 (0.03)	0.46 (0.03)	0.39 (0.01)	0.42 (0.03)	0.39 (0.02)	0.45 (0.03)
20	200[M+](39) 159(100) 117(95)	1345	ND	0.83 (0.02)	0.81 (0.01)	0.82 (0.02)	0.73 (0.01)	0.74 (0.03)	0.76 (0.03)
21	202[M+](13) 81(100) 96(73)	1350	ND	0.17 (0.02)	0.21 (0.02)	0.22 (0.01)	0.18 (0.01)	0.19 (0.02)	0.20 (0.04)
22	204[M+](10) 119(100) 91(84)	1353	ND	0.08 (0.02)	0.10 (0.02)	0.09 (0.01)	0.08 (0.01)	0.06 (0.01)	0.06 (0.04)
23	anastreptene	1370[a]	1370[a]	25.29 (0.05)	25.26 (0.05)	25.26 (0.05)	25.28 (0.04)	25.09 (0.05)	25.13 (0.02)
24	204[M+](5) 81(100) 93(96)	1384	ND	0.18 (0.02)	0.18 (0.01)	0.16 (0.02)	0.18 (0.01)	0.19 (0.03)	0.23 (0.03)
25	β-elemene	1391[a,b,c]	1389[c]	2.38 (0.04)	2.01 (0.03)	2.18 (0.04)	2.10 (0.04)	2.02 (0.03)	2.01 (0.02)
26	204[M+](13) 157(100) 185(84)	1398	ND	0.19 (0.02)	0.21 (0.03)	0.23 (0.02)	0.23 (0.02)	0.24 (0.02)	0.23 (0.03)
27	204[M+](13) 157(100) 185(84)	1417	ND	0.24 (0.02)	0.26 (0.02)	0.24 (0.03)	0.27 (0.01)	0.27 (0.02)	0.27 (0.02)
28	204[M+](19) 135(100) 105(82)	1423	ND	0.31 (0.03)	0.27 (0.01)	0.27 (0.04)	0.31 (0.02)	0.32 (0.03)	0.25 (0.01)
29	204[M+](9) 91(100) 105(93)	1425	ND	0.09 (0.01)	0.10 (0.01)	0.11 (0.01)	0.09 (0.01)	0.08 (0.01)	0.11 (0.01)
30	(-)-aristolene	1429[a,b,c,d]	1428[a]	0.76 (0.03)	0.76 (0.03)	0.81 (0.04)	0.79 (0.03)	0.81 (0.02)	0.72 (0.01)
31	204[M+](9) 107(100) 79(43)	1432	ND	0.27 (0.03)	0.30 (0.02)	0.31 (0.03)	0.29 (0.02)	0.32 (0.02)	0.29 (0.04)
32	γ-maaliene	1435[a,b]	1427[a]	0.57 (0.04)	0.57 (0.03)	0.56 (0.03)	0.59 (0.03)	0.56 (0.02)	0.62 (0.06)
33	α-maaliene	1443[a,b]	1442[a]	0.42 (0.02)	0.33 (0.02)	0.43 (0.05)	0.42 (0.02)	0.43 (0.03)	0.43 (0.03)
34	aromandendrene	1445[a,b]	1447[a]	7.54 (0.04)	7.21 (0.05)	6.98 (0.03)	7.55 (0.05)	6.98 (0.05)	6.99 (0.03)
35	selina-5,11-diene	1447[a,b]	1454[a]	0.67 (0.03)	0.75 (0.02)	0.75 (0.03)	0.76 (0.03)	0.74 (0.01)	0.79 (0.04)
36	dehydroaromadendrene	1456[c]	1460[c]	1.39 (0.02)	1.40 (0.05)	1.53 (0.04)	1.59 (0.03)	1.62 (0.03)	1.48 (0.03)
37	1,2,9,10-tetradehydroaristolane	1461	ND	1.04 (0.02)	1.04 (0.02)	0.97 (0.04)	1.19 (0.02)	1.33 (0.03)	1.35 (0.02)
38	204[M+](15) 91(100) 105(84)	1465	ND	0.30 (0.03)	0.30 (0.03)	0.34 (0.05)	0.43 (0.02)	0.42 (0.02)	0.46 (0.03)
39	204[M+](18) 128(100) 143(95)	1469	ND	0.32 (0.02)	0.42 (0.02)	0.43 (0.06)	0.42 (0.01)	0.38 (0.03)	0.41 (0.02)
40	γ-gurjunene	1474[c,d]	1475[c]	0.51 (0.02)	0.51 (0.03)	0.39 (0.03)	0.48 (0.02)	0.47 (0.03)	0.57 (0.06)
41	γ-muurolene	1477[c]	1478[c]	0.19 (0.01)	0.26 (0.02)	0.24 (0.05)	0.25 (0.01)	0.26 (0.02)	0.29 (0.02)
42	δ-selinene	1488[c]	1492[c]	2.10 (0.05)	2.16 (0.03)	2.18 (0.02)	2.19 (0.02)	2.21 (0.05)	2.09 (0.04)
43	ledene	1492[a,b,c]	1496[c]	1.92 (0.03)	1.87 (0.03)	1.93 (0.04)	1.87 (0.02)	1.92 (0.02)	1.83 (0.05)
44	204[M+](38) 105(100) 93(96)	1495	ND	0.18 (0.01)	0.23 (0.04)	0.24 (0.03)	0.24 (0.02)	0.25 (0.03)	0.31 (0.03)
45	bicyclogermacrene	1499[a,b,c]	1500[c]	17.82 (0.05)	17.98 (0.05)	17.32 (0.06)	16.45 (0.03)	17.02 (0.06)	16.96 (0.03)
46	204[M+](19) 93(100) 91(95)	1505	ND	0.37 (0.02)	0.57 (0.02)	0.57 (0.03)	0.53 (0.03)	0.51 (0.04)	0.47 (0.02)
47	202[M+](25) 133(100) 91(89)	1509	ND	0.14 (0.01)	0.11 (0.01)	0.12 (0.02)	0.14 (0.02)	0.13 (0.03)	0.18 (0.01)
48	206[M+](14) 191(100) 57(38)	1514	ND	0.09 (0.01)	0.13 (0.01)	0.08 (0.04)	0.22 (0.03)	0.27 (0.04)	0.31 (0.05)
49	202[M+](33) 131(100) 145(53)	1518	ND	0.44 (0.03)	0.37 (0.02)	0.39 (0.04)	0.37 (0.03)	0.36 (0.03)	0.43 (0.04)
50	δ-cadinene	1524[a,b,c]	1522[c]	0.14 (0.01)	0.12 (0.01)	0.16 (0.01)	0.14 (0.02)	0.11 (0.02)	0.15 (0.02)
51	204[M+](5) 91(100) 131(95)	1530	ND	0.19 (0.01)	0.16 (0.02)	0.15 (0.03)	0.16 (0.04)	0.16 (0.02)	0.18 (0.04)
52	200[M+](54) 185(100) 143(91)	1535	ND	-	-	-	-	-	-
53	4,5,9,10-dehydro-isolongifolene	1544[a,b]	1544[a]	4.35 (0.04)	4.32 (0.04)	4.32 (0.03)	4.34 (0.05)	4.43 (0.04)	4.41 (0.06)
54	202[M+](4) 128(100) 157(95)	1547	ND	0.76 (0.02)	0.71 (0.02)	0.69 (0.04)	0.73 (0.03)	0.78 (0.03)	0.71 (0.03)

55	200[M+](8) 171(100) 186(79)	1551	ND	0.11 (0.02)	0.08 (0.01)	0.11 (0.01)	0.09 (0.01)	0.09 (0.02)	0.13 (0.04)
56	200[M+](91) 129(100) 157(88)	1556	ND	0.06 (0.03)	0.04 (0.01)	0.09 (0.01)	0.04 (0.01)	0.06 (0.01)	0.08 (0.01)
57	204[M+](8) 143(100) 157(98)	1559	ND	0.07 (0.01)	0.02 (0.01)	0.06 (0.01)	0.06 (0.02)	0.05 (0.01)	0.07 (0.01)
58	204[M+](82) 173(100) 189(94)	1563	ND	1.23 (0.02)	1.19 (0.03)	1.19 (0.04)	1.24 (0.03)	1.25 (0.04)	1.26 (0.03)
59	palustrol	1567[c]	1567[c]	5.09 (0.04)	5.10 (0.04)	5.02 (0.04)	5.19 (0.02)	5.04 (0.04)	5.43 (0.04)
60	200[M+](11) 79(100) 93(95)	1570	ND	0.43 (0.02)	0.63 (0.03)	0.57 (0.01)	0.50 (0.02)	0.61 (0.03)	0.46 (0.02)
61	204[M+](31) 81(100) 109(88)	1573	ND	1.95 (0.03)	2.14 (0.03)	2.08 (0.01)	2.23 (0.03)	2.14 (0.03)	2.13 (0.03)
62	spathulenol	1576[a,b,c]	1577[c]	2.62 (0.06)	2.67 (0.04)	2.64 (0.05)	2.47 (0.04)	2.67 (0.03)	2.73 (0.03)
63	200[M+](56) 185(100) 143(63)	1581	ND	2.45 (0.05)	2.44 (0.03)	2.42 (0.04)	2.44 (0.02)	2.48 (0.02)	2.40 (0.04)
64	202[M+](4) 91(100) 79(82)	1587	ND	0.31 (0.02)	0.27 (0.03)	0.30 (0.01)	0.29 (0.02)	0.30 (0.02)	0.28 (0.03)
65	globulol	1599[a,b,c,d]	1590[c]	1.39 (0.03)	1.38 (0.02)	1.53 (0.03)	1.48 (0.03)	1.49 (0.03)	1.39 (0.04)
66	200[M+](8) 198(100) 183(84)	1605	ND	-	-	-	-	-	-
67	220[M+](2) 145(100) 200(93)	1609	ND	0.10 (0.01)	0.08 (0.02)	0.09 (0.01)	0.07 (0.01)	0.08 (0.01)	0.07 (0.03)
68	(+)-bisabola-2,10-diene[1,9]oxide	1615[a,b]	1596[a]	0.32 (0.02)	0.27 (0.03)	0.28 (0.03)	0.28 (0.02)	0.28 (0.05)	0.27 (0.02)
69	208[M+](3) 95(100) 85(95)	1621	ND	0.46 (0.02)	0.61 (0.04)	0.69 (0.03)	0.54 (0.03)	0.53 (0.04)	0.52 (0.02)
70	ledene oxide-(II)	1631[a,b]	1631[a]	-	-	-	-	-	-
71	isospathulenol	1635[a,b]	1633[a]	0.29 (0.02)	0.37 (0.04)	0.39 (0.02)	0.33 (0.02)	0.39 (0.01)	0.34 (0.01)
72	220[M+](18) 91(100) 105(83)	1639	ND	0.73 (0.02)	0.77 (0.06)	0.69 (0.03)	0.73 (0.03)	0.77 (0.03)	0.62 (0.03)
73	cubenol	1642[a,b,c,d]	1645[c]	0.09 (0.01)	0.09 (0.01)	0.06 (0.01)	0.08 (0.01)	0.09 (0.02)	0.13 (0.03)
74	220[M+](21) 91(100) 105(82)	1651	ND	0.04 (0.02)	0.03 (0.01)	0.04 (0.01)	0.03 (0.01)	0.04 (0.01)	0.05 (0.01)
75	222[M+](3) 179(100) 121(92)	1655	ND	0.07 (0.02)	0.04 (0.01)	0.04 (0.01)	0.03 (0.01)	0.06 (0.01)	0.02 (0.01)
76	germacra-4(15),5,10(14)-trien-1-α-ol	1660[c]	1685[c]	0.95 (0.02)	0.78 (0.02)	0.89 (0.04)	0.87 (0.03)	0.95 (0.01)	0.92 (0.04)
77	216[M+](31) 145(100) 91(97)	1699	ND	0.15 (0.03)	0.23 (0.04)	0.21 (0.02)	0.25 (0.02)	0.22 (0.03)	0.25 (0.04)
78	1,4-dimethyl-7-(1-methylethyl)-azulene	1790[c]	1779[c]	1.24 (0.03)	1.11 (0.02)	1.23 (0.03)	1.18 (0.03)	1.33 (0.06)	1.14 (0.04)
79	14-hydroxy-δ-cadinene	1797[c]	1803[c]	0.08 (0.01)	0.09 (0.02)	0.05 (0.01)	0.18 (0.02)	0.11 (0.02)	0.16 (0.04)
	Total			98.92 (1.76)	98.92 (1.82)	98.70 (1.97)	98.61 (1.63)	98.84 (1.86)	98.87 (2.11)
	% Identified			85.18 (1.05)	84.45 (1.09)	84.27 (1.18)	84.05 (0.98)	84.10 (1.07)	84.22 (1.21)
	Including:								
	Aliphatics			1.25 (0.14)	1.30 (0.14)	1.30 (0.14)	1.37 (0.14)	1.31 (0.13)	1.36 (0.16)
	Aromatics			2.43 (0.11)	2.44 (0.14)	2.52 (0.12)	2.51 (0.09)	2.47 (0.09)	2.48 (0.14)
	Monoterpene hydrocarbons			0.01 (0.01)	0.01 (0.01)	0.01 (0.01)	0.03 (0.01)	0.04 (0.01)	0.06 (0.01)
	Monoterpenoide hydrocarbons			0.04 (0.01)	0.04 (0.01)	0.06 (0.01)	0.03 (0.01)	0.03 (0.01)	0.05 (0.01)
	Sesquiterpene hydrocarbons			70.62 (0.58)	69.91 (0.57)	69.52 (0.67)	69.23 (0.54)	69.23 (0.62)	68.90 (0.64)
	Sesquiterpenoide hydrocarbons			10.83 (0.21)	10.75 (0.22)	10.86 (0.23)	10.88 (0.19)	11.02 (0.21)	11.37 (0.25)

- less than 0.01%. * The names of terpenes and terpenoids according to IUPAC terminology are given in Table S5. ** Retention index on Quadex 007-5MS column. *** Literature retention index. ND No data. **** For abbreviations of samples see Table 1. ( ) standard deviation. Identification of compounds by MS databases ([a] - NIST 2011, [b] - NIST Chemistry WebBook, [c] - Adams 4 Library, [d] - Pherobase).

Table S4a. Volatile compounds detected in the samples collected in autumn (CI-63 – CI-68).

No.	Compounds*	RI**	RI***	Code****					
				CI-63	CI-64	CI-65	CI-66	CI-67	CI-68
1	propan-1-ol	<700[a,b]	483[a]	1.32 (0.03)	1.25 (0.03)	1.41 (0.03)	1.37 (0.03)	1.21 (0.04)	1.41 (0.05)
2	pentanal	705[a,b,c,d]	704[c]	0.44 (0.02)	0.45 (0.02)	0.38 (0.02)	0.42 (0.02)	0.42 (0.02)	0.38 (0.02)
3	hexanal	802[a,b,c,d]	801[c]	0.38 (0.02)	0.34 (0.01)	0.42 (0.03)	0.40 (0.02)	0.43 (0.03)	0.40 (0.03)
4	hexan-1-ol	867[a,b]	869[a]	0.28 (0.03)	0.28 (0.03)	0.25 (0.02)	0.32 (0.03)	0.29 (0.02)	0.31 (0.02)
5	heptanal	902[a,b,c,d]	901[c]	0.04 (0.01)	0.06 (0.01)	0.03 (0.01)	0.01 (0.01)	0.03 (0.01)	0.04 (0.01)
6	α-pinene	939[a,b,c]	932[c]	0.05 (0.01)	0.05 (0.01)	0.05 (0.01)	0.03 (0.01)	0.04 (0.01)	0.04 (0.01)
7	benzaldehyde	940[a,b,c]	952[c]	0.49 (0.02)	0.47 (0.02)	0.42 (0.02)	0.45 (0.02)	0.43 (0.02)	0.43 (0.03)
8	2-ethylhexan-1-ol	1023[a,b]	1025[a]	0.22 (0.02)	0.22 (0.01)	0.19 (0.02)	0.19 (0.01)	0.24 (0.03)	0.23 (0.02)
9	phenylmethanol	1028[a,b,c]	1026[c]	0.99 (0.03)	1.06 (0.03)	1.03 (0.02)	0.99 (0.03)	1.02 (0.03)	1.04 (0.04)
10	phenylacetaldehyde	1044[a,b]	1044[a]	1.72 (0.03)	1.75 (0.03)	1.69 (0.03)	1.72 (0.03)	1.72 (0.03)	1.74 (0.03)
11	nonanal	1102[a,b,c,d]	1100[c]	0.12 (0.01)	0.15 (0.01)	0.13 (0.01)	0.08 (0.01)	0.09 (0.01)	0.10 (0.01)
12	3,4-dimethylcyclohexan-1-ol	1115[a,b]	1126[a]	0.13 (0.01)	0.09 (0.01)	0.09 (0.01)	0.08 (0.01)	0.11 (0.01)	0.12 (0.01)
13	phenylethanol	1121[a,b]	1121[a]	0.56 (0.02)	0.54 (0.02)	0.58 (0.03)	0.56 (0.03)	0.62 (0.03)	0.59 (0.03)
14	decanal	1195[a,b,c,d]	1201[c]	0.06 (0.01)	0.07 (0.01)	0.06 (0.01)	0.09 (0.01)	0.09 (0.01)	0.09 (0.01)
15	β-cyclocitral	1221[c]	1217[c]	0.04 (0.01)	0.05 (0.01)	0.05 (0.01)	0.04 (0.01)	0.04 (0.01)	0.05 (0.01)
16	2-phenoxyethan-1-ol	1225[a,b]	1226[a]	1.32 (0.04)	1.28 (0.03)	1.24 (0.05)	1.28 (0.05)	1.30 (0.04)	1.29 (0.02)
17	bicycloelemene	1316[a]	1330[a]	0.09 (0.01)	0.09 (0.02)	0.08 (0.01)	0.08 (0.02)	0.10 (0.01)	0.09 (0.01)
18	δ-elemene	1324[a,b,c]	1335[c]	0.68 (0.02)	0.73 (0.03)	0.65 (0.03)	0.68 (0.03)	0.70 (0.02)	0.72 (0.03)
19	204[M+](5) 121(100) 93(89)	1343	ND	0.12 (0.02)	0.12 (0.02)	0.12 (0.01)	0.14 (0.01)	0.13 (0.01)	0.15 (0.01)
20	200[M+](39) 159(100) 117(95)	1345	ND	1.31 (0.03)	1.21 (0.04)	1.29 (0.05)	1.26 (0.02)	1.21 (0.05)	1.23 (0.04)
21	202[M+](13) 81(100) 96(73)	1350	ND	0.11 (0.01)	0.11 (0.01)	0.11 (0.02)	0.12 (0.01)	0.09 (0.01)	0.09 (0.01)
22	204[M+](10) 119(100) 91(84)	1353	ND	0.10 (0.01)	0.08 (0.01)	0.08 (0.01)	0.09 (0.01)	0.11 (0.02)	0.09 (0.01)
23	anastreptene	1370[a]	1370[a]	15.82 (0.06)	15.83 (0.05)	15.97 (0.06)	15.69 (0.06)	15.32 (0.04)	15.58 (0.04)
24	204[M+](5) 81(100) 93(96)	1384	ND	0.16 (0.01)	0.16 (0.03)	0.15 (0.01)	0.14 (0.02)	0.22 (0.02)	0.20 (0.01)
25	β-elemene	1391[a,b,c]	1389[c]	1.23 (0.03)	1.31 (0.04)	1.28 (0.02)	1.26 (0.04)	1.26 (0.04)	1.26 (0.05)
26	204[M+](13) 157(100) 185(84)	1398	ND	0.24 (0.02)	0.27 (0.02)	0.19 (0.01)	0.21 (0.01)	0.22 (0.02)	0.26 (0.03)
27	204[M+](13) 157(100) 185(84)	1417	ND	0.31 (0.03)	0.31 (0.03)	0.33 (0.03)	0.33 (0.02)	0.36 (0.02)	0.35 (0.01)
28	204[M+](19) 135(100) 105(82)	1423	ND	0.13 (0.02)	0.13 (0.02)	0.11 (0.01)	0.11 (0.01)	0.13 (0.01)	0.19 (0.01)
29	204[M+](9) 91(100) 105(93)	1425	ND	0.06 (0.01)	0.05 (0.01)	0.02 (0.01)	0.02 (0.01)	0.06 (0.01)	0.05 (0.01)
30	(-)-aristolene	1429[a,b,c,d]	1428[a]	1.17 (0.03)	1.17 (0.02)	1.21 (0.04)	1.18 (0.04)	1.12 (0.03)	1.18 (0.03)
31	204[M+](9) 107(100) 79(43)	1432	ND	0.09 (0.01)	0.12 (0.01)	0.11 (0.02)	0.10 (0.02)	0.11 (0.02)	0.15 (0.02)
32	γ-maaliene	1435[a,b]	1427[a]	0.17 (0.02)	0.26 (0.01)	0.26 (0.02)	0.25 (0.02)	0.19 (0.02)	0.21 (0.01)
33	α-maaliene	1443[a,b]	1442[a]	0.23 (0.02)	0.27 (0.03)	0.18 (0.02)	0.22 (0.02)	0.25 (0.02)	0.28 (0.01)
34	aromandendrene	1445[a,b]	1447[a]	3.18 (0.05)	3.22 (0.04)	3.22 (0.02)	3.23 (0.04)	3.19 (0.04)	3.34 (0.04)
35	selina-5,11-diene	1447[a,b]	1454[a]	0.27 (0.02)	0.34 (0.03)	0.34 (0.03)	0.25 (0.02)	0.29 (0.03)	0.28 (0.01)
36	dehydroaromadendrene	1456[c]	1460[c]	1.21 (0.02)	1.16 (0.02)	1.13 (0.04)	1.09 (0.04)	1.12 (0.04)	1.19 (0.04)
37	1,2,9,10-tetradehydroaristolane	1461	ND	0.52 (0.02)	0.56 (0.02)	0.46 (0.03)	0.52 (0.02)	0.46 (0.03)	0.51 (0.02)
38	204[M+](15) 91(100) 105(84)	1465	ND	0.27 (0.01)	0.26 (0.01)	0.24 (0.02)	0.28 (0.01)	0.31 (0.02)	0.33 (0.02)
39	204[M+](18) 128(100) 143(95)	1469	ND	0.34 (0.01)	0.37 (0.01)	0.36 (0.02)	0.36 (0.02)	0.35 (0.03)	0.29 (0.02)
40	γ-gurjunene	1474[c,d]	1475[c]	0.29 (0.02)	0.28 (0.02)	0.33 (0.02)	0.34 (0.02)	0.33 (0.02)	0.29 (0.02)
41	γ-muurolene	1477[c]	1478[c]	0.11 (0.02)	0.09 (0.01)	0.09 (0.01)	0.11 (0.01)	0.09 (0.02)	0.14 (0.01)
42	δ-selinene	1488[c]	1492[c]	1.01 (0.03)	1.03 (0.03)	0.99 (0.03)	1.06 (0.04)	1.11 (0.04)	1.09 (0.05)
43	ledene	1492[a,b,c]	1496[c]	1.32 (0.03)	1.47 (0.04)	1.42 (0.02)	1.47 (0.03)	1.41 (0.05)	1.38 (0.04)
44	204[M+](38) 105(100) 93(96)	1495	ND	0.08 (0.02)	0.06 (0.01)	0.07 (0.01)	0.09 (0.01)	0.11 (0.01)	0.09 (0.02)
45	bicyclogermacrene	1499[a,b,c]	1500[c]	7.23 (0.03)	7.16 (0.05)	7.23 (0.03)	7.62 (0.04)	7.45 (0.04)	7.69 (0.05)
46	204[M+](19) 93(100) 91(95)	1505	ND	0.09 (0.01)	0.16 (0.01)	0.12 (0.01)	0.10 (0.01)	0.15 (0.02)	0.19 (0.01)
47	202[M+](25) 133(100) 91(89)	1509	ND	0.14 (0.01)	0.13 (0.01)	0.18 (0.01)	0.22 (0.02)	0.18 (0.03)	0.22 (0.02)
48	206[M+](14) 191(100) 57(38)	1514	ND	0.08 (0.01)	0.16 (0.02)	0.12 (0.01)	0.11 (0.01)	0.12 (0.01)	0.19 (0.04)
49	202[M+](33) 131(100) 145(53)	1518	ND	0.20 (0.02)	0.23 (0.02)	0.19 (0.01)	0.14 (0.01)	0.22 (0.02)	0.21 (0.02)
50	δ-cadinene	1524[a,b,c]	1522[c]	0.32 (0.03)	0.33 (0.03)	0.32 (0.03)	0.32 (0.04)	0.34 (0.04)	0.30 (0.02)
51	204[M+](5) 91(100) 131(95)	1530	ND	0.12 (0.01)	0.06 (0.01)	0.08 (0.01)	0.09 (0.01)	0.12 (0.01)	0.09 (0.04)
52	200[M+](54) 185(100) 143(91)	1535	ND	0.18 (0.02)	0.14 (0.01)	0.20 (0.01)	0.19 (0.01)	0.18 (0.02)	0.25 (0.03)
53	4,5,9,10-dehydro-isolongifolene	1544[a,b]	1544[a]	8.67 (0.04)	8.32 (0.05)	8.23 (0.02)	8.37 (0.05)	8.53 (0.04)	8.54 (0.05)
54	202[M+](4) 128(100) 157(95)	1547	ND	1.43 (0.02)	1.55 (0.04)	1.43 (0.04)	1.53 (0.05)	1.57 (0.03)	1.54 (0.04)

55	200[M+](8) 171(100) 186(79)	1551	ND	0.18 (0.02)	0.19 (0.02)	0.22 (0.01)	0.21 (0.02)	0.17 (0.02)	0.24 (0.01)
56	200[M+](91) 129(100) 157(88)	1556	ND	0.09 (0.01)	0.12 (0.01)	0.11 (0.01)	0.11 (0.01)	0.11 (0.02)	0.09 (0.02)
57	204[M+](8) 143(100) 157(98)	1559	ND	0.05 (0.01)	0.05 (0.01)	0.06 (0.01)	0.05 (0.01)	0.05 (0.01)	0.07 (0.01)
58	204[M+](82) 173(100) 189(94)	1563	ND	1.64 (0.05)	1.58 (0.02)	1.54 (0.04)	1.62 (0.03)	1.61 (0.04)	1.56 (0.04)
59	palustrol	1567[c]	1567[c]	9.87 (0.05)	9.81 (0.05)	9.83 (0.05)	9.83 (0.03)	9.92 (0.04)	9.83 (0.05)
60	200[M+](11) 79(100) 93(95)	1570	ND	0.47 (0.03)	0.53 (0.03)	0.48 (0.02)	0.51 (0.04)	0.52 (0.02)	0.47 (0.03)
61	204[M+](31) 81(100) 109(88)	1573	ND	0.89 (0.02)	0.94 (0.02)	0.95 (0.03)	0.92 (0.03)	0.89 (0.02)	0.86 (0.06)
62	spathulenol	1576[a,b,c]	1577[c]	5.03 (0.04)	5.01 (0.03)	5.03 (0.04)	4.97 (0.03)	5.02 (0.05)	4.96 (0.03)
63	200[M+](56) 185(100) 143(63)	1581	ND	5.36 (0.05)	5.39 (0.05)	5.38 (0.03)	5.42 (0.05)	5.62 (0.06)	5.39 (0.04)
64	202[M+](4) 91(100) 79(82)	1587	ND	0.47 (0.02)	0.45 (0.02)	0.43 (0.02)	0.44 (0.02)	0.48 (0.02)	0.49 (0.02)
65	globulol	1599[a,b,c,d]	1590[c]	3.02 (0.04)	3.12 (0.02)	2.98 (0.03)	3.01 (0.02)	2.97 (0.04)	3.01 (0.05)
66	200[M+](8) 198(100) 183(84)	1605	ND	0.31 (0.01)	0.24 (0.01)	0.18 (0.01)	0.23 (0.01)	0.28 (0.01)	0.26 (0.02)
67	220[M+](2) 145(100) 200(93)	1609	ND	2.04 (0.02)	2.11 (0.03)	2.21 (0.02)	2.12 (0.02)	2.03 (0.05)	2.11 (0.06)
68	(+)-bisabola-2,10-diene[1,9]oxide	1615[a,b]	1596[a]	0.22 (0.02)	0.12 (0.01)	0.15 (0.01)	0.18 (0.05)	0.22 (0.01)	0.21 (0.01)
69	208[M+](3) 95(100) 85(95)	1621	ND	1.44 (0.03)	1.36 (0.04)	1.12 (0.04)	1.18 (0.05)	1.36 (0.06)	1.52 (0.03)
70	ledene oxide-(II)	1631[a,b]	1631[a]	0.22 (0.01)	0.21 (0.02)	0.27 (0.02)	0.18 (0.01)	0.18 (0.02)	0.18 (0.02)
71	isospathulenol	1635[a,b]	1633[a]	0.86 (0.03)	0.89 (0.02)	0.99 (0.04)	0.98 (0.03)	0.88 (0.03)	0.87 (0.02)
72	220[M+](18) 91(100) 105(83)	1639	ND	1.66 (0.03)	1.51 (0.03)	1.58 (0.03)	1.58 (0.04)	1.62 (0.05)	1.52 (0.03)
73	cubenol	1642[a,b,c,d]	1645[c]	0.54 (0.02)	0.42 (0.02)	0.55 (0.02)	0.46 (0.02)	0.42 (0.02)	0.39 (0.02)
74	220[M+](21) 91(100) 105(82)	1651	ND	0.10 (0.01)	0.07 (0.01)	0.08 (0.02)	0.09 (0.02)	0.08 (0.01)	0.10 (0.02)
75	222[M+](3) 179(100) 121(92)	1655	ND	0.05 (0.01)	0.04 (0.01)	0.03 (0.01)	0.02 (0.01)	0.04 (0.01)	0.04 (0.01)
76	germacra-4(15),5,10(14)-trien-1-α-ol	1660[c]	1685[c]	0.86 (0.02)	0.97 (0.02)	1.05 (0.04)	0.98 (0.03)	0.96 (0.02)	0.98 (0.04)
77	216[M+](31) 145(100) 91(97)	1699	ND	0.56 (0.02)	0.66 (0.03)	0.58 (0.02)	0.57 (0.02)	0.62 (0.03)	0.64 (0.05)
78	1,4-dimethyl-7-(1-methylethyl)-azulene	1790[c]	1779[c]	3.42 (0.06)	3.24 (0.05)	3.15 (0.03)	3.36 (0.03)	3.31 (0.02)	3.52 (0.06)
79	14-hydroxy-δ-cadinene	1797[c]	1803[c]	0.28 (0.01)	0.38 (0.02)	0.43 (0.05)	0.40 (0.01)	0.43 (0.03)	0.34 (0.02)
	Total			96.87 (1.77)	96.82 (1.78)	96.29 (1.79)	96.82 (1.85)	97.08 (2.02)	98.14 (2.07)
	% Identified			76.00 (1.12)	75.90 (1.09)	75.84 (1.14)	76.12 (1.17)	75.65 (1.20)	76.62 (1.20)
	Including:								
	Aliphatics			2.99 (0.16)	2.91 (0.14)	2.96 (0.16)	2.96 (0.15)	2.91 (0.18)	3.08 (0.18)
	Aromatics			5.08 (0.14)	5.10 (0.13)	4.96 (0.15)	5.00 (0.16)	5.09 (0.15)	5.09 (0.15)
	Monoterpene hydrocarbons			0.05 (0.01)	0.05 (0.01)	0.05 (0.01)	0.03 (0.01)	0.04 (0.01)	0.04 (0.01)
	Monoterpenoide hydrocarbons			0.04 (0.01)	0.05 (0.01)	0.05 (0.01)	0.04 (0.01)	0.04 (0.01)	0.05 (0.01)
	Sesquiterpene hydrocarbons			46.94 (0.56)	46.86 (0.59)	46.54 (0.51)	47.10 (0.61)	46.57 (0.59)	47.59 (0.59)
	Sesquiterpenoide hydrocarbons			20.90 (0.24)	20.93 (0.21)	21.28 (0.30)	20.99 (0.23)	21.00 (0.26)	20.77 (0.26)

- less than 0.01%. * The names of terpenes and terpenoids according to IUPAC terminology are given in Table S5. ** Retention index on Quadex 007-5MS column. *** Literature retention index. ND No data. **** For abbreviations of samples see Table 1. ( ) standard deviation. Identification of compounds by MS databases ([a] - NIST 2011, [b] - NIST Chemistry WebBook, [c] - Adams 4 Library, [d] - Pherobase).

Table S4b. Volatile compounds detected in the samples collected in autumn (CI-69 – CI-74).

No.	Compounds*	RI**	RI***	Code****					
				CI-69	CI-70	CI-71	CI-72	CI-73	CI-74
1	propan-1-ol	<700[a,b]	483[a]	1.30 (0.03)	1.39 (0.04)	1.29 (0.05)	1.32 (0.03)	1.25 (0.04)	1.37 (0.05)
2	pentanal	705[a,b,c,d]	704[c]	0.42 (0.02)	0.39 (0.03)	0.42 (0.03)	0.48 (0.02)	0.44 (0.03)	0.45 (0.02)
3	hexanal	802[a,b,c,d]	801[c]	0.41 (0.02)	0.44 (0.03)	0.43 (0.02)	0.42 (0.02)	0.38 (0.02)	0.34 (0.03)
4	hexan-1-ol	867[a,b]	869[a]	0.29 (0.03)	0.29 (0.03)	0.27 (0.03)	0.31 (0.03)	0.28 (0.02)	0.28 (0.02)
5	heptanal	902[a,b,c,d]	901[c]	0.03 (0.01)	0.03 (0.01)	0.06 (0.01)	0.02 (0.01)	0.05 (0.01)	0.04 (0.01)
6	α-pinene	939[a,b,c]	932[c]	0.05 (0.01)	0.04 (0.01)	0.04 (0.01)	0.04 (0.01)	0.06 (0.01)	0.06 (0.01)
7	benzaldehyde	940[a,b,c]	952[c]	0.49 (0.02)	0.52 (0.03)	0.51 (0.04)	0.49 (0.03)	0.52 (0.03)	0.49 (0.02)
8	2-ethylhexan-1-ol	1023[a,b]	1025[a]	0.25 (0.02)	0.21 (0.02)	0.23 (0.02)	0.21 (0.03)	0.23 (0.02)	0.23 (0.03)
9	phenylmethanol	1028[a,b,c]	1026[c]	1.12 (0.04)	1.13 (0.04)	1.06 (0.03)	1.06 (0.04)	0.98 (0.02)	0.99 (0.04)
10	phenylacetaldehyde	1044[a,b]	1044[a]	1.65 (0.03)	1.71 (0.05)	1.59 (0.03)	1.65 (0.05)	1.65 (0.04)	1.76 (0.05)
11	nonanal	1102[a,b,c,d]	1100[c]	0.12 (0.01)	0.13 (0.02)	0.15 (0.01)	0.13 (0.02)	0.14 (0.02)	0.13 (0.03)
12	3,4-dimethylcyclohexan-1-ol	1115[a,b]	1126[a]	0.09 (0.01)	0.09 (0.01)	0.11 (0.01)	0.10 (0.03)	0.11 (0.01)	0.12 (0.01)
13	phenylethanol	1121[a,b]	1121[a]	0.60 (0.02)	0.61 (0.04)	0.59 (0.03)	0.59 (0.03)	0.62 (0.03)	0.56 (0.02)
14	decanal	1195[a,b,c,d]	1201[c]	0.11 (0.02)	0.08 (0.02)	0.07 (0.01)	0.08 (0.01)	0.07 (0.01)	0.06 (0.01)
15	β-cyclocitral	1221[c]	1217[c]	0.07 (0.01)	0.06 (0.01)	0.04 (0.01)	0.04 (0.01)	0.05 (0.01)	0.05 (0.01)
16	2-phenoxyethan-1-ol	1225[a,b]	1226[a]	1.37 (0.05)	1.22 (0.02)	1.25 (0.04)	1.28 (0.03)	1.31 (0.02)	1.34 (0.04)
17	bicycloelemene	1316[a]	1330[a]	0.07 (0.02)	0.09 (0.01)	0.06 (0.01)	0.06 (0.01)	0.05 (0.01)	0.09 (0.02)
18	δ-elemene	1324[a,b,c]	1335[c]	0.69 (0.05)	0.69 (0.02)	0.73 (0.03)	0.70 (0.02)	0.73 (0.04)	0.72 (0.05)
19	204[M+](5) 121(100) 93(89)	1343	ND	0.13 (0.01)	0.11 (0.01)	0.12 (0.01)	0.12 (0.02)	0.14 (0.01)	0.13 (0.01)
20	200[M+](39) 159(100) 117(95)	1345	ND	1.33 (0.03)	1.33 (0.06)	1.32 (0.03)	1.25 (0.04)	1.27 (0.05)	1.22 (0.03)
21	202[M+](13) 81(100) 96(73)	1350	ND	0.12 (0.01)	0.15 (0.02)	0.14 (0.02)	0.14 (0.02)	0.15 (0.03)	0.08 (0.04)
22	204[M+](10) 119(100) 91(84)	1353	ND	0.08 (0.01)	0.11 (0.02)	0.11 (0.02)	0.13 (0.02)	0.12 (0.02)	0.11 (0.01)
23	anastreptene	1370[a]	1370[a]	15.69 (0.05)	15.62 (0.04)	15.31 (0.04)	15.93 (0.06)	15.63 (0.05)	15.97 (0.06)
24	204[M+](5) 81(100) 93(96)	1384	ND	0.18 (0.02)	0.19 (0.02)	0.16 (0.01)	0.13 (0.03)	0.15 (0.02)	0.18 (0.03)
25	β-elemene	1391[a,b,c]	1389[c]	1.29 (0.04)	1.29 (0.03)	1.31 (0.03)	1.26 (0.02)	1.28 (0.05)	1.27 (0.04)
26	204[M+](13) 157(100) 185(84)	1398	ND	0.25 (0.02)	0.21 (0.01)	0.22 (0.01)	0.19 (0.02)	0.17 (0.04)	0.19 (0.03)
27	204[M+](13) 157(100) 185(84)	1417	ND	0.30 (0.02)	0.32 (0.02)	0.32 (0.02)	0.35 (0.04)	0.39 (0.03)	0.36 (0.02)
28	204[M+](19) 135(100) 105(82)	1423	ND	0.18 (0.01)	0.16 (0.01)	0.17 (0.01)	0.14 (0.02)	0.15 (0.04)	0.16 (0.03)
29	204[M+](9) 91(100) 105(93)	1425	ND	0.04 (0.01)	0.03 (0.01)	0.03 (0.01)	0.02 (0.01)	0.03 (0.01)	0.02 (0.01)
30	(-)-aristolene	1429[a,b,c,d]	1428[a]	1.21 (0.03)	1.23 (0.04)	1.23 (0.04)	1.24 (0.04)	1.19 (0.03)	1.22 (0.03)
31	204[M+](9) 107(100) 79(43)	1432	ND	0.15 (0.02)	0.11 (0.02)	0.11 (0.01)	0.09 (0.02)	0.10 (0.02)	0.13 (0.02)
32	γ-maaliene	1435[a,b]	1427[a]	0.19 (0.02)	0.24 (0.04)	0.26 (0.02)	0.26 (0.02)	0.27 (0.03)	0.26 (0.02)
33	α-maaliene	1443[a,b]	1442[a]	0.33 (0.03)	0.23 (0.03)	0.20 (0.02)	0.27 (0.03)	0.19 (0.04)	0.22 (0.03)
34	aromandendrene	1445[a,b]	1447[a]	3.47 (0.02)	3.28 (0.04)	3.27 (0.04)	3.36 (0.05)	3.32 (0.03)	3.42 (0.04)
35	selina-5,11-diene	1447[a,b]	1454[a]	0.32 (0.02)	0.29 (0.02)	0.26 (0.02)	0.27 (0.02)	0.30 (0.02)	0.31 (0.02)
36	dehydroaromadendrene	1456[c]	1460[c]	1.23 (0.05)	1.26 (0.05)	1.16 (0.05)	1.22 (0.03)	1.14 (0.03)	1.16 (0.04)
37	1,2,9,10-tetradehydroaristolane	1461	ND	0.52 (0.02)	0.50 (0.04)	0.49 (0.03)	0.47 (0.03)	0.49 (0.02)	0.52 (0.03)
38	204[M+](15) 91(100) 105(84)	1465	ND	0.31 (0.02)	0.32 (0.02)	0.37 (0.04)	0.32 (0.02)	0.34 (0.03)	0.32 (0.03)
39	204[M+](18) 128(100) 143(95)	1469	ND	0.29 (0.01)	0.38 (0.03)	0.40 (0.02)	0.33 (0.02)	0.31 (0.02)	0.36 (0.02)
40	γ-gurjunene	1474[c,d]	1475[c]	0.28 (0.01)	0.33 (0.04)	0.32 (0.03)	0.29 (0.03)	0.27 (0.03)	0.28 (0.02)
41	γ-muurolene	1477[c]	1478[c]	0.11 (0.02)	0.13 (0.02)	0.08 (0.01)	0.14 (0.02)	0.16 (0.01)	0.09 (0.01)
42	δ-selinene	1488[c]	1492[c]	1.07 (0.03)	0.97 (0.04)	0.99 (0.01)	0.96 (0.02)	1.05 (0.05)	1.01 (0.05)
43	ledene	1492[a,b,c]	1496[c]	1.34 (0.03)	1.43 (0.03)	1.42 (0.02)	1.36 (0.02)	1.39 (0.03)	1.35 (0.04)
44	204[M+](38) 105(100) 93(96)	1495	ND	0.06 (0.01)	0.09 (0.01)	0.09 (0.01)	0.09 (0.03)	0.08 (0.01)	0.06 (0.01)
45	bicyclogermacrene	1499[a,b,c]	1500[c]	7.56 (0.04)	7.76 (0.03)	7.67 (0.06)	7.68 (0.02)	7.56 (0.04)	7.43 (0.05)
46	204[M+](19) 93(100) 91(95)	1505	ND	0.20 (0.02)	0.16 (0.01)	0.14 (0.02)	0.12 (0.01)	0.16 (0.02)	0.12 (0.01)
47	202[M+](25) 133(100) 91(89)	1509	ND	0.23 (0.03)	0.17 (0.01)	0.23 (0.04)	0.24 (0.01)	0.23 (0.04)	0.18 (0.01)
48	206[M+](14) 191(100) 57(38)	1514	ND	0.18 (0.02)	0.14 (0.01)	0.09 (0.02)	0.13 (0.02)	0.12 (0.01)	0.17 (0.02)
49	202[M+](33) 131(100) 145(53)	1518	ND	0.16 (0.02)	0.21 (0.02)	0.22 (0.02)	0.22 (0.03)	0.25 (0.02)	0.20 (0.03)
50	δ-cadinene	1524[a,b,c]	1522[c]	0.33 (0.02)	0.32 (0.02)	0.34 (0.02)	0.34 (0.02)	0.36 (0.03)	0.29 (0.02)
51	204[M+](5) 91(100) 131(95)	1530	ND	0.08 (0.01)	0.09 (0.01)	0.10 (0.01)	0.07 (0.01)	0.11 (0.01)	0.09 (0.01)
52	200[M+](54) 185(100) 143(91)	1535	ND	0.19 (0.02)	0.17 (0.01)	0.23 (0.02)	0.19 (0.01)	0.24 (0.01)	0.17 (0.03)
53	4,5,9,10-dehydro-isolongifolene	1544[a,b]	1544[a]	8.69 (0.05)	8.48 (0.04)	8.75 (0.05)	8.59 (0.05)	8.61 (0.04)	8.33 (0.04)
54	202[M+](4) 128(100) 157(95)	1547	ND	1.47 (0.03)	1.52 (0.03)	1.47 (0.03)	1.53 (0.04)	1.55 (0.04)	1.49 (0.03)

55	200[M+](8) 171(100) 186(79)	1551	ND	0.21 (0.02)	0.20 (0.03)	0.19 (0.02)	0.22 (0.01)	0.24 (0.02)	0.18 (0.02)
56	200[M+](91) 129(100) 157(88)	1556	ND	0.12 (0.02)	0.14 (0.02)	0.08 (0.01)	0.09 (0.02)	0.08 (0.01)	0.09 (0.01)
57	204[M+](8) 143(100) 157(98)	1559	ND	0.09 (0.01)	0.06 (0.01)	0.06 (0.01)	0.08 (0.01)	0.06 (0.03)	0.06 (0.01)
58	204[M+](82) 173(100) 189(94)	1563	ND	1.59 (0.03)	1.62 (0.05)	1.62 (0.04)	1.57 (0.02)	1.58 (0.02)	1.55 (0.02)
59	palustrol	1567[c]	1567[c]	9.90 (0.03)	9.85 (0.05)	9.85 (0.05)	9.83 (0.04)	9.86 (0.06)	9.83 (0.05)
60	200[M+](11) 79(100) 93(95)	1570	ND	0.55 (0.04)	0.49 (0.02)	0.49 (0.03)	0.48 (0.02)	0.48 (0.04)	0.52 (0.03)
61	204[M+](31) 81(100) 109(88)	1573	ND	0.91 (0.03)	0.89 (0.03)	0.91 (0.02)	0.94 (0.03)	0.93 (0.03)	0.92 (0.02)
62	spathulenol	1576[a,b,c]	1577[c]	5.13 (0.03)	5.01 (0.04)	5.06 (0.04)	4.97 (0.06)	5.01 (0.03)	5.06 (0.05)
63	200[M+](56) 185(100) 143(63)	1581	ND	5.23 (0.05)	5.48 (0.04)	5.44 (0.03)	5.39 (0.03)	5.28 (0.04)	5.38 (0.04)
64	202[M+](4) 91(100) 79(82)	1587	ND	0.47 (0.03)	0.46 (0.03)	0.43 (0.02)	0.49 (0.02)	0.49 (0.02)	0.44 (0.02)
65	globulol	1599[a,b,c,d]	1590[c]	3.13 (0.02)	3.09 (0.03)	3.08 (0.05)	3.07 (0.03)	3.09 (0.03)	3.13 (0.03)
66	200[M+](8) 198(100) 183(84)	1605	ND	0.23 (0.01)	0.27 (0.02)	0.23 (0.02)	0.22 (0.03)	0.26 (0.02)	0.31 (0.02)
67	220[M+](2) 145(100) 200(93)	1609	ND	2.15 (0.02)	2.14 (0.03)	2.09 (0.04)	2.08 (0.04)	2.22 (0.05)	2.14 (0.06)
68	(+)-bisabola-2,10-diene[1,9]oxide	1615[a,b]	1596[a]	0.19 (0.02)	0.17 (0.02)	0.19 (0.02)	0.17 (0.02)	0.14 (0.03)	0.22 (0.03)
69	208[M+](3) 95(100) 85(95)	1621	ND	1.46 (0.03)	1.43 (0.04)	1.19 (0.06)	1.16 (0.03)	1.17 (0.04)	1.26 (0.04)
70	ledene oxide-(II)	1631[a,b]	1631[a]	0.21 (0.01)	0.25 (0.01)	0.19 (0.01)	0.27 (0.02)	0.19 (0.03)	0.24 (0.03)
71	isospathulenol	1635[a,b]	1633[a]	0.96 (0.02)	0.87 (0.03)	0.89 (0.02)	0.98 (0.03)	1.01 (0.03)	0.88 (0.02)
72	220[M+](18) 91(100) 105(83)	1639	ND	1.54 (0.04)	1.61 (0.02)	1.53 (0.05)	1.54 (0.02)	1.57 (0.04)	1.64 (0.04)
73	cubenol	1642[a,b,c,d]	1645[c]	0.42 (0.03)	0.51 (0.03)	0.42 (0.03)	0.39 (0.02)	0.39 (0.03)	0.49 (0.02)
74	220[M+](21) 91(100) 105(82)	1651	ND	0.09 (0.01)	0.08 (0.02)	0.07 (0.01)	0.08 (0.01)	0.10 (0.02)	0.07 (0.02)
75	222[M+](3) 179(100) 121(92)	1655	ND	0.05 (0.01)	0.06 (0.01)	0.04 (0.01)	0.04 (0.02)	0.03 (0.01)	0.04 (0.01)
76	germacra-4(15),5,10(14)-trien-1-α-ol	1660[c]	1685[c]	0.92 (0.03)	0.96 (0.03)	0.98 (0.02)	1.06 (0.03)	0.98 (0.02)	0.96 (0.02)
77	216[M+](31) 145(100) 91(97)	1699	ND	0.68 (0.02)	0.62 (0.04)	0.57 (0.03)	0.55 (0.05)	0.67 (0.03)	0.58 (0.04)
78	1,4-dimethyl-7-(1-methylethyl)-azulene	1790[c]	1779[c]	3.45 (0.04)	3.34 (0.02)	3.38 (0.03)	3.41 (0.06)	3.42 (0.03)	3.24 (0.03)
79	14-hydroxy-δ-cadinene	1797[c]	1803[c]	0.36 (0.02)	0.43 (0.03)	0.37 (0.02)	0.31 (0.03)	0.27 (0.02)	0.38 (0.02)
	Total			98.73 (1.87)	98.48 (2.05)	97.35 (1.98)	97.81 (2.05)	97.71 (2.13)	97.56 (2.14)
	% Identified			77.43 (1.15)	76.96 (1.28)	76.37 (1.20)	77.08 (1.25)	76.49 (1.23)	76.64 (1.31)
	Including:								
	Aliphatics			3.02 (0.17)	3.05 (0.21)	3.03 (0.19)	3.07 (0.20)	2.95 (0.18)	3.02 (0.21)
	Aromatics			5.23 (0.16)	5.19 (0.18)	5.00 (0.17)	5.07 (0.18)	5.08 (0.14)	5.14 (0.17)
	Monoterpene hydrocarbons			0.05 (0.01)	0.04 (0.01)	0.04 (0.01)	0.04 (0.01)	0.06 (0.01)	0.06 (0.01)
	Monoterpenoide hydrocarbons			0.07 (0.01)	0.06 (0.01)	0.04 (0.01)	0.04 (0.01)	0.05 (0.01)	0.05 (0.01)
	Sesquiterpene hydrocarbons			47.84 (0.59)	47.48 (0.60)	47.23 (0.56)	47.81 (0.57)	47.41 (0.61)	47.18 (0.64)
	Sesquiterpenoide hydrocarbons			21.22 (0.21)	21.14 (0.27)	21.03 (0.26)	21.05 (0.28)	20.94 (0.28)	21.19 (0.27)

- less than 0.01%. * The names of terpenes and terpenoids according to IUPAC terminology are given in Table S5. ** Retention index on Quadex 007-5MS column. *** Literature retention index. ND No data. **** For abbreviations of samples see Table 1. ( ) standard deviation. Identification of compounds by MS databases ([a] - NIST 2011, [b] - NIST Chemistry WebBook, [c] - Adams 4 Library, [d] - Pherobase).

Table S5. IUPAC name for detected terpenes and terpenoids.

No.*	Compounds	
	**Name**	**IUPAC name**
6	α-pinene	(1*R*,5*R*)-2,6,6-trimetylobicyklo[3.1.1]hept-2-en
15	β-cyclocitral	2,6,6-trimethylcyclohex-1-ene-1-carbaldehyde
17	bicycloelemene	3-ethenyl-3,7,7-trimethyl-2-prop-1-en-2-ylbicyclo[4.1.0]heptane
18	δ-elemene	(3*R*,4*R*)-1-isopropyl-4-methyl-3-(prop-1-en-2-yl)-4-vinylcyclohex-1-ene
23	anastreptene	(1*R*,2*S*,4*R*)-3,3,7,11-tetramethyltetracyclo[5.4.0.$0^{1,8}$.$0^{2,4}$]undec-10-ene
25	β-elemene	(1*S*,2*S*,4*R*)-1-methyl-2,4-di(prop-1-en-2-yl)-1-vinylcyclohexane
30	(-)-aristolene	1,1,7,7a-tetramethyl-1H,1aH,2H,4H,5H,6H,7H,7aH,7bH-cyclopropa[a]naphthalene
32	γ-maaliene	(1*aS*,3*aR*,7*aS*,7*bS*)-1,1,3*a*-trimethyl-7-methylidene-1*a*,2,3,4,5,6,7*a*,7*b*-octahydrocyclopropa[a]naphthalene
33	α-maaliene	(1*aS*,3*aR*,7*aR*,7*bS*)-1,1,3*a*,7-tetramethyl-2,3,4,5,7*a*,7*b*-hexahydro-1*aH*-cyclopropa[a]naphthalene
34	aromandendrene	1,1,7-trimethyl-4-methylidene-2,3,4*a*,5,6,7,7*a*,7*b*-octahydro-1*aH*-cyclopropa[e]azulene
35	selina-5,11-diene	(4*aR*)-1,4*a*-dimethyl-7-prop-1-en-2-yl-2,3,4,5,6,7-hexahydro-1*H*-naphthalene
36	dehydroaromadendrene	1,1,7-trimethyl-4-methylidene-4*a*,5,6,7,7*a*,7*b*-hexahydro-1*aH*-cyclopropa[e]azulene
37	1,2,9,10-tetradehydroaristolane	1,1,7,7a-tetramethyl-1a,2,6,7,7a,7b-hexahydro-1H-cyclopropa[a]naphthalene
40	γ-gurjunene	1,4-dimethyl-7-prop-1-en-2-yl-1,2,3,3*a*,4,5,6,7-octahydroazulene
41	γ-muurolene	7-methyl-4-methylidene-1-(propan-2-yl)-1,2,3,4,4a,5,6,8a-octahydronaphthalene
42	δ-selinene	4,8*a*-dimethyl-6-propan-2-yl-2,3,7,8-tetrahydro-1*H*-naphthalene
43	ledene	(1S,2R,4R,11R)-3,3,7,11-tetramethyltricyclo[6.3.0.02.4]undec-7-ene
45	bicyclogermacrene	(1*S*,2*E*,6*E*,10*R*)-3,7,11,11-tetramethylbicyclo[8.1.0]undeca-2,6-diene
50	δ-cadinene	(1*S*,8*aR*)-4,7-dimethyl-1-propan-2-yl-1,2,3,5,6,8*a*-hexahydronaphthalene
53	4,5,9,10-dehydro-isolongifolene	2,2,7,7-tetramethyltricyclo[6.2.1.$0^{1,6}$]undeca-3,5,9-triene
59	palustrol	1,1,4,7-tetramethyl-2,3,4,5,6,7,7*a*,7*b*-octahydro-1*aH*-cyclopropa[h]azulen-4*a*-ol
62	spathulenol	(1*aR*,4*aR*,7*S*,7*aR*,7*bR*)-1,1,7-trimethyl-4-methylidene-1*a*,2,3,4*a*,5,6,7*a*,7*b*-octahydrocyclopropa[h]azulen-7-ol
65	globulol	(1*aR*,4*R*,4*aR*,7*R*,7*aS*,7*bS*)-1,1,4,7-tetramethyl-2,3,4*a*,5,6,7,7*a*,7*b*-octahydro-1*aH*-cyclopropa[e]azulen-4-ol
68	(+)-bisabola-2,10-diene[1,9]oxide	4,7-dimethyl-2-(2-methylprop-1-enyl)-3,4,4a,5,6,8a-hexahydro-2H-chromene
70	ledene oxide-(II)	3,7,7,10-tetramethyl-2-oxatetracyclo[7.3.0.$0^{1,3}$.$0^{6,8}$]dodecane
71	isospathulenol	(1*aR*,7*S*,7*aS*,7*bR*)-1,1,4,7-tetramethyl-2,3,5,6,7*a*,7*b*-hexahydro-1*aH*-cyclopropa[h]azulen-7-ol
73	cubenol	(1*S*,4*R*,4*aR*,8*aR*)-4,7-dimethyl-1-propan-2-yl-2,3,4,5,6,8*a*-hexahydro-1*H*-naphthalen-4*a*-ol
76	germacra-4(15),5,10(14)-trien-1-α-ol	(1*S*,5*E*,7*S*)-4,10-dimethylidene-7-propan-2-ylcyclodec-5-en-1-ol
78	1,4-dimethyl-7-(1-methylethyl)-azulene	1,4-dimethyl-7-(propan-2-yl)azulene
79	14-hydroxy-δ-cadinene	(8-isopropyl-5-methyl-3,4,6,7,8,8a-hexahydro-2-naphthalenyl)methanol

* Numbering of compounds as in Tables 2a-7 and Table S2a-S4b.

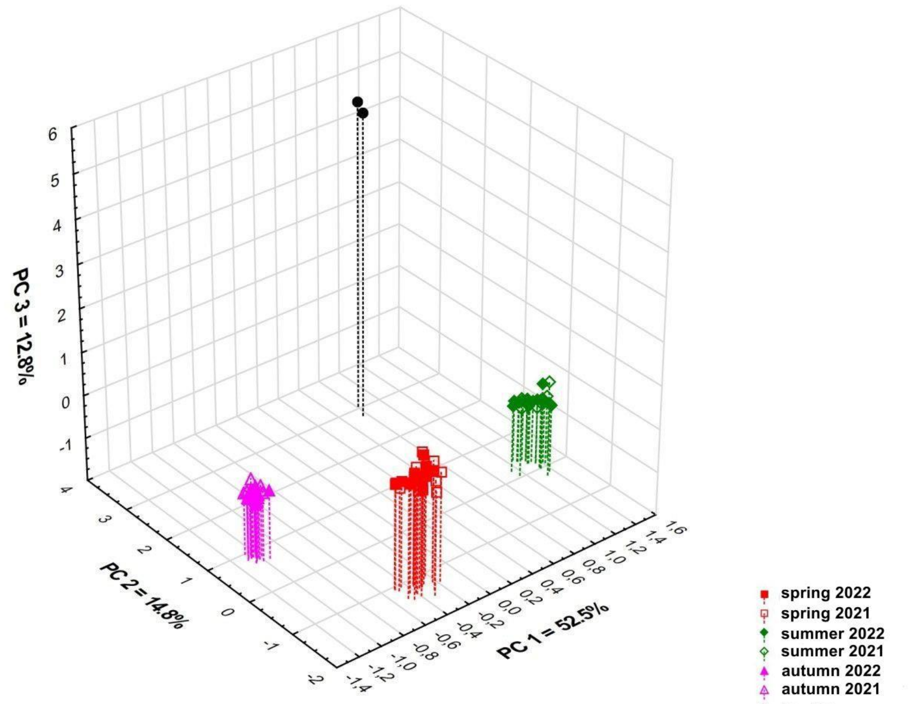

Figure S7. Three-dimensional PCA scatter plot based on all 79 detected compounds in samples of *Calypogeia integristipula* collected in spring, summer, autumn in 2022 and 2021 and *in vitro*. The percentage of explained variance (R^2X) is 52.5% for PC1, 14.8% for PC2, 12.8% for PC3, and predictive ability (Q^2) is 48.2%, 16.7%, and 25.2%, respectively.

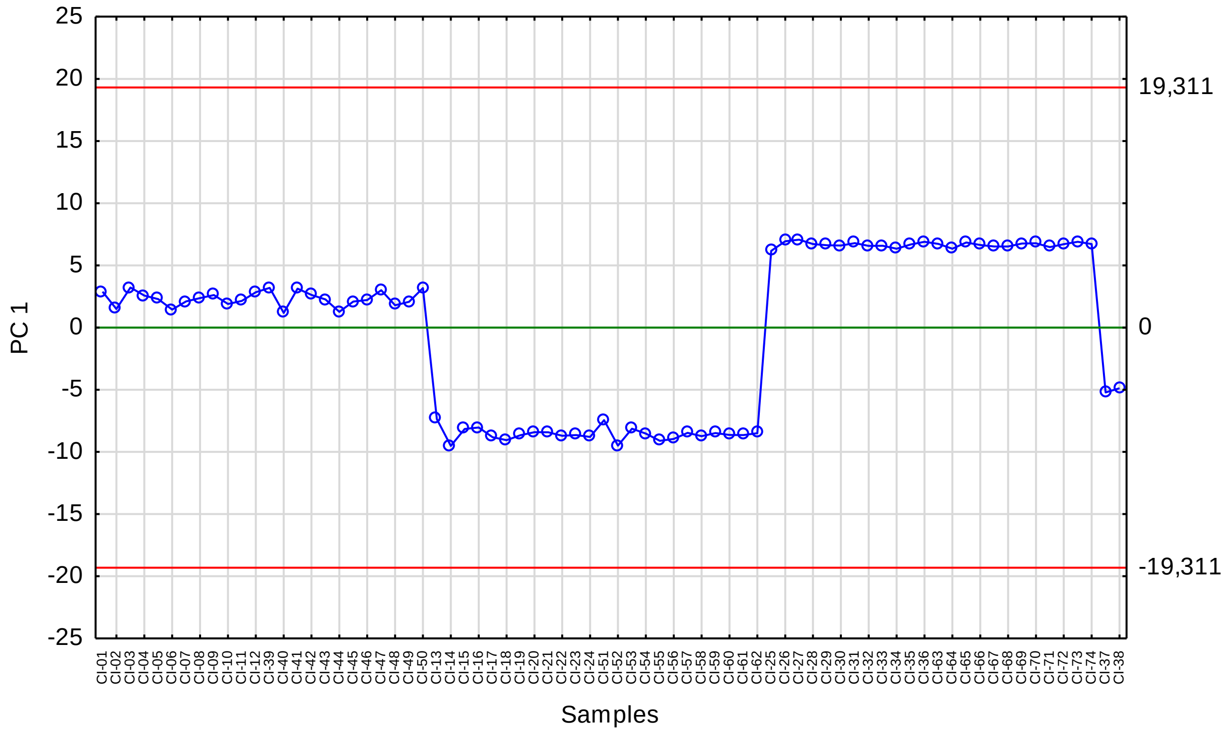

Figure S8. Line plot of the component PC1 for the examined samples of *Calypogeia integristipula* collected in 2021 and 2022 and *in vitro* based on all 79 detected compounds. The red lines represent +/-3.00 standard deviations. SD: 6.637 (Figure S7).

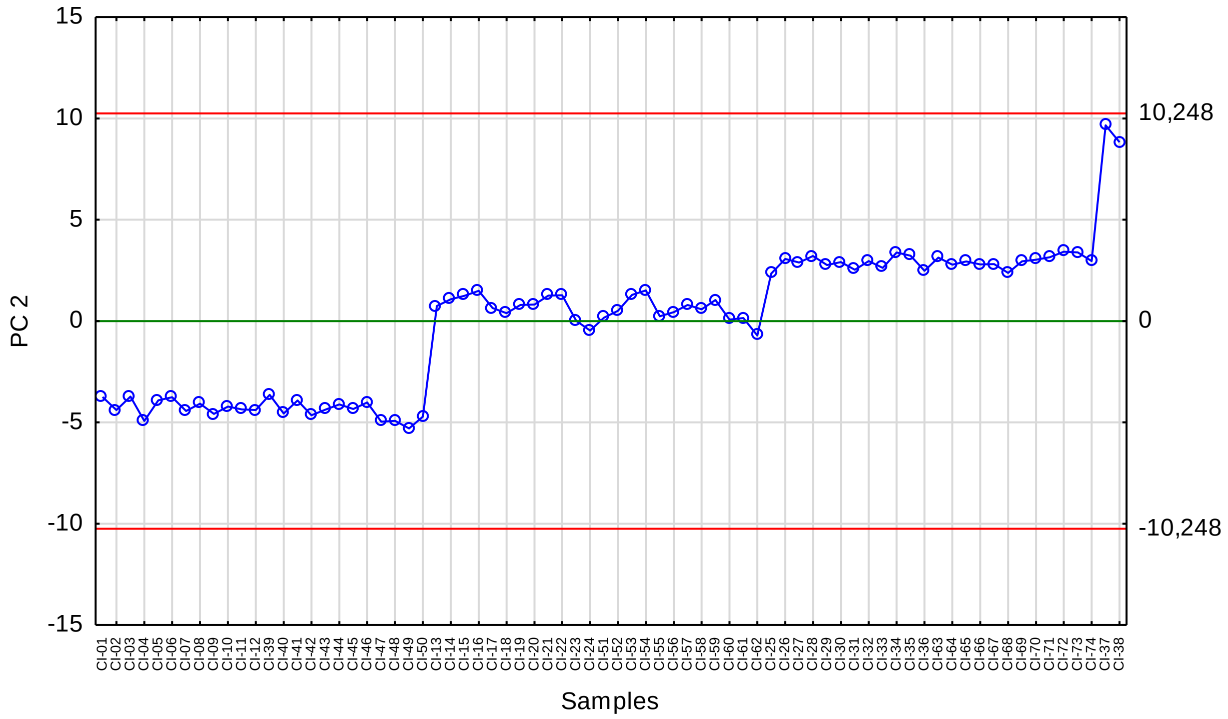

Figure S9. Line plot of the component PC2 for the examined samples of *Calypogeia integristipula* collected in 2021 and 2022 and *in vitro* based on all 79 detected compounds. The red lines represent +/-3.00 standard deviations. SD: 3.416 (Figure S7).

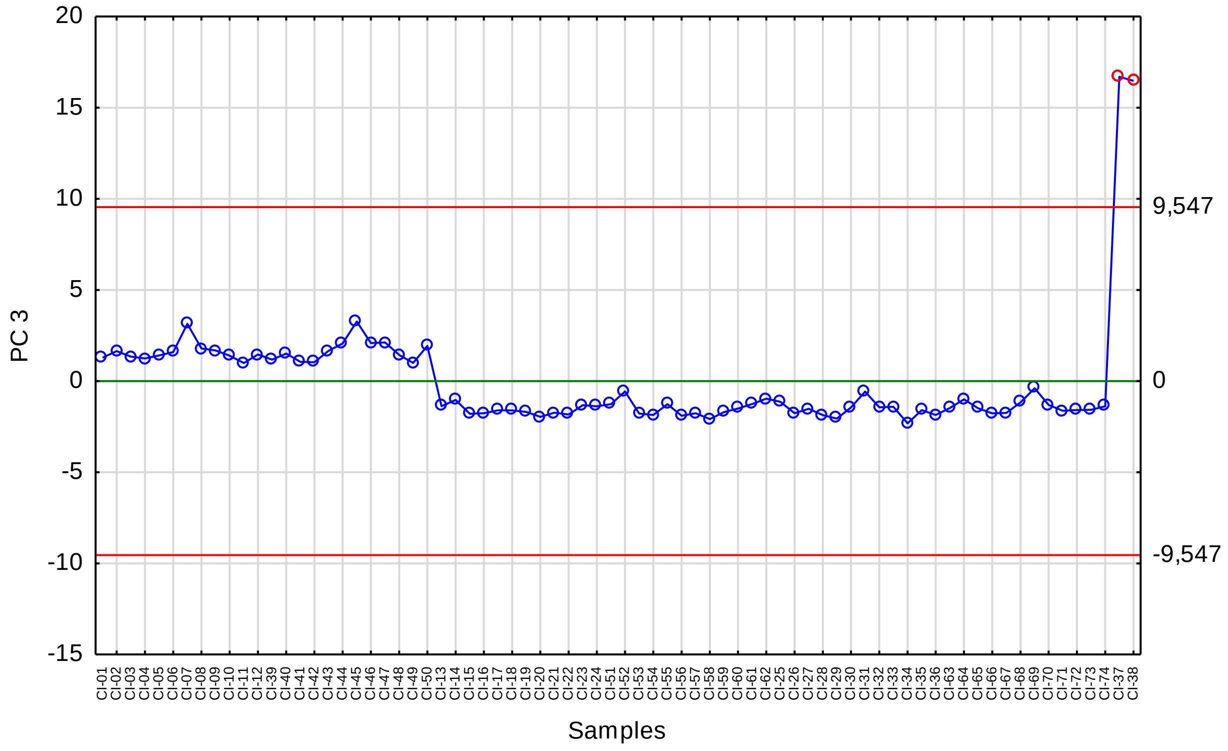

Figure S10. Line plot of the component PC3 for the examined samples of *Calypogeia integristipula* collected in 2021 and 2022 and *in vitro* based on all 79 detected compounds. The red lines represent +/-3.00 standard deviations. SD: 3.182 (Figure S7).

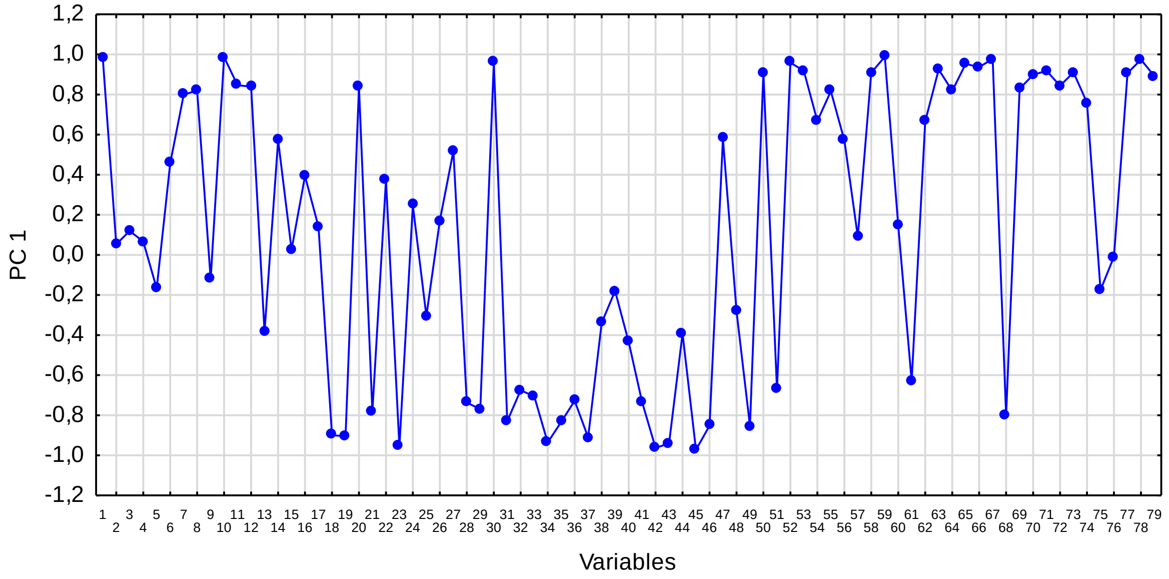

Figure S11. Linear plot of the lodgings for the first principal component PC1 (Figure S7).

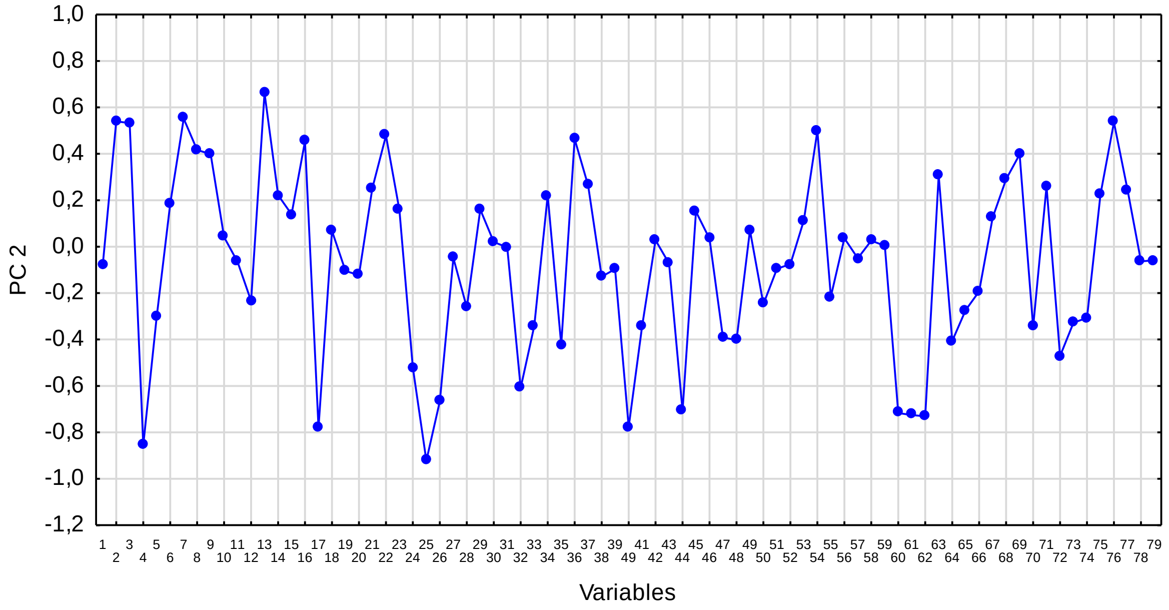

Figure S12. Linear plot of the lodgings for the second principal component PC2 (Figure S7).

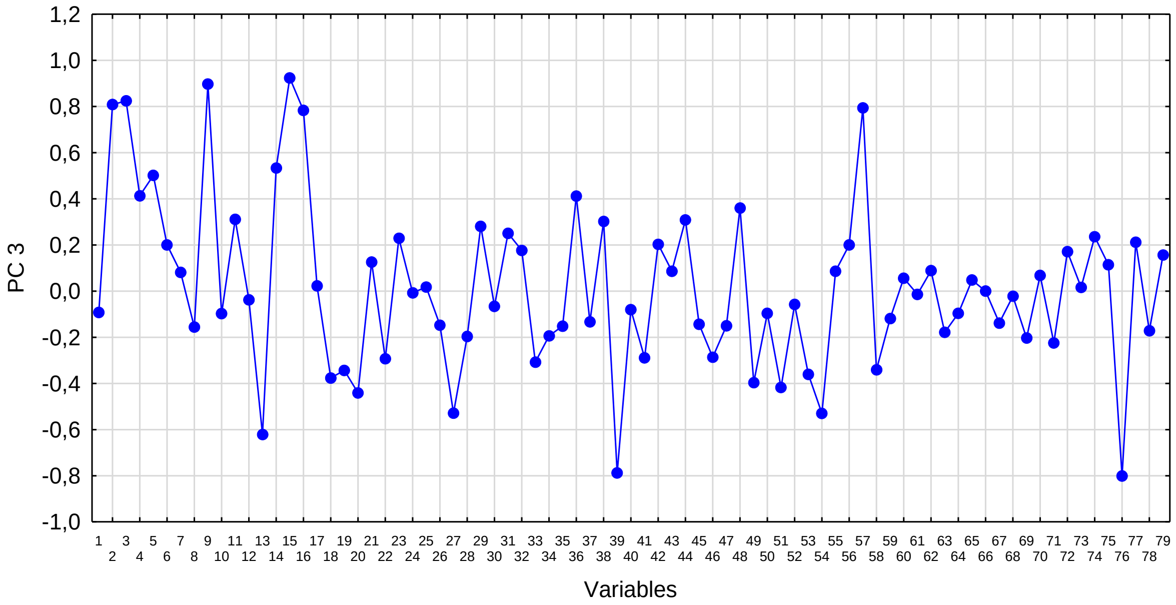

Figure S13. Linear plot of the lodgings for the third principal component PC3 (Figure S7).
